# Supplementary material for: Safety and immunogenicity following a homologous booster dose of CoronaVac in children and adolescents
Source: Nat Commun. 2022 Nov 14;13:6952. doi: 10.1038/s41467-022-34280-y (PMC9663200; doi:10.1038/s41467-022-34280-y)
Supplement: Supplementary file 1 — Supplementary Information [file 41467_2022_34280_MOESM1_ESM.pdf]

Contents

1. Immunogenic Detection Method 2

2. Serious adverse events 4

3. Immunogenicity results 5

Table 3-1 Immunogenicity results of neutralising antibody to live prototype SARS-CoV-2 induced by CoronaVac 5

Table 3-2 Comparisons of seropositivity rates and GMTs/GMIs of neutralizing antibodies to SARS-CoV-2 Omicron variant in different age groups at day 28 after the third dose 6

Fig. 1 Reduction of cross-neutralizing to the Omicron among the three age groups 7

4. Study protocol 8

1. Immunogenic Detection Method

Detection Method of Neutralization Potency against the live prototype SARS-CoV-2 and the Omicron variant

Micro cytopathic effect assay was adopted.

Serum treatment: All serum samples were inactivated at 56°C in a water bath for 30 minutes.

Medium addition: the cell maintenance medium was added to the cell control group at 100 μl/well, and 50 μl/well of maintenance medium was supplemented to the to-be-tested serum group, virus back titration group and positive control group from the second dilution.

Dilution of the serum sample: The serum was diluted four-fold (60 μl sample + 180 μl maintenance medium) with cell maintenance medium (2% newborn calf serum-199 (2% sodium hydrogen carbonate) cell maintenance medium). The diluted serum was added to the cell plate at 100 μl/well, and each sample was diluted to 2 wells in parallel. 50 μl of the mixture in the first dilution was pipetted into the next dilution, and the mixture was pipetted up and down for 8-10 times. The mixture was diluted to the appropriate dilution range by this method, and 50 μl of the last dilution was discarded, and 50 μl of the diluted sample was retained in each well.

Dilution of the virus for neutralization: The SAR-CoV-2 used for neutralization was diluted to 100CCID$_{50}$/0.05 ml by titer.

Neutralization: Serum of different dilutions was mixed with 100CCID$_{50}$/0.05 ml virus liquid in equal volume (50 μl+50 μl), respectively, and then incubated in an incubator at 36.5°C, 5%CO_2 for 2h.

Experimental control: Negative serum control, positive serum control, serum sample and cell control were set simultaneously.

Virus Back Titration: The virus suspension diluted to 100 CCID$_{50}$/0.05 mL was diluted via ten-fold serial dilution, i.e. diluted to 10 CCID50/0.05 ml, 1 CCID$_{50}$/0.05 mL and 0.1 CCID50/0.05 ml, and added to the 96-well cell plate respectively, 12 well per dilution and 50 μl per well, then 50 μl of cell maintenance medium was added to each well, and the plate was incubated in an incubator at 36.5°C, 5% CO2 for 5 days.

Cell Inoculation and Culture: After incubation, 100μL of Vero cell suspension (cell concentration: 1.0-2.0×10^5 cell/ml) was added to each well, and then incubated in an incubator at 36.5°C, 5% CO_2 for 5 days.

Interpretation of the Results: It was observed for the cytopathic effect after cultured for 3-5 days, and the neutralizing antibody titer of the to-be-tested serum sample was determined according to the observation results of the cytopathic effect (CPE) on the 5th day. The reciprocal of the highest serum dilution without cytopathic effects the end titer. When 1 of the 2 wells of the highest dilution serum shows CPE, while the other does not, the reciprocal of the dilution should be the neutralizing antibody titer of the serum specimen; the reciprocal of the mean dilution of the two wells should be the neutralizing antibody titer of the serum specimen when the 2 wells with the highest

dilution are completely pathological while the adjacent 2 wells with low dilution are not pathological completely; when 1 of two adjacent wells is pathological while the other not, the reciprocal of the average dilutions of 2 wells should be the neutralizing antibody titer of the serum specimen. For example, 2 wells with high dilution of 1:16 have a complete CPE, while the adjacent 2 wells with low dilution of 1:8 have no CPE; or in 2 adjacent wells with dilutions of 1:8 and 1:16, one has a CPE, while the other does not. In this case, the reciprocal 12 of the average dilutions of 2 wells is the neutralizing antibody titer of the serum.

2. Serious adverse events

As of February 10, 2022, only one serious adverse event has been reported, which was considered unrelated to vaccination. The case narrative of this serious adverse events as follows:

This participant received the first dose of investigational vaccine on Dec 13, 2020 and received the second dose on Jan 17, 2021 and received the third dose on Nov 13, 2021. On Nov 28, 2021, This participant had fever of 38.0°C, congestion in the pharynx and enlarged tonsils bilaterally, and the symptoms did not improve after oral drug treatment. This participant was then hospitalized on Nov 29, 2022. During hospitalization, this participant had blood tests and chest X-ray test: WBC.4.03$^{10\hat{}9/L}$, PLT 207.00$^{10\hat{}9/L}$, NEUT% 43.90%, LYMPH% 31.80%, CRP<10mg/L, and no abnormalities observed in X-ray. After admission, this participant was given anti-infection (Piperacillin sodium), and Tanreqing injection. This participant recovered and discharged on Dec 6, 2021.

Causality: According to this participant’s symptoms and related examinations, the serious adverse event was clearly diagnosed as “acute tonsillitis”. It was effective after symptomatic treatment. In addition, the inactivated vaccine did not cause infectious diseases. Therefore, this serious adverse event was considered possibly unrelated to vaccination.

3 Immunogenicity results

Table 3-1 Immunogenicity results of neutralising antibody to live prototype SARS-CoV-2 induced by CoronaVac

Time	Characteristics	Cohort 1			Cohort 2		
		1.5 ug group	3.0 ug group	p value	1.5 ug group	3.0 ug group	p value
**Before vaccination**	Seropositive n/N (%)	0/86 (0.0)	0/85 (0.0)	1.000	0/86 (0.0)	0/87 (0.0)	1.000
	(95%CI)	(0.0-4.2)	(0.0-4.3)		(0.0-4.2)	(0.0-4.2)	
	GMT	2.0	2.0	1.000	2.0	2.0	1.000
	(95%CI)	(2.0-2.0)	(2.0-2.0)		(2.0-2.0)	(2.0-2.0)	
**28 days after Dose 2**	Seropositive n/N (%)	81/86 (94.2)	83/83 (100.0)	0.059	85/86 (98.8)	87/87 (100.0)	0.497
	(95%CI)	(87.0-98.1)	(95.7-100.0)		(93.7-99.97)	(95.9-100.0)	
	GMT	81.8	138.1	0.001	90.2	147.4	0.001
	(95%CI)	(64.3-104.1)	(111.9-170.3)		(71.2-114.2)	(123.4-176.2)	
	GMFR*	40.9	69.0	0.001	45.1	73.7	0.001
	(95%CI)	(32.1-52.0)	(56.0-85.2)		(35.6-57.1)	(61.7-88.1)	
**3 months after Dose 2**	Seropositive n/N (%)	85/85 (98.8)	85/85 (100.0)	1.000	NA	NA	NA
	(95%CI)	(93.7-99.97)	(95.8-100.0)		NA	NA	
	GMT	67.4	110.3	<0.001	NA	NA	NA
	(95%CI)	(55.0-82.5)	(90.4-134.6)		NA	NA	
	GMFR*	33.7	55.1	<0.001	NA	NA	NA
	(95%CI)	(27.5-41.3)	(45.2-67.3)		NA	NA	
**6 months after Dose 2**	Seropositive n/N (%)	NA	NA	NA	71/82 (86.6)	82/86 (95.4)	0.047
	(95%CI)	NA	NA		(77.3-93.1)	(88.5-98.7)	
	GMT	NA	NA	NA	22.1	27.2	0.127
	(95%CI)	NA	NA		(18.1-26.9)	(22.7-32.5)	
	GMFR*	NA	NA	NA	11.1	13.6	0.127
	(95%CI)	NA	NA		(9.1-13.5)	(11.3-16.3)	
**10 months after Dose 2**	Seropositive n/N (%)	60/86 (69.8)	74/85 (87.1)	0.006	NA	NA	NA
	(95%CI)	(58.9-79.2)	(78.0-93.4)		NA	NA	
	GMT	12.7	20.8	0.002	NA	NA	NA
	(95%CI)	(10.4-15.5)	(16.5-26.1)		NA	NA	
	GMFR*	6.4	10.4	0.002	NA	NA	NA
	(95%CI)	(5.2-7.8)	(8.3-13.1)		NA	NA	
**12 months after Dose 2**	Seropositive n/N (%)	NA	NA	NA	66/86 (76.7)	81/87 (93.1)	0.003
	(95%CI)	NA	NA		(66.4-85.2)	(85.6-97.4)	
	GMT	NA	NA	NA	16.4	21.7	0.091
	(95%CI)	NA	NA		(13.0-20.6)	(18.1-26.0)	
	GMFR*	NA	NA	NA	8.4	10.9	0.091
	(95%CI)	NA	NA		(6.6-10.6)	(9.0-13.0)	
**28 days after Dose 3**	Seropositive n/N (%)	85/85 (100.0)	85/85 (100.0)	1.000	85/85 (100.0)	82/82 (100.0)	1.000
	(95%CI)	(95.8-100.0)	(95.8-100.0)		(95.8-100.0)	(95.6-100.0)	
	GMT	597.7	681.0	0.468	462.4	745.2	0.007
	(95%CI)	(452.4-789.6)	(545.2-850.7)		(364.4-586.6)	(577.0-962.3)	
	GMFR#	46.4	32.8	0.072	28.0	33.3	0.350
	(95%CI)	(35.3-60.9)	(25.1-42.8)		(22.0-35.6)	(25.0-44.4)	

GMT=geometric mean titre; GMFR =geometric mean fold rise. GMFR*: taking before vaccination as baseline, GMFR#: taking pre-booster as baseline.

Data are n/N (%) (95% CI) unless otherwise stated. Comparison between groups was conducted by group t-test with log-transformation. Pearson's chi-squared test and Fisher's exact test were used to analyses categorical outcome. Two-sided tests were used.

Table 3-2 Comparisons of seropositivity rates and GMTs/GMFR of neutralising antibodies to SARS-CoV-2 Omicron variant in different age groups at day 28 after the third dose

Doses	Characteristics	Cohort 1				Cohort 2			
		3-5 years	6-11 years	12-17 years	P value	3-5 years	6-11 years	12-17 years	P value
1.5 ug group	Seropositivity n/N (%)	20/20 (100.0)	29/33 (87.9)	17/32 (53.1)	<0.001	20/20 (100.0)	27/32 (84.4)	20/33 (60.6)	0.001
	(95%CI)	(83.2-100.00)	(71.8-96.6)	(34.7-70.9)		(83.2-100.00)	(67.2-94.7)	(42.1-77.1)	
	GMT	59.1	26.1	12.0	<0.001	50.2	28.3	13.1	0.001
	(95%CI)	(38.6-90.4)	(17.0-40.1)	(7.4-19.2)		(31.1-81.1)	(17.6-45.5)	(8.2-20.9)	
	GMFR	28.0	12.5	6.0	<0.001	18.3	14.2	6.6	0.008
	(95%CI)	(18.0-43.4)	(8.3-18.9)	(3.7-9.6)		(11.3-29.6)	(8.8-22.8)	(4.1-10.5)	
3.0 ug group	Seropositivity n/N (%)	21/21 (100.0)	30/32 (93.8)	26/32 (81.3)	0.075	19/19 (100.0)	32/33 (97.0)	24/30 (80.0)	0.017
	(95%CI)	(83.9-100.0)	(79.2-99.2)	(63.6-92.8)		(82.4-100.00)	(84.2-99.9)	(61.4-92.3)	
	GMT	59.1	35.7	23.4	0.009	66.8	53.4	21.1	<0.001
	(95%CI)	(40.9-85.4)	(24.8-51.5)	(15.1-36.1)		(42.0-106.3)	(36.7-77.6)	(13.1-33.9)	
	GMFR	23.3	17.5	11.4	0.040	30.4	25.3	10.5	0.001
	(95%CI)	(16.9-32.2)	(12.3-24.9)	(7.4-17.6)		(19.8-46.8)	(17.5-36.4)	(6.6-17.0)	

GMT=geometric mean titre. GMFR=geometric mean fold rise, taking pre-booster as baseline.

Data are n/N (%) (95% CI) unless otherwise stated. ANOVA model with log-transformation (per GMT and GMFR as above) was used to detect the difference among groups. Pearson's chi-squared test and Fisher's exact test were used to analyses categorical outcome. Two-sided tests were used.

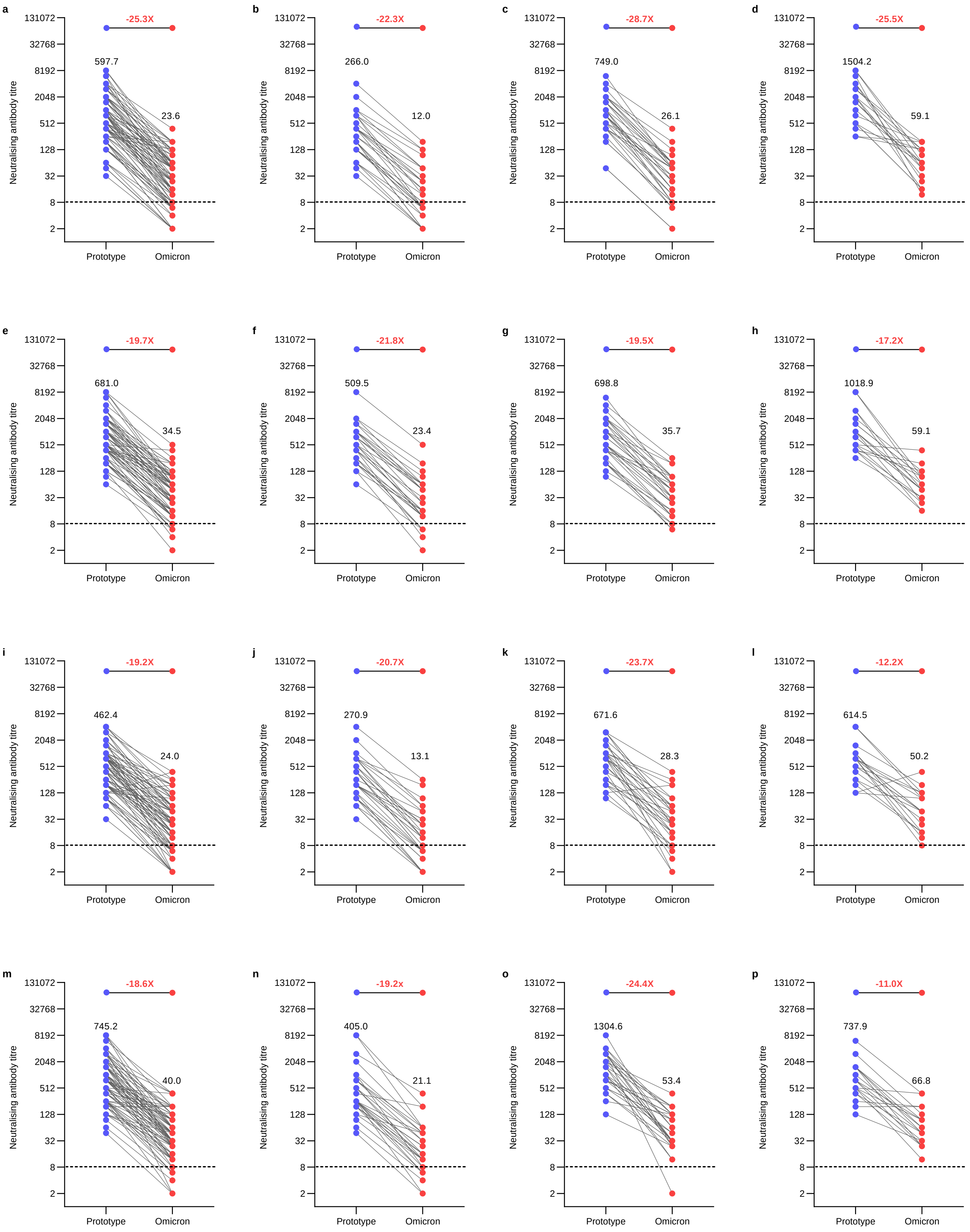

Fig. 1 Reduction of cross-neutralizing to the Omicron among the three age groups

a Paired neutralising antibodies titre against the prototype SARS-COV-2 (blue) and the Omicron (red) in Cohort 1, 1.5μg group, 3-17 years (n=85). **b** Cohort 1, 1.5μg group, 12-17 years (n=32). **c** Cohort 1, 1.5μg group, 6-11 years (n=33). **d** Cohort 1, 1.5μg group, 3-5 years (n=20). **e** Cohort 1, 3.0μg group, 3-17 years (n=85). **f** Cohort 1, 3.0μg group, 12-17 years (n=32). **g** Cohort 1, 3.0μg group, 6-11 years (n=32). **h** Cohort 1, 3.0μg group, 3-5 years (n=21). **i** Cohort 2, 1.5μg group, 3-17 years (n=85). **j** Cohort 2, 1.5μg group, 12-17 years (n=33). **k** Cohort 2, 1.5μg group, 6-11 years (n=32). **l** Cohort 2, 1.5μg group, 3-5 years (n=20). **m** Cohort 2, 3.0μg group, 3-17 years (n=82). **n** Cohort 2, 3.0μg group, 12-17 years (n=30). **o** Cohort 2, 3.0μg group, 6-11 years (n=33). **p** Cohort 2, 3.0μg group, 3-5 years (n=19). Dots are reciprocal neutralising antibodies titres for individuals in the per-protocol population. Numbers above the dots are GMTs. Numbers above the short horizontal lines are reduction folders of cross-neutralizing comparisons between prototype and Omicron. The dotted horizontal line represents the seropositivity threshold (1:8). Titres lower than the limit of detection (1:4) are presented as half the limit of detection. GMT=geometric mean titre.

Project Title: Phase I/II Clinical Trial for COVID-19 Vaccine (Vero Cell), Inactivated in Children and Adolescents

Protocol Title: A Randomized, Double-blind, Placebo-controlled Phase I/II Clinical Trial to Evaluate the Safety and Immunogenicity of COVID-19 Vaccine (Vero Cell), Inactivated in Healthy Children and Adolescents Aged 3-17 Years Old

Product Name: COVID-19 Vaccine (Vero Cell), Inactivated

Sponsor: Sinovac Life Sciences Co., Ltd.

Investigator: Hebei Provincial Center for Disease Control and Prevention

Statistical Organization: Beijing KEY TECH Statistical Technology Co., Ltd.

Protocol No.: PRO-nCOV-1003

Protocol version/date: February 18, 2022

Version No.: 2.6

Protocol approver: Xiaojuan Lian

Signature of approver:

Approval date: MM/DD/YYYY

北京科兴中维生物技术有限公司
SINOVAC LIFE SCIENCES CO., LTD.

Signature of Principal Investigator

I hereby agree to:

- Assume the responsibility for properly instructing the Clinical Research in this region.
- Ensure that the Research is carried out in accordance with the Trial Protocol and standard operating procedure for clinical research.
- Ensure that the personnel involved in the Project have a full knowledge of the research product information, as well as other responsibilities and obligations in connection with the Research as specified in the Trial Protocol.
- Ensure that no change will be made to the Trial Protocol without review and written approval of the Sponsor and the Independent Ethics Committee (IEC), unless it is necessary to eliminate the immediate hazard to the subjects or as required by the registration authority (for example: in terms of administration of the Project).
- I am thoroughly familiar with the methods for properly using the Vaccine as described in the Trial Protocol and have a full knowledge of other information provided by the Sponsor, including but not limited to the following: the current Investigator's Brochure (IB) or equivalent document and Supplementary Documents to the Investigator's Brochure (if any).
- I am familiar with and will comply with the *Good Clinical Practice* (GCP), *Guidelines for Quality Management of Vaccine Clinical Trial (Trial)* and all prevailing regulatory requirements.

Name of Principal Investigator: Zhao Yuliang

Signature:

Date: MM/DD/YYYY

Research Team

Sponsor

Organization name: Sinovac Research & Development Co., Ltd.

Address: Building 1, No. 21, Tianfu Street, Daxing Biomedical Industry Base, Zhongguancun Science Park, Daxing District, Beijing

Contact name: Gao Qiang

Mobile: 13693092396 Fax: 010-62979669 Postcode: 102629

E-mail: gaoq@sinovac.com

Organization Responsible for the Clinical Trial

Organization name: Hebei Provincial Center for Disease Control and Prevention

Address: No. 97 East Huai'an Road, Shijiazhuang City

Specialized department: Institute for Clinical Research of Vaccines

Principal investigator: Zhao Yuliang

Mobile: 13315290538 Fax: 0311-86573212 Postcode: 050021

E-mail: yuliang_zh@163.com

Site Organization of the Clinical Trial

Organization name: Center for Disease Control and Prevention of Zanhuang County

Address: No. 75, East Tanshan Road, Zanhuang County

Name of person in charge: Jiao Wenbin

Mobile: 0311-84221431 Fax: 0311-84221431 Postcode: 051230

E-mail: zhmqx2020@163

Monitor

Organization name: Sinovac Biotech Co., Ltd.

Add: Peking University Biopolis, No. 39, Shangdi West Road, Haidian District, Beijing

Contact name: Hu Yuansheng

Mobile: 13436950182,010-82799318 Fax: 010-82890408 Postcode: 100085

E-mail: huys@sinovac.com

Organization for blood routine examination, blood biochemistry and routine urine test

Organization name: Hospital of Zanhuang County

Address: No. 50, East Huaiquan Road, Zanhuang Town, Zanhuang County

Contact person: Chen Huixian

Mobile: 13630828107 Fax: 84221431 Postcode: 051230

E-mail: jykzhxyy@163.com

Organization for Serum Antibody Detection

Organization name: National Institutes for Food and Drug Control

Add: No. 31, Huatuo Road, Daxing District, Beijing

Mobile: 010-53851770 Postcode: 102629

Organization for Data Management

Organization name: Meida Kelin (Nanjing) Medicine Technology Co., Ltd.

Address: Room A, 20F, Oriental International Technology and Science Building, No.58 Xiangcheng Road, Pudong District, Shanghai

Contact name: Sun Hualong

Mobile: 13816706496 Postcode: 200031 E-mail: hualong.sun@meta-clinical .com

Organization for Statistical Analysis

Organization name: Beijing KEY TECH Statistical Technology Co., Ltd.

Add: 1018-1119w, Sihui Building, Huihe South Street, Chaoyang District, Beijing

Contact name: Jiang Zhiwei

Mobile: 18618483152 Postcode: 100025 E-mail: zhi.wei.jiang@ktstat.com

Revision History of the Protocol

S/N	Original version No./version date/revision part	Current version No./version date/revision description
1	**When Version 2.6 is approved, Version 2.5 is invalid.**	
2	Version 2.5/ September 13,2021/	Version 2.6/February 18, 2022 /Add the detection of neutralizing antibody against Omicron strain at different time points before and after booster dose.
3	**When Version 2.5 is approved, Version 2.4 is invalid.**	
4	Version 2.4/ February 8, 2021/ Protocol home page	Version 2.5/ September 13,2021/ Protocol approver of sponsor is changed from " Qiang Gao " to "Xiaojuan Lian”
5	Version 2.4/ February 8, 2021/5 Preliminary Clinical Trial	Version 2.5/ September 13,2021/Update the results of preliminary clinical trial
6	Version 2.4/ February 8, 2021/6.5 Inoculation route and procedure	Version 2.5/ September 13,2021/ Add one booster dose at 10 months or 12 months after the second dose in subjects of Phase II trial
7	Version 2.4/ February 8, 2021/ 8.2.2 Endpoints of phase II trial	Version 2.5/ September 13,2021/ Increase endpoints of booster dose
8	Version 2.4/ February 8, 2021/8.3.2 Study Plan&10.1.2 Vistit Plan &10.6 Sampling	Version 2.5/ September 13,2021/ Increase the relevant content of booster immunization in Phase II trial. Meanwhile, the corresponding visit and sampling are modified in the visit plan and sampling content.
9	Version 2.4/ February 8, 2021/8.4.4 Unblinding regulations	Version 2.5/ September 13,2021/Increase"In order to protect the rights and interests of subjects in the placebo group and ensure that subjects can receive COVID-19 vaccine as soon as possible, subjects of appropriate age will receive COVID-19 vaccine (non-experimental vaccine) as required by the government during the trial. The low-dose and medium-dose groups remain blind until the database is locked."
10	Version 2.4/ February 8, 2021/10.7.6 Report on Serious Adverse Events	Version 2.5/ September 13,2021/Update reporting process of serious adverse event according to new GCP (2020 version)
11	Version 2.4/ February 8, 2021/10.11.1 Analysis Set	Version 2.5/ September 13,2021/ Increase related analysis set for booster immunization
12	Version 2.4/ February 8, 2021/	Version 2.5/ September 13,2021/ Increase the observation of adverse events of special interest and related study endpoints. adverse events of special interest include: Bell's palsy, sudden deafness/hearing loss, brachial plexus/multiple neuritis, Guillain-Barre syndrome, thrombocytopenic purpura, allergic purpura, thrombus, myelitis, myocarditis, immune thrombocytopenia, convulsion, multisystem inflammatory syndrome in children (MISC)
13	**When Version 2.4 is approved, Version 2.3 is invalid.**	
14	Version 2.3/ September 27, 2020/ 4.1.3 Toxicity test of repeated administration in rats	Version 2.4/ February 8, 2021/ Update the toxicity test of repeated administration in rats
15	Version 2.3/ September 27, 2020/ 4.1.5 Reproductive development toxicity test in rats	Version 2.4/ February 8, 2021/ Update the reproductive development toxicity test in rats
16	Version 2.3/ September 27, 2020/ 5. Preliminary Clinical Trial	Version 2.4/ February 8, 2021/ Update Phase I/II clinical trial results of COVID-19 Vaccine for the population aged 18-59 and aged 60 and above
17	Version 2.3/ September 27, 2020/ 6.2 Vaccine stability	Version 2.4/ February 8, 2021 /Update the study results of vaccine stability

18	Version 2.3/ September 27, 2020/ 8.2.2 Endpoints of phase II trial	Version 2.4/ February 8, 2021/ Add "seropositive rate and GMT of neutralizing antibody 3 months after the second dose" in the exploratory endpoint
19	Version 2.3/ September 27, 2020/ 8.3.2 Phase II research program & 10.1.2 Phase II subject visit plan & 10.6 Sampling	Version 2.4/ February 8, 2021/In the protocol Version 2.3, the blood samples will be collected from all subjects 6 months after the second dose. In the protocol Version 2.4, we revise that half of subjects will take blood at 3 months after the second dose and half of subjects will take blood at 6 months after the second dose.
20	Version 2.3/ September 27, 2020/ 10.11.1.2 Immunogenicity analysis set	Version 2.4/ February 8, 2021/ Add "immunization persistence analysis set"
21	Version 2.3/ September 27, 2020/ 10.7.6 Report on serious adverse events (SAE)	Version 2.4/ February 8, 2021/ Update 24h contact persons of the Sponsor's SAE report
22	Version 2.3/ September 27, 2020/ Pseudovirus antibody test	Version 2.4/ February 8, 2021/ When the versions 2.0-2.3 were revised, the pseudovirus neutralizing antibody test has not been finished in the Phase I/II clinical trial for the adults aged 18-59; SINOVAC added the pseudovirus neutralizing antibody test at different time points before and after the test vaccine immunity in the clinical trial protocols for the children and teenagers according to CDE's opinions. At present, SINOVAC has explored and researched the correlation between live virus and pseudovirus neutralizing antibody test methods in the Phase I/II clinical trial for adults. Besides, other manufacturers have not carried out the pseudovirus neutralizing antibody test at present. Hence, the related contents of pseudovirus neutralizing antibody detection were revised and deleted in this clinical trial protocol.
23	**When Version 2.3 is approved, Version 2.2 is invalid.**	
24	Version 2.2/ September 23, 2020/ List of Abbreviations of the Protocol	Version 2.3/ September 27, 2020/ The Sponsor's English name is modified as "Sinovac Life Sciences Co., Ltd."
25	Version 2.2/ September 23, 2020/ 8.3.1 Phase II research program	Version 2.3/ September 27, 2020/ Revise "Phase II clinical trials of this age group can be started after the safety observation is completed on Days 0-7 after the inoculation of the first dose of medium-dose group in all age groups in Phase I clinical trial and the safety is confirmed by DMC" to "Phase II clinical trials can be started after the safety observation is completed 0-7 days after the first dose of inoculation in all groups in Phase I clinical trial and the safety is confirmed by DMC".
26	Version 2.2/ September 23, 2020/ 10.1.1 Phase I subject visit plan	Version 2.3/ September 27, 2020/ Combination of visit 0 and visit 1
27	Version 2.2/ September 23, 2020/ 10.6 Sampling	Version 2.3/ September 27, 2020/ For the phase I clinical trial, revise the sampling time for antibody test before immunization from "sample after enrollment" to "sample together with the samples for blood routine examination and blood biochemistry during screening". See 10.6 Sampling for details.
28	Version 2.2/ September 23, 2020/ 10.6 Sampling	Version 2.3/ September 27, 2020/ Add "Samples for laboratory detection and the immunogenicity samples of the subjects not enrolled shall be disposed as medical waste at the research site after the work on that day"
29	**When Version 2.2 is approved, Version 2.1 is invalid.**	
30	Version 2.1/ November 10, 2020/ 8.3.1 Phase I research program	Version 2.2/ September 23, 2020/ Revise "According to the occurrence of solicited and non-solicited adverse events on

		Days 0-7 after the inoculation of first dose of the subject in the previous stage, as well as the abnormal occurrence of blood routine examination, blood biochemistry and routine urine test indexes, the next dose stage of inoculation can be entered only after the safety is confirmed by DMC" to "According to the occurrence of solicited and non-solicited adverse events on Days 0-7 after the first dose of inoculation in the previous stage, as well as the abnormal occurrence of blood routine examination, blood biochemistry and routine urine test indexes, the next stage of inoculation can be entered only after the safety is confirmed".
31	**When Version 2.1 is approved, Version 2.0 is invalid.**	
32	Version 2.0/ August 25, 2020/ Summary of the Clinical Protocol	Version 2.1/ September 10, 2020/ The "original China Food and Drug Administration (CFDA)" was deleted
33	Version 2.0/ August 25, 2020/ 4.1.2 Active systemic anaphylaxis test in guinea pigs	Version 2.1/ September 10, 2020/ The content of active anaphylaxis test was revised
34	Version 2.0/ August 25, 2020/ 5.1.1 Safety evaluation	Version 2.1/ September 10, 2020/ Revise the clinical trial safety evaluation result for adults
35	Version 2.0/ August 25, 2020/ 11.6 Management on clinical trial samples	Version 2.1/ September 10, 2020/ Revise from "The backup serum shall be kept or handled by the Sponsor" to "It shall be destroyed on site after confirmation by the Sponsor"
36	Version 2.0/ August 25, 2020/ 11.7 Preservation of clinical trial data	Version 2.1/ September 10, 2020/ Revise to "The data in the clinical trial must be kept in accordance with GCP requirements, and the Sponsor, responsible organization and trial site shall keep the clinical trial data for 5 years after the approval of test drug for marketing."
37	**When Version 2.0 is approved, Version 1.2 is invalid.**	
38	Version 1.2/ May 4, 2020/ 1 Introduction	Version 2.0/ August 25, 2020/ Update the introduction
39	Version 1.2/ May 4, 2020/ 2 Participating Organizations and their Responsibilities	Version 2.0/ August 25, 2020/ Change the site organization of the clinical trial; add the information on the organization for blood routine examination, blood biochemistry and routine urine test
40	Version 1.2/ May 4, 2020/ 3.4 Epidemiological characteristics	Version 2.0/ August 25, 2020/ Update the epidemic status of COVID-19 in China and in the world
41	Version 1.2/ May 4, 2020/ 3.5 Vaccine research and development	Version 2.0/ August 25, 2020/ Update the current situation of vaccine research and development
42	Version 1.2/ May 4, 2020/ 4.1.3 Toxicity test of repeated administration in rats	Version 2.0/ August 25, 2020/ Update the toxicity test of administration in rats
43	Version 1.2/ May 4, 2020/ 4.3 Challenge research	Version 2.0/ August 25, 2020/ Update the content of challenge research
44	Version 1.2/ May 4, 2020/ 5. Preliminary Clinical Trial	Version 2.0/ August 25, 2020/ Add the clinical trial results of COVID-19 Vaccine for adults
45	Version 1.2/ May 4, 2020/ 6.2 Vaccine stability	Version 2.0/ August 25, 2020/ Update the vaccine stability research results
46	Version 1.2/ May 4, 2020/ 6.4 Storage and transportation of vaccines	Version 2.0/ August 25, 2020/ Add "When the excessive temperature occurs temporarily for such reason as the signing after reception of vaccine, the opening/closing of door and other normal operations, is shall not be considered as deviation."
47	Version 1.2/ May 4, 2020/ 6.6 Information of test product	Version 2.0/ August 25, 2020/ Delete the information on high-dose vaccine; revise the lot No. and expiry date of medium-dose vaccine to 20200412 (lot No.) and April 8, 2023 (expiry date)

48	Version 1.2/ May 4, 2020/ 8.1.2<br>Sample size and power of test	Version 2.0/ August 25, 2020/ The sample size in Phase I is modified from 96 to 72; the sample size in Phase II is modified from 630 to 480
49	Version 1.2/ May 4, 2020 / 8.2.1<br>Endpoints of phase I trial	Version 2.0/ August 25, 2020/<br>**Secondary endpoint:** Add "the incidence of abnormalities in blood routine examination, blood biochemistry and routine urine test on Day 3 after each dose of test vaccine"<br>**Exploratory endpoint:**<br>Add "seroconversion rate, seropositive rate, GMT and GMI of pseudovirus neutralizing antibodies on Day 28 after the full-course immunization of test vaccine and on Day 28 after the first dose of immunization";<br>Revise the "seropositive rate and GMT of neutralizing antibody 12 months after full-course inoculation of test vaccine" to "positive rate and GMT of neutralizing antibody and pseudovirus neutralizing antibody 6 and 12 months after full-course inoculation of test vaccine"
50	Version 1.2/ May 4, 2020 / 8.2.2<br>Endpoints of phase II trial	Version 2.0/ August 25, 2020/<br>**Exploratory endpoint:**<br>Add "seroconversion rate, seropositive rate, GMT and GMI of pseudovirus neutralizing antibodies on Day 28 after the full-course immunization of test vaccine";<br>Revise the "seropositive rate and GMT of neutralizing antibody 12 months after full-course inoculation of test vaccine" to "positive rate and GMT of neutralizing antibody and pseudovirus neutralizing antibody 6 and 12 months after full-course inoculation of test vaccine"
51	Version 1.2/ May 4, 2020/ 8.3.1<br>Phase I research program	Version 2.0/ August 25, 2020/<br>Delete the high-dose group;<br>Add the test of laboratory test index for blood routine examination, blood biochemistry and routine urine test before each inoculation and on Day 3 after the inoculation;<br>Change of enrollment mode: The adolescents and children aged 3-17 is enrolled from low dose to high dose in the original protocol, which is revised to "enrolled in the group according to the principle of age group from being older to younger and from low dose to high dose" in this version. See 8.3.1 for details.
52	Version 1.2/ May 4, 2020/ 8.3.2<br>Phase II research program	Version 2.0/ August 25, 2020/<br>Delete the high-dose group;<br>Age distribution: revise "150 for subjects aged 3-6, 7-12 and 13-17 respectively" to "120, 180 and 180 for subjects aged 3-5, 6-11 and 12-17 respectively";<br>Enrollment mode: the enrollment of the age group in Phase II can be entered only after the completion of safety observation on Days 0-7 of all age groups in Phase I medium-dose stage and the safety confirmation.
53	Version 1.2/ May 4, 2020/ 8.4.4<br>Unblinding regulations	Version 2.0/ August 25, 2020/<br>Add "After the unblinding, the investigator responsible for observing and assessing the subjects and the clinical research associate responsible for source data validation shall remain blind until the database is locked."
54	Version 1.2/ May 4, 2020/ 8.4.5<br>Flowchart	Version 2.0/ August 25, 2020/<br>Update the flowchart of Phase I clinical trial
55	Version 1.2/ May 4, 2020/ 9.2<br>Exclusion criteria for subjects	Version 2.0/ August 25, 2020/<br>Delete "Wuhan and its surrounding areas" in Articles (1) and (3) of the exclusion criteria;

		Exclusion criteria: add<br>“(18) Subjects with abnormal laboratory test results such as in hematology and biochemistry which are beyond the range of reference values and of clinical significance in physical examination (applicable for Phase I clinical trial only):<br>1) Blood routine test: white blood cell count, hemoglobin and platelet count.<br>2) Blood biochemistry: alanine aminotransferase (ALT), aspartate aminotransferase (AST), total bilirubin (TBIL), creatinine (CR) and fasting blood-glucose;<br>3) Routine urine index: urine protein (PRO).”
56	Version 1.2/ May 4, 2020/ 10.1 Visit plan	Version 2.0/ August 25, 2020/<br>Revise the Phase I subject visit plan
57	Version 1.2/ May 4, 2020/ 10.6 Blood sample collection	Version 2.0/ August 25, 2020/<br>Change the blood sample for antibody detection from 2.5~3.5ml to 3.5~4.0ml;<br>Add blood sampling for blood routine examination and blood biochemistry: sample 2.0~2.5ml for blood routine examination every time; sample 3.0~3.5ml for blood biochemistry every time; add blood sampling for routine urine test: sample 5~10ml every time
58	Version 1.2/ May 4, 2020/ 10.6 Blood sample collection	Version 2.0/ August 25, 2020/<br>Revise “The serum for serum antibody detection shall be placed into 2 tubes, with tube A for neutralizing antibody detection and tube B for backup serum” to “Serum shall be separated from blood samples, by laboratory personnel, for serum antibody detection, and placed into 3 tubes, with tube A for neutralizing antibody detection, tube B for pseudovirus neutralizing antibody detection, and tube C for backup serum”.
59	Version 1.2/ May 4, 2020/ 10.7.1 Safety observation indexes	Version 2.0/ August 25, 2020/<br>Add the Phase I laboratory test indexes:<br>(1) Blood routine examination: white blood count, hemoglobin and platelet count; (2) Blood biochemistry: alanine aminotransferase (ALT), aspartate aminotransferase (AST), total bilirubin (TBIL), creatinine (CR) and blood glucose. 3) Routine urine: urine protein (PRO)
60	Version 1.2/ May 4, 2020/ 10.7.2 Definition of adverse event/reaction	Version 2.0/ August 25, 2020/<br>Update the definition of adverse event/reaction according to the new GCP
61	Version 1.2/ May 4, 2020/ 10.7.6 Report on serious adverse events	Version 2.0/ August 25, 2020/<br>(1) Add “The report object can be adjusted as required by current regulations, local regulatory authorities and the Independent Ethics Committee.”<br>(2) Delete the report procedure;<br>(3) Change the contact of Sinovac as: Liu Yongqing, phone: 13699256104
62	Version 1.2/ May 4, 2020/ 10.7.7 Safety evaluation criteria	Version 2.0/ August 25, 2020/<br>Add the grading standard of laboratory test indexes
63	Version 1.2/ May 4, 2020/ 10.9 Immunogenicity evaluation	Version 2.0/ August 25, 2020/<br>Add the evaluation standards for pseudovirus neutralizing antibody:<br>Positive criterion: if antibody titer≥1:30, it is positive.<br>Positive conversion: if the titer of pseudovirus neutralizing antibody is less than 1:30 before immunization and no less than 1:30 after immunization, it is considered as positive;

		or if the pseudovirus neutralizing antibody is no less than 1:30 before immunization and the titer of pseudovirus neutralizing antibody increases 4 times after immunization, the seroconversion of antibody is considered.
64	**When Version 1.2 is approved, Version 1.1 is invalid.**	
65	Version 1.1/April 29, 2020/1 Introduction	Version 1.2/May 04, 2020/The result of clinical trial for COVID-19 Vaccine for adults is updated.
66	Version 1.1/April 29, 2020/7.7 Protocol violation and deviation	Version 1.2/May 4, 2020/ "The protocol deviation/violation report shall be submitted to the Independent Ethics Committee for review and approval" is added.
67	Version 1.0/April 04, 2020/7.7 Protocol Violation and Deviation	Version 1.1/April 29, 2020/ "The protocol violations are listed as follows (including but not limited to): The subjects did not meet the inclusion criteria or met the exclusion criteria; the subjects received the wrong vaccine; the SAE was not reported within the specified time. The protocol deviations are listed as follows (including but not limited to): The test vaccine was given not within the window period specified in the protocol; blood sample was collected not within the window period specified in the protocol; interval between the test vaccine and other vaccines (except for rabies vaccine or tetanus vaccine in case of emergency) was not as prescribed in the protocol." The revision is made as follows: "Deviation from protocol: It refers to any change and non-compliance with the design or process in the protocol for clinical trial. The protocol deviation refers to the action of evaluating the safety or main indicators without affecting the rights and interests, safety and benefits of the subjects, or the integrity, accuracy and reliability of the test data. The severe protocol deviation (protocol violation) refers to the action of evaluating the safety or main indicators affecting the rights and interests, safety and benefits of the subjects, or the integrity, accuracy and reliability of the test data. For any protocol deviation/violation during the research, the on-site investigator shall report the fact, process, cause and impact to the responsible organization, and the Principal Investigator shall give opinions on the handling of such deviation/violation. The investigator shall provide targeted training on relevant links of such deviation/violation for the staff concerned to prevent the occurrence of similar incidents, and shall record the training process."
68	Version 1.0/April 04, 2020/8.2 Exclusion criteria for subjects	Version 1.1/ April 29, 2020/ Add "pregnant woman (including those with positive urine pregnancy test) or lactating women, and those who plan to get pregnant within 3 months"
69	Version 1.0/ April 4, 2020/ 8.3 Exclusion criteria for vaccination with the second dose	Version 1.1/ April 29, 2020/ Add "become pregnant after the last vaccination (including those with positive urine pregnancy test)"
70	Version 1.0/August 04, 2020/9.5 Safety follow-up observation	Version 1.1/April 29, 2020/The safety follow-up observation method is revised as follows: "The subjects shall be observed for any adverse events within 30 minutes after receiving each dose of vaccine, and Diary Cards and Contact Cards shall be used to collect any adverse events on days 0-7 and 8-28, respectively. The doctors explained the determination, measurement method, recording method, precautions and reporting method for adverse events. Systematic observation was conducted on day 7 after

		vaccination. Before day 7, the subjects were required to observe their own symptoms and signs and fill in the Diary Cards on a daily basis. The Investigator paid a face-to-face visit (or a visit by telephone) to all subjects, checked adverse events on days 0-7 after vaccination, collected the Diary Cards, and distributed the Contact Cards to record any adverse events on days 8-28 after vaccination on day 7 after vaccination, and paid a visit to all subjects, checked adverse events and collected the Contact Cards on day 28 after vaccination.
71	Version 1.0/ April 4, 2020/ 9.6 Blood sample collection	Version 1.1/ April 29, 2020/ Revise "about 3ml blood sample every time" to "about 2.5-3.5ml blood sample every time".
72	Version 1.0/April 04, 2020/9.7.4 Correlation between adverse events and vaccine	Version 1.1/April 29, 2020/The basis for determination of the correlation between adverse events and vaccine is revised as follows: "Definitely correlated: The evidence of receiving the test vaccine is presented; the chronological order of occurrence of adverse events and inoculation of test vaccine is reasonable; the occurrence of adverse events is more reasonably explained by the test vaccine than other reasons; positive result is obtained after the test vaccine is given repeatedly; the conditions of adverse events are consistent with the previous knowledge of this or this type of vaccine; Very probably correlated: The evidence of receiving the test vaccine is presented; the chronological order of occurrence of adverse events and inoculation of test vaccine is reasonable; the occurrence of adverse events is more reasonably explained by the test vaccine than other reasons; Probably correlated: The evidence of receiving the test vaccine is presented; the chronological order of occurrence of adverse events and inoculation of test vaccine is reasonable; the test vaccine and other reasons cannot be excluded from the causes of adverse events; Probably uncorrelated: The evidence of receiving the test vaccine is presented; adverse events are more likely to be caused by other reasons; negative or uncertain result is obtained after the test vaccine is given repeatedly; Definitely uncorrelated: The subject is not inoculate with the test vaccine; the chronological order of occurrence of adverse events and inoculation of test vaccine is not logical; or adverse events may be caused by other obvious reasons."
73	Version 1.0/April 04, 2020/	Version 1.1/ April 29, 2020/ Add Data Monitoring Committee (DMC) in "2 Participating Organizations and their Responsibilities"
74	Version 1.0/April 04, 2020/	Version 1.1/ April 29, 2020/The content related to day-0,14 immunization schedule is deleted.
75	Version 1.0/ April 04, 2020/3.5 Vaccine research and development	Version 1.1/ April 29, 2020/The vaccine research and development status is updated as follows: "Currently there is no approved treatment or vaccine for COVID-19 all over the world. According to the data released by WHO, as of April 26, 2020, a total of 82 candidate vaccines are subjected to the preclinical study, and 7 candidate vaccines are subjected to clinical trials, i.e., mRNA-1273 from Moderna, INO-4800 from Inovio, and mRNA vaccine from Pfizer, inactivated vaccine from Wuhan Institute of Biological Products, ChAdOx1-nCOV from Oxford

		University, Ad5-nCOV from CanSino Biologics Inc., and inactivated vaccine from SINOVAC."
76	Version 1.0/ April 4, 2020/ 7.3.1 Phase I research program	Version 1.1/ April 29, 2020/ The total sample size for Phase I clinical trial is modified from 192 to 96
77	Version 1.0/April 04, 2020/7.3.2 Phase II study plan	Version 1.1/ April 29, 2020/ The total sample size for Phase II clinical trial is modified from 960 to 630
78	Version 1.0/ April 4, 2020/ 9.7.5 Handling of adverse events	Version 1.1/April 29, 2020/During test observation, the subjects who develop fever with cough and other respiratory symptoms shall, when necessary, immediately go to the designated hospitals, have the throat swabs/sputum and anal swabs collected, and be subjected to imaging examinations such as CT to determine if the disease is caused by COVID-19 infection. In case of COVID-19 infection, it shall be treated according to SAE, and the occurrence of ADE shall be especially analyzed.

List of Abbreviations of the Protocol

**PROTOCOL TITLE**	A Randomized, Double-blind, Placebo-controlled Phase I/II Clinical Trial to Evaluate the Safety and Immunogenicity of COVID-19 Vaccine (Vero Cell), Inactivated in Healthy Children and Adolescents Aged 3-17 Years Old
**SPONSOR**	Sinovac Life Sciences Co., Ltd.
**PROJECT PHASE**	Phase I/II
**OBJECTIVE(S)**	To evaluate the safety and immunogenicity of SARS-CoV-2 vaccine
**EXPERIMENTAL DESIGN OF THE TRIAL**	A randomized, double-blinded, placebo-controlled, Phase I/II Clinical Trial
**PLANNED SAMPLE SIZE**	Total of 552 subjects，including 72 in the phase I and 480 in the phase II clinical trial
**SUBJECT SELECTION CRITERIA**	Healthy population aged from 3 to 17 years, balanced gender ratio.
**NAME AND FORMULATION OF DRUG**	SARS-CoV-2 Inactivated Vaccine<br>-Inactivated SARS-CoV-2 (300SU/0.5ml or 600SU/0.5ml)<br>-Aluminum hydroxide, disodium hydrogen phosphate, sodium dihydrogen phosphate, sodium chloride, etc.
**DOSAGE AND SCHEDULE**	Dosage: 0.5ml/dose<br>Primary Immunization Schedule: day 0,28<br>Booster Immunization: 10 months or 12 months after the second dose (only for phase II)
**ROUTE OF ADMINISTRATION**	Intramuscularly, deltoid region
**CHALLENGE SCHEDULE, if applicable**	None
**BLOOD SAMPLE COLLECTION**	**Phase I**<br>Day 0(-3), 3, 28,31 56,208, 388<br>**Phase II**<br>Day 0,56,118, 328,356,508,688 or 0,56,208,388, 416,568,748
**PARAMETERS OF SAFETY**	**Primary Endpoint**<br>－ Incidence rate of adverse reactions occurred from the beginning of the vaccination to 28 days after the second dose vaccination.<br>**Secondary Endpoints**<br>－ Incidence rate of adverse reactions within 7 days after each dose vaccination;<br>－ Incidence of abnormal laboratory index (blood routine test, blood chemistry test, and urine routine test) on the 3th day after each dose of vaccination in phase I;

	– Incidence rate of SAEs and AESIs from the beginning of the vaccination to 12 months after the last dose vaccination.
**PARAMETERS OF IMMUNOGENICITY**	**Primary Endpoint**<br>– The seroconversion rate of neutralizing antibodies 28 days after the second dose vaccination.<br>**Secondary Endpoints**<br>– The seropositive rate, GMT, and GMI of neutralizing antibodies 28 days after the second dose vaccination;<br>– The seroconversion rate, seropositive rate, GMT, and GMI 28 days after the first dose vaccination in phase I.<br>**Exploratory Endpoints**<br>– Phase Ⅰ: The seropositive rate and GMT of neutralizing antibody against live SARS-CoV-2 6 months and 12 months after the second dose vaccination.<br>– Phase Ⅱ: The seropositive rate and GMT of neutralizing antibody against live SARS-CoV-2 3 months, 6 months, 10 months and 12 months after the second dose vaccination.<br>– Phase Ⅱ: The seropositive rate, GMT, and GMI of neutralizing antibody against prototype strain (CZ strain) and Omicron strain 28 days after the booster dose;<br>– Phase Ⅱ: The seropositive rate, GMT of neutralizing antibody against prototype strain (CZ strain) and Omicron strain 6 months and 12 months after the booster dose.

List of Vocabulary Abbreviations

ADE	Antibody Dependent Enhancement
AE	Adverse Event
AESI	Adverse Events of Special Interest
CDC	Center for Disease Control and Prevention
CDE	Center for Drug Evaluation
CFDA	China Food and Drug Administration
COVID-19	Corona Virus Disease 2019
CFDI	Center for Food and Drug Inspection
CRF	Case Report Form
DMC	Data Monitoring Committee
eCRF	Electronic Case Report Form
ELISA	Enzyme-Linked Immune-sorbent Assay
EDC	Electronic Data Capture
FAS	Full Analysis Set
GCP	Good Clinical Practice
GMT	Geometric Mean Titer
GMI	Geometric Mean Increase
IEC	Independent Ethics Committee
ITT	Intention-to-Treat
MedDRA	Medical Dictionary for Regulatory Activities
NMPA	National Medical Products Administration
PI	Principal Investigator
PPS	Per Protocol Set
PT	Preferred Term
SAE	Serious Adverse Event
SARS	Severe Acute Respiratory Syndrome
SARS-CoV-2	Severe Acute Respiratory Syndrome Coronavirus 2
SOC	System Organ Class
SOP	Standard Operation Procedure
SS	Safety Set
SUSAR	Suspected Unexpected Serious Adverse Reaction

Summary of the Clinical Protocol

The COVID-19 Vaccine (Vero Cell), Inactivated developed by Sinovac Life Sciences Co., Ltd.,, Ltd. (hereinafter referred to as "SINOVAC") can induce active immunity and prevent diseases caused by the SARS-CoV-2. According to the preliminary immunogenicity studies, the vaccine can produce good neutralizing antibody responses, and has a good effectiveness in animals. At the same time, comprehensive safety evaluations were carried out on animals, showing that the new vaccine is safe. This protocol is drafted on the basis of *Regulation of Drug Registration*[1], *Good Clinical Practice (GCP)* [2-3], *Guidance on Vaccine Clinical Trial* [4], *Guidance on Vaccine Clinical Trial Quality Management (Trial)*[5] and *Guidance on SARS-CoV-2 Vaccine (Trail)* [6], etc.

The main objective is to evaluate the safety and immunogenicity of the test vaccine and the randomized, double-blinded and placebo-controlled experiment design is adopted. In Phase I, 72 adolescents and children aged 3-17 years (including 24 subjects aged 3-5 years, 6-11 years and 12-17 years respectively) will be selected. Sequential grouping method will be adopted according to the age from older to younger and the dose-escalating manner is used. The subjects in different dose stages of each age group will be randomly divided into 2 groups according to the ratio of 3: 1. Each enrolled subject will receive two doses of vaccine or placebo according to the immunization schedule of day 0, 28. Enrollment in Phase I clinical trial is divided into four stages as follows: the first stage: low-dose stage (12 subjects) for adolescents aged 12-17; the second stage: medium-dose stage for adolescents aged 12-17 (12 subjects) and low-dose stage for children aged 6-11 (12 subjects); the third stage: medium-dose stage (12 subjects) for children aged 6-11 and low-dose stage (12 subjects) for children aged 3-5; the fourth stage: medium-dose stage for children aged 3-5 (12 subjects). The next stage will start only with the condition that safety observation 0~7 days after the first dose of vaccination is finished, and the good safety profiles is confirmed according to the occurrence of the solicited and unsolicited adverse events, as well as the occurrence of the abnormal results of the blood routine, blood biochemical and urine routine testing.

The subjects for Phase II clinical trial can be enrolled only upon safety observation on Days 0-7 after the first dose of inoculation in all medium-dose groups for Phase I clinical trial and the safety confirmation by the Data Monitoring Committee (DMC). A total of 480 adolescents and children aged 3-17 years (including 120, 180, 180 subjects aged 3-5 years, 6-11 years and 12-17 years respectively) will be selected. The subjects of each age sub-group will be randomly divided into 3 groups according to the ratio of 2:2:1. All enrolled subjects will receive two doses of low-dose, medium-dose test vaccine and placebo respectively. Based on the results of immunogenicity 6 months after 2 doses from the Phase Ⅱ trial, DMC concluded that the available data support the introduction of booster immunization in children and adolescents. Therefore, the study plans to administrate one booster dose to subjects in the low -dose and medium-dose vaccine groups 10 or 12 months after the second dose.

The immediate reactions occur 30 minutes after each dose of vaccination will be observed on site.; the local and systemic solicited adverse events within 0~7 days and the non-solicited adverse events within 0~28 days will be collected; and the serious adverse events (SAE) and adverse events of special interest(AESI) monitoring from the beginning of vaccination to 12 months after the last dose will be collected to evaluate the safety of vaccines. In addition, the blood and urine of all volunteers in Phase I will be collected at different time points before and after immunization for blood routine examination, blood biochemistry and routine urine tests to evaluate the safety of the vaccine.

In Phase I, venous blood will be collected at different times before and after immunization to test the neutralizing antibody for evaluating the immunogenicity and immune persistence of vaccine. In Phase II, venous blood will be collected at different time points before and after immunization for neutralizing antibody detection to evaluate the immunogenicity, immune persistence and effect of booster immunization of the vaccine. In addition, the neutralizing antibody against Omicron strain will be detected before and after booster dose to explore the cross neutralization effect.

The clinical protocol will be independently undertaken by the investigator after being approved by independent ethics committee (IEC). The clinical research associates designated by the sponsor will monitor the whole process of the study to ensure the safety of the trial.

Contents

1 Introduction ... 18
2 Participating Organizations and their Responsibilities ... 18
2.1 Sponsor ... 18
2.2 Organization Responsible for the Clinical Trial ... 19
2.3 Study Site Institution ... 20
2.4 Sample Testing Institute 1 ... 21
2.5 Sample Testing Institute 2 ... 22
2.6 Monitor Institution ... 22
2.7 Data Management Institution ... 23
2.8 Statistical Analysis Institution ... 24
2.9 Data Monitoring Committee (DMC) ... 24
3 Background and Principle ... 25
3.1 Summary ... 25
3.2 Virology ... 25
3.3 Clinical Manifestation ... 26
3.4 Epidemiological Characteristics ... 26
3.5 Vaccine Research and Development ... 27
4 Preclinical Study and Laboratory Evaluation of Vaccine ... 27
4.1 Safety Study ... 27
4.2 Immunogenicity Study ... 32
4.3 Study of Virus Challenge ... 34
4.4 Cross Neutralizing Research ... 37
5 Preliminary Clinical Trial ... 38
5.1 Phase I/II clinical trial of COVID-19 vaccine in adults ... 38
5.2 Phase I/II clinical trial of COVID-19 vaccine in elderly ... 41
5.3 Phase I/II clinical trial of COVID-19 vaccine in children and adolescents ... 42
6 Product Features ... 44
6.1 Preparation Technology and Formulation of the Vaccine ... 44
6.2 Stability ... 45
6.3 Control Vaccine ... 45
6.4 Transportation and Storage of Vaccine ... 45
6.5 Inoculation route and procedure ... 45
6.6 Information of Investigational Vaccine ... 46
6.7 Vaccine Packaging ... 46
7 Objective ... 46
7.1 Phase I clinical trial ... 46
7.2 Phase II clinical trial ... 46
8 Study Design ... 47
8.1 Design ... 47
8.2 Endpoint ... 47
8.3 Study Plan ... 48
8.4 Randomization and blinding ... 50

8.5 Flow chart 52
8.6 Study Duration 54
8.7 Trial Suspension and Early Termination 54
8.8 Protocol Violation and Deviation 54
8.9 Pregnancy event 54
9 Subjects 55
9.1 Inclusion Criteria for Subjects 55
9.2 Exclusion Criteria for Subjects 55
9.3 Exclusion Criteria for Vaccination with Subsequent Dose 56
9.4 Withdrawal and Termination Criteria for Subjects 56
10 Method and Schedule 57
10.1 Visit Plan 57
10.2 Recruitment and Informed Consent 63
10.3 Screening and Random Enrollment 63
10.4 Vaccination 63
10.5 Safety Follow-up and Observation 63
10.6 Sampling 64
10.7 Safety Evaluation 65
10.8 Concomitant Medication and Vaccination 71
10.9 Immunogenicity evaluation 72
10.10 Data management 72
10.11 Statistical analysis 73
11 Clinical Trial Monitoring 76
11.1 Sponsor’s Responsibility 76
11.2 Investigator’s Responsibility 77
11.3 Personnel Training 77
11.4 Compliance Guaranteeing of Subjects 77
11.5 Management of Test Vaccine 77
11.6 Sample Management in Clinical Trials 78
11.7 Storage of Data on Clinical Test 78
11.8 Ending Criteria for Clinical Trial 78
12 Ethical Approval 78
12.1 Review and Approval 78
12.2 Field Supervision 78
12.3 Confidentiality 79
13 Modification of Clinical Trial Protocol 80
14 Disclosure and Publication of Data 80
15 References 80

1 Introduction

The COVID-19 Vaccine (Vero Cell), Inactivated (hereinafter referred to as "COVID-19 Vaccine") developed by Sinovac Life Sciences Co., Ltd.(hereinafter referred to as "SINOVAC") can induce the body to produce active immunity and prevent the diseases caused by SARS-CoV-2. Preliminary immunogenicity studies showed the SAR-Cov-2 vaccine can induce good neutralizing antibody responses, and have a good effectiveness in animals. Comprehensive safety evaluations showed the SARS-CoV-2 vaccine was safety in animals. This clinical trial is designed to evaluate the safety and immunogenicity of the investigational vaccine.

The Phase I/II clinical trial for COVID-19 Vaccine in adults aged 18-59 was launched in April 2020 in Suining County, Jiangsu Province. At present, the immunogenicity evaluation and safety observation of all subjects on Day 28 after two doses of vaccination have been completed. The results showed that COVID-19 Vaccine produced by SINOVAC had good safety and immunogenicity in adults. Both emergency vaccination schedule and routine vaccination schedule can induce body to produce antibody quickly. The Phase I/II clinical trial for COVID-19 Vaccine in elderly people aged 60 and above was launched in May 2020 in Renqiu County, Hebei Province. In June 2020, 72 subjects have been enrolled for Phase I and 350 subjects for Phase II. The current result showed that the COVID-19 vaccine is safe and meets the requirements for vaccine clinical trial in healthy population aged 3~17 years. To evaluate the safety and immunogenicity of the COVID-19 vaccine in healthy population aged from 3 to 17 years, this clinical trial protocol is designed.

2 Participating Organizations and their Responsibilities

2.1 Sponsor

2.1.1 Responsibilities

The Sponsor of the Clinical Trial is Sinovac Life Sciences Co., Ltd., the main responsibilities of which are as follows:

- Providing the preliminary clinical trial protocol and approving the final protocol with signature and seal.
- Providing approval documents for the Clinical Research, the Investigator's Brochure for the Clinical Trial (preclinical safety information of the product), executive standards for products and other site application documents.
- Providing vaccines for the study and issuing the acceptable verification report.
- Evaluating and selecting the responsible organization and site of the Clinical Trial, assigning the Monitor to perform the assessment and accreditation of the site of the Clinical Trial and the monitoring duties according to the GCP requirements, and being ultimately responsible for the quality of the Clinical Trial.
- Participating in the investigation and handling of the cases with adverse reaction to vaccine, and providing medical treatment and relevant compensation for the cases with clinically proven adverse event related to the vaccination according to relevant regulations. For other cases, please refer to the Working Agreement.
- Providing funds for the Clinical Research.

2.1.2 Profile

Sinovac Life Sciences Co., Ltd., as the former R&D Center of Sinovac Biotech Co., Ltd. and a biological high-tech enterprise solely-invested and established by Sinovac Biotech (Hong Kong) Limited, was incorporated in 2009, with the registered capital of USD 9.60 million. The Company is a Zhongguancun high-tech enterprise and Zhongguancun gold seed enterprise.

SINOVAC is specialized in the research, development and technical services of vaccines for human use and related products to provide technical support for the prevention and control of serious infectious diseases. Relying on the Group's advantages in vaccine research and development and industrialization over the years, the Company has gradually formed a research and development mode with enterprises as the main body of research and development and the combination of the efforts of enterprises, universities and research institutions, and built the virus isolation identification technology platform, cell factory platform, microcarrier fermentation technology platform, virus purification technology platform, bacterial fermentation and purification platform, polysaccharide-protein combination technology platform, freeze-drying technology platform, animal evaluation platform, quality control platform and diagnostic reagent raw materials development platform, the expertise of which complements each other's advantages with cross penetration to promote the stable and efficient progress of Company's research and development.

SINOVAC has undertaken 2 special projects of national major new drug development and one science and technology program in Beijing, and obtained 12 authorized patents for invention in China. The clinical research of the 23-valent pneumopolysaccharide vaccine developed by the Company has been successfully completed and its industrialization has been realized in Sinovac. The Company is developing DPT polio Hib series combined vaccine, 13-valent pneumococcus conjugate vaccine, recombinant hepatitis B vaccine and other varieties.

2.2 Organization Responsible for the Clinical Trial

2.2.1 Responsibilities

The organization responsible for the Clinical Trial is Hebei Provincial Center for Disease Control and Prevention, and its main responsibilities are as follows:

- Participating in the formulation of tables and cards required by the Vaccine Clinical Trial Protocol and the Trial;
- Participating in the drafting of informed consent, the preparation of SOP for site operation of the Clinical Trial, and the application for approval by the Independent Ethics Committee; organizing the selection and evaluation of the site for the Clinical Trial that meets the requirements of GCP, and filing with the "Record Management Information Platform for Drug Clinical Trial Institutions" of the National Medical Products Administration;
- Organizing the implementation of the Clinical Trial, and performing quality control over its implementation process;
- Instructing the site, reporting Serious Adverse Events occurring during the Clinical Trial to the provincial medical products administration, Sponsor and Independent Ethics Committee in a timely manner, and carrying out investigation and handling;
- Participating in database locking and keeping a backup of the locked database for verification;

- Reporting the implementation progress of the Clinical Trial to relevant administrative departments and writing the summary report of the Clinical Trial.

2.2.2 Profile

Hebei Provincial Center for Disease Control and Prevention is a public institution directly affiliated to the Health Commission of Hebei Province, which was formally established in August 2001 based on the dissolution or merger of 6 organizations, namely, the former Provincial Health and Epidemic Prevention Station, Provincial Endemic Disease Prevention and Control Institute, Provincial Occupational Disease Prevention and Control Institute, Provincial Institute of Radiological Health, Provincial Academy of Medical Sciences, and Provincial Tuberculosis Prevention and Control Center, and it is also known as the Health Inspection Center of Hebei Province, Academy of Medical Sciences of Hebei Province, Occupational Disease Prevention and Treatment Institute of Hebei Province, and National Food Safety Risk Monitoring Hebei Center. It is the technical guidance center of Hebei Province for disease prevention and control.

The center has 40 offices (institutes, teams, centers), including 14 administrative and business management offices and 26 business offices. The checked staff size is 558, and currently there are 472 active staff, including: 144 with a master degree or above, accounting for 30.5% of the total number of active staff, 159 with the title of a senior professional post, accounting for 44.28% of the total number of active staff, 22 provincial management experts, provincial middle-aged and young experts, and staff enjoying special government allowance, and 4 provincial-level model workers.

The center has two provincial-level key medical subject units, namely, the cardiovascular and cerebrovascular disease prevention and treatment organization and food safety risk monitoring laboratory. It is the teaching base for preventive medicine of 4 colleges and universities in and outside Hebei Province, namely, Hebei Medical University, Hebei University, North China University of Science and Technology, and Shanxi Medical University, and it undertakes the teaching of about 30-50 students every year. It has accumulatively undertaken more than 250 scientific research projects of various types, including 11 major national science and technology projects, 30 national collaboration projects, and 2 international cooperation projects, and gained more than 110 scientific research awards at the ministerial level or above, including 25 awards for scientific and technological advancement at ministerial and provincial-level.

Hebei Provincial Center for Disease Control and Prevention began to carry out vaccine clinical trials in accordance with GCP in 2008, and up to now, it has undertaken 28 clinical trial projects (with 19 completed) and established a relatively complete vaccine clinical trial organization, management and quality control system. After the Guidelines for Quality Management in Clinical Trial of Vaccines (Trial) is implemented, the center made adjustments according to relevant requirements, and established a vaccine clinical trial institution on November 15, 2013, and a vaccine clinical research institute on November 25, 2013, i.e., department of professional management of vaccine clinical trials, to be responsible for the organization, management and implementation of vaccine clinical trials. The vaccine clinical research institute has 8 professionals, 2 of whom are chief physicians and 7 hold a master degree respectively. All personnel have attended the training on GCP organized by the National Medical Products Administration (NMPA).

2.3 Study Site Institution

2.3.1 Responsibilities

The site of the Clinical Research is Zanhuang County and the study site institution is the Center for Disease Control and Prevention of Zanhuang County, the main responsibilities of which are as follows:

- Cooperating in the assessment and filing of the test site;
- Organizing personnel with corresponding qualification to participate in the research work, and all participants to read and understand the content of the research protocol in detail and strictly observe the protocol to ensure that there is sufficient time to complete the clinical research within the time limit as specified;
- Organizing the on-site implementation, including the organization and selection of subjects, obtaining the Informed Consent signed by subjects, screening and enrollment, vaccination, safety visit, sample collection, serum separation, sample cryopreservation and submission;
- Data input and ensuring that all collected data are true, accurate, complete and legal;
- Accepting the monitoring and inspection by the Monitor or Inspector dispatched by the Sponsor, and the inspection and visit by the medical products administration to ensure the quality of the Clinical Research;
- Ensuring that subjects are properly handled when they suffer adverse reactions/events during the research, and in case of serious adverse reactions/events, handling and reporting such conditions immediately according to relevant operating procedures;
- Keeping relevant clinical trial data during the Clinical Trial.

2.3.2 Profile

The Disease Control and Prevention of Zanhuang County, established in December 2006, covers an area of 2,500m2 and floor area of 2,350m2. At present, it has 40 employees, including 6 with bachelor degree, 29 with junior college degree and 5 with technical secondary school degree; 36 professional medical workers, including 2 with associate senior title, 10 with intermediate title and 24 with primary title; 13 medical practitioners, 18 nurses and 5 laboratorians. It is mainly responsible for the monitoring and control of infectious diseases, immunization planning, handling of health emergencies, epidemic prevention of major disasters, inspection, testing and hygienic evaluation, health education and health promotion, as well as prevention and control of chronic non-infectious diseases.

The Disease Control and Prevention of Zanhuang County is equipped with two vaccine cold storages and one cold-train transport vehicle. Digital management has been realized in vaccination clinic and cold chain transportation, with many large instruments and equipment such as gas chromatograph, ion chromatograph, atomic absorption spectrometer, atomic fluorescence spectrometer, PCR instrument, automatic nucleic acid extraction workstation, microplate reader, spectrophotometer and biosafety cabinet. With physics and chemistry lab, microbiology lab, PCR biological lab, HIV screening lab, serum lab, iodine deficiency disorder lab, P2 lab, it has 179 test items passing the qualification accreditation, proving the strong inspection and testing ability.

2.4 Sample Testing Institute 1

2.4.1 Responsibilities

Zanhuang Hospital, its responsibilities are:

- Performing blood routine examination, blood biochemistry and routine urine test.

2.4.2 Profile

The Zanhuang Hospital is a comprehensive Grade 2A hospital integrating medical treatment, teaching, scientific research, first aid and rehabilitation. It covers an area of 20,000m2 and has fixed assets of RMB 170 million. At present, it employs 799 staff members, including 586 with professional skill, 18 with senior title, 74 with associate senior title, 187 with intermediate title; in addition, it has 600 beds in service. In 2019, the Hospital records 229,243 outpatients, 22,811 in-patients, and 3,498 surgical cases, with ward utilization rate of up to 120.1%. It is equipped with nearly 100 pieces of large and medium-sized medical equipment, such as color ultrasound instrument, MRI, CT, DR, CR, electronic gastroscope, thoracoscope, peritoneoscope, surgical microscope and automatic biochemical analyser, and advanced laminar flow operating room to accommodate 9 million-grade operating rooms, which can support various independent major operations like radical operations for lung cancer, digestive tract cancer and cervical cancer, as well as common craniotomy in neurosurgery department, orthopedic operation, artificial hip and knee replacement. The Hospital has 35 clinical and medical science departments, in which the internal medicine department is divided into cardiovascular, neurology, respiratory, digestive and other professional sections; the surgery department is divided into general surgery, bone surgery, cerebral surgery, urology department, proctology department and other professional sections; the pediatric department has high-standard neonatal ward with high level in the treatment of neonatal and infant diseases; the ENT department carries out cataract, phacoemulsification, intraocular lens implantation and other operations. The function of the Hospital is becoming better and better.

2.5 Sample Testing Institute 2

2.5.1 Responsibilities

National Institutes for Food and Drug Control, the main responsibilities of which are as follows:

- Detecting the serum neutralizing antibody.

2.5.2 Profile

National Institutes for Food and Drug Control is a public institution directly under the National Medical Products Administration, the national statutory body and the supreme technical arbitration body for testing the quality of medicines and biological products, and the “WHO Collaborating Center for Drug Quality Assurance” designated by the World Health Organization. In accordance with the laws, it implements the approval and registration inspection, import inspection, supervision inspection and safety assessment of medicines, biological products, medical devices, food, health food, cosmetics, experimental animals, packing materials and other products in various fields, as well as lot release of biological products, is responsible for the research, distribution and management of culture and virus seed used for the reference material and production verification of national drugs and medical devices, and carries out related technical research.

2.6 Monitor Institution

2.6.1 Responsibilities

The Clinical Research Department of Sinovac Biotech Co., Ltd. is responsible for the monitoring of the Clinical Trial.

- Conducting monitoring of the Clinical Trial according to GCP, protocol and SOP;
- Assisting the Sponsor in undertaking the screening and training of the institutions for the Clinical Trial, holding kick-off meeting and other work;
- Verifying the test process and progress;
- Verifying the signing of informed consent;
- Verifying the qualifications of the investigators and the effectiveness of the implementation equipment;
- Verifying the transportation, storage, distribution, use, return and disposal of the vaccines for the Clinical Trial;
- Verifying the collection, storage and transportation of biological samples;
- Verifying the handling of adverse events;
- Verifying the logicality of the original records and the report documents in the Trial;
- Completing the monitoring after the Trial, etc.

2.6.2 Profile

Since its establishment in 2002, Sinovac Clinical Research Department has independently undertaken the organization, implementation, monitoring, data and statistical analysis management of many clinical trials, including inactivated hepatitis A vaccine, hepatitis A and B combined vaccine, SARS vaccine, H1N1 vaccine, H5N1 vaccine, EV71 vaccine, 23-valent pneumococcus vaccine, varicella vaccine, inactivated poliomyelitis vaccine, and quadrivalent influenza vaccine, making it experienced in clinical trial organization, implementation and management.

2.7 Data Management Institution

2.7.1 Responsibilities

Meida Kelin (Nanjing) Medicine Technology Co., Ltd. is responsible for clinical trial data management.

- Formulating the Data Management Plan and Data Validation Plan according to protocol requirements;
- Providing EDC and other related online services;
- Carrying out data management in accordance with the Technical Guidelines for Clinical Trial Data Management during the Trial, and confirming that all data reports and records are correct and complete;
- Conducting data cleaning, raising questions about research data, and assisting investigators in verification and clarification;
- Preparing the data management report.

2.7.2 Profile

Meida Kelin (Nanjing) Medicine Technology Co., Ltd. established in September 2014, is a Contract Research Organization (CRO) mainly engaged in the outsourcing of data related services in clinical trials for domestic and foreign pharmaceutical companies. It has now offices in Shanghai, Beijing, Xi’an and Shenyang, and is in a strategic partnership with the CRO Clinical Service Center providing all-round services. It has provided data management, statistical analysis and drug safety alert services for phases I~IV and bioequivalence clinical trials of the innovative drugs and generic drugs of dozens

of domestic and foreign pharmaceutical companies. It has:

- Standard Operating Procedure (SOP) and strict quality management system that meet the requirements of ICH-GCP/FDA 21 CFR part 11/international or domestic clinical trials;
- Personnel familiar with the clinical trial design, implementation, data management and statistical analysis experience in China, US, EU, Japan, South Korea and other countries, and with relevant drug management regulations and implementation rules;
- Complete education and training system.

2.8 Statistical Analysis Institution

2.8.1 Responsibilities

Beijing KEY TECH Statistical Technology Co., Ltd. is responsible for the statistical analysis of the Clinical Trial.

- Writing the randomization, sample size and statistical analysis parts of the Clinical Trial Protocol;
- Writing the statistical analysis plan according to Clinical Trial Protocol;
- Conducting the randomization and blinding of the Clinical Trial;
- Carrying out the statistical analysis according to the proposed statistical analysis plan and writing the statistical analysis report.

2.8.2 Profile

Beijing KEY TECH Statistical Technology Co., Ltd. (referred to as “KEY TECH”), incorporated in August 2017 in Beijing, is a wholly domestic-funded company engaged in data management and statistical analysis services of clinical trial. It focuses on the biostatistics service of clinical research, and mainly provides, with respect to registered clinical trials, the statistical strategy consultation for drug research plan throughout the whole process, the statistical design and statistical analysis, etc. KEY TECH has currently set up offices in Beijing, Xi’an and Nanning, now with 43 employees, who have mainly graduated from the Fourth Military Medical University, Peking University, Sichuan University and other first-class universities in China. At present, there are 21 statisticians/statistical programmers, 18 data managers, 1 quality control person and 3 non-business personnel among the employees. In terms of education background, there are 3 doctors, 6 masters and 34 bachelors.

Since its establishment, KEY TECH has assisted the Sponsor in obtaining 8 approval documents for clinical trial and completed 12 new drug applications, including 3 Class I new drugs of biological products, the first 13-valent pneumonia vaccine in China and the second Adalimumab monoclonal antibody product in China. Among such applications, 6 vaccine clinical research projects have audited by CFDI and 4 have been subjected to the technical review by CDE recently. KEY TECH signed an agreement with Abbott in 2019 for statistical consulting services in the Asia Pacific Region, establishing long-term partnership with leading innovative pharmaceutical companies at home and abroad.

2.9 Data Monitoring Committee (DMC)

The Data Monitoring Committee consists of specialists in clinical medicine, epidemiology and statistics. Its main responsibilities are as follows:

- Performing safety data review and clinical trial risk assessment to ensure that the Trial is carried out in a safe and standard way.

3 Background and Principle

3.1 Summary

Since December 8, 2019, several cases of pneumonia for unknown cause were reported in Hubei, with most of the patients working or living in South China Seafood Wholesale Market where live animals are sold. At early stage, this pneumonia presented severe symptoms of acute respiratory infection, with some patients rapidly developing to acute respiratory distress syndrome (ARDS). This pneumonia, which was later proved to be human-to-human transmission, escalated rapidly in early January 2020, and there were cases found in provinces of China and more than 20 other countries, including Japan, Singapore and US. Chinese Center for Disease Control and Prevention (CDC) identified a novel coronavirus from a patient's throat swab sample on January 7, 2020. The World Health Organization (WHO) declared the pneumonia outbreak caused by the novel coronavirus to be a public health emergency of international concern (PHEIC) on January 31, 2020. WHO declared the outbreak to enter the international pandemic phase on March 12, 2020.

As shown by research, the novel coronavirus gene sequences were most closely associated with two SARS-like coronaviruses from bat (bat-SL-CoVZC45 and bat-SL-CoVZXC21)[7] International Committee on Taxonomy of Viruses (ICTV) declared the official class name of this novel coronavirus as Severe Acute Respiratory Syndrome Coronavirus 2 (SARS-CoV-2) on February 12, 2020, while WHO declared the official name of the disease caused by the virus as COVID-19 on the same day.

3.2 Virology

Coronavirus (COV) is an important pathogen of human and vertebrate that can infect the respiratory tract, gastrointestinal tract, liver and central nervous system of humans, livestock, birds, bats, mice and many other wild animals. Since the outbreak of Severe Acute Respiratory Syndrome (SARS) in 2003 and Middle East Respiratory Syndrome (MERS) in 2012, the possibility for COVs to transmit from animals to humans has been proved. COVs belongs to the Coronavirinae subfamily of the Nidovirales coronavirus family, which includes four genera: α-coronaviruses, β-coronaviruses, γ-coronaviruses, and δ-coronaviruses[7-8].

SARS-CoV-2 is from genus β that is enveloped with round or oval and often pleomorphic particles in the diameter of 60-140nm, and is a plus-stranded RNA virus. Its gene characteristics are clearly different from those of SARSr-COV and MERSr-COV. The present research shows that it has more than 85% homology with bat SARS-like coronavirus (bat-SL-COVZC45). COVID-19 could be found in respiratory epithelial cells within about 96h when isolated and cultured in vitro, while within about 6 days when isolated and cultured in Vero E6 and Hun-7 cell lines.

So far, the full-length genome sequences among virus samples are almost identical, suggesting that no significant virus variation has taken place. Close monitoring of SARS-CoV-2 also shows that no significant variation has been found in viruses isolated from the environment, previously isolated from humans and recently isolated[9]. However, there is still the possibility of future mutation or recombination in which the virulence may increase or decrease.

The understanding of physicochemical properties of coronavirus mainly comes from the researches of

SARS-COV and MERS-COV. The virus is sensitive to ultraviolet and heat. At 56°C for 30 minutes, diethyl ether, 75% ethanol, chlorine-containing disinfectant, peracetic acid, chloroform and other lipid solvents can effectively inactivate the virus, but chlorhexidine cannot[10].

3.3 Clinical Manifestation

According to the current epidemiological survey, the incubation period is 1-14 days, mostly 3-7 days, with fever, fatigue and dry cough as the main manifestations. A few patients have symptoms such as nasal congestion, runny nose, sore throat, myalgia and diarrhea. Critical patients often develop dyspnea and/or hypoxemia one week after attack, with rapid development to acute respiratory distress syndrome, septic shock, incorrigible metabolic acidosis, haemorrhagic and coagulation dysfunction in severe cases. It is worth noting that the course of the disease in the severe and critical patients may present moderate to low fever, and even no obvious fever.

Some cases of children and newborns show atypical symptoms, such as vomiting, diarrhea and other digestive tract symptoms or only mental weakness and polypnea. Mild patients only show low fever, fatigue, etc., without pneumonia.

The prognosis of most patients is favorable and a few patients are in critical condition. The elderly and those with chronic underlying diseases have a poor prognosis. The clinical course of pregnant and birth-giving women with COVID-19 is similar to that of patients of the same age, and children have relatively mild symptoms[9].

3.4 Epidemiological Characteristics

Transmission routes and susceptible groups

The main source of infection of COVID-19 is the patients infected by SARS-CoV-2, and asymptomatic carriers may also become the source of infection. The main routes of transmission are respiratory droplets and contact transmission, the virus is transmitted through the droplets produced during patients' cough, sneeze and talk, susceptible people inhaling them will be infected, and the population is generally susceptible. Exposure to high concentration of aerosol in a relatively closed environment for a long time presents the possibility of aerosol transmission.

The fecal-oral transmission route remains to be determined. Recently, SARS-CoV-2 was detected in the feces of the confirmed patients in Wuhan, Shenzhen and even the US in the first case, indicating that the virus can replicate and exist in the digestive tract and suggesting that fecal-oral transmission is possible[11], but it has not been established that eating food contaminated by the virus will cause infection and transmission. There is also a view that the virus in feces may be retransmitted by aerosol formed by droplets containing the virus, and further investigation is needed.

It has been currently reported that there is the case where the mother is a confirmed COVID-19 patient and the newborn is with positive viral nucleic acid by throat swab 30h after birth, suggesting that SARS-CoV-2 may cause neonatal infection through mother-to-child transmission[12].

Epidemic Status of COVID-19 in China

As of 11:00 on August 16, 2020 (CEST), there have been 89,761 confirmed cases of COVID-19 in China, with 4,710 deaths[13]. As pointed out in the Joint Investigation Report of China-WHO on Novel Coronavirus Pneumonia (COVID-19)[14], among the 55,924 laboratory confirmed cases reported, the median of age is 51 years old, the age range is 2 days~100 years old, and the interquartile range is

39~63 years old. Most of the cases (77.8%) are in the range of 30~69 years old. Among them, males account for 51.1%, cases from Hubei for 77% and peasants or manual workers for 21.6%.

In China, human-to-human transmission of COVID-19 mainly occurs within families. Detailed information on continuous transmission among family members in some provinces can be obtained from cluster case surveys and some family transmission case studies. There are 1,836 cases in total in Guangdong and Sichuan, and the reported 344 cluster cases involve 1,308 cases, of which 78%~85% occur in family members. Research on the transmission within family members is ongoing, but preliminary findings in Guangdong estimate that the second-generation secondary attack rate in family members is about 3%~10%. As the pandemic continues, community cluster infections are also increasing with hospital cluster attack, although family cluster infections are dominant[144].

Epidemic Status of COVID-19 in the World

As of 11:00 on August 17, 2020 (CEST), there have been 21,260,760 cumulative confirmed cases of COVID-19 in the world, with 761,018 deaths. The countries with high incidence of COVID-19 are, in turn, the US (5,258,565 cumulative confirmed cases), Brazil (3,275,520 cumulative confirmed cases), India (2,589,682 cumulative confirmed cases), Russia (917,884 cumulative confirmed cases), South Africa (583,653 cumulative confirmed cases), Peru (516,296 cumulative confirmed cases), Mexico (511,369 cumulative confirmed cases), Columbia (445,111 cumulative confirmed cases), Chile (383,902 cumulative confirmed cases), Spain (342,813 cumulative confirmed cases), Iran (338,825 cumulative confirmed cases) and the United Kingdom (316,371 cumulative confirmed cases). The outbreak has spread to over 200 countries around the world, leading to the global COVID-19 pandemic.[13]

3.5 Vaccine Research and Development

At present, there are no approved specific therapies or vaccines against COVID-19. According to the data published by WHO, there were 167 candidate vaccines under development, 138 candidate vaccines in preclinical studies, and 29 candidate vaccines in clinical trials, including 7 candidate vaccine in Phase IIII clinical trials (i.e., inactivated COVID-19 vaccines from SINOVAC, Beijing Institute of Biological Products and Wuhan Institute of Biological Products, ChAdOx1-S from the University of Oxford, and mRNA vaccine from Moderna, mRNA vaccine from BioNTech, and protein subunit vaccine from Novavax), 11 candidate vaccines in Phase I/II clinical trials, and 11 candidate vaccines in Phase I clinical trials as of August 13, 2020.

4 Preclinical Study and Laboratory Evaluation of Vaccine

4.1 Safety Study

The single dose toxicity study in rats, active systemic anaphylaxis test in guinea pigs, toxicity test of repeated administration in rats, toxicity test of repeated administration in macaca fascicularis and reproductive development toxicity test in rats have been carried out for the test vaccine, the results of which are as follows:

4.1.1 Single Dose Toxicity Study on Rats

Objective: To evaluate the acute toxicity of SARS-CoV-2 Vaccine on Sprague-Dawley (SD) rats within 14 days after a single dose, so as to provide toxic data for acute poisoning.

Design: according to the body weight of animals measured before administration, 20 animals with similar body weight and quarantine inspection passed were selected for the test, with half males and half females, which were randomly divided into 2 groups by sex section, namely the test group and the negative control group. The rats in the test group were intramuscularly injected with the proposed clinical high-dose vaccine by 0.5ml/1200SU/rat (SARS-CoV-2 Unite) and the rats in the negative control group were intramuscularly injected with normal saline by 0.5ml/rat, which were observed for 14 days after the administration and further observed by gross anatomy.

Results: No death or near-death was observed in the animals of both the test group and the negative control group, and no abnormalities were observed in clinical observation. The body weights of the animals in all groups normally increased, and no statistical differences were observed in the body weights of animals in the test group when compared with the animals in the negative control group of same sex during the same period, and no significant effects of drug administration on animal intake were observed. Visual observations of pathological gross autopsy showed that no abnormities were seen in major organs and tissues of the animals in all the groups.

Conclusion: No abnormalities associated with administration were seen in SD rats inoculated with high-dose vaccines in clinic, and the maximum tolerated dose (MTD) in SD rats was greater than or equal to 1,200 SU/1 dose/rat.

4.1.2 Active systemic anaphylaxis test in guinea pigs

Objective: to observe the immediate systemic anaphylaxis in guinea pigs sensitized by intramuscular injection of COVID-19 Vaccine (once every other day, for 3 times in total) and stimulated by intravenous injection on D19 and/or D26, so as to provide animal experimental data for clinical research of the test article.

Design: according to the body weight of animals measured before administration, animals with similar body weight were selected, and 36 Hartley guinea pigs were randomly divided into 4 groups, i.e., low-dose test group, high-dose test group, negative control group and positive control group, respectively sensitized by intramuscular administration on D1, D3 and D5 and stimulated by intravenous administration on D19 (14 days after the last sensitization) and D26 with 0.5ml/1200SU/dose test article, normal saline and human hemoglobin. The first 3 animals in each group were stimulated by intravenous injection in the feet, and the stimulation dose in each group was twice the sensitization dose. Clinical observation was performed after administration. The experimental design is shown in the table below:

Table 1 Experimental Design of Active Systemic Anaphylaxis in Guinea Pigs

Group	Test article/control	Qty. of animals	Sensitization (i.m) D1,D3,D5		Stimulation (i.v) D19 and D26	
			Dosage of administration	Capacity of administration (mL/Nr.)	Dosage of administration	Capacity of administration (mL/Nr.)
1	Negative control	9	0	0.5	0	1
2	Positive control	9	20 mg/Nr.	0.5	40 mg/Nr.	1
3	Low-dose test article	9	0.1 dose/Nr.	0.05	0.2 dose/Nr.	0.1
4	High-dose test article	9	1 dose/Nr.	0.5	2 doses/Nr.	1

Results: no abnormal reaction was observed in general clinical observation. The body weight of animals in each group was in normal growth according to the weighing before grouping, before the last sensitization and before administration on the day of stimulation respectively. The low-dose group, high-dose group and negative control group all showed negative anaphylaxis. The positive control group was stimulated on D19 and D26 and showed positive anaphylaxis.

Conclusion: No allergic reaction was found in guinea pigs inoculated with high-dose vaccines in clinic.

4.1.3 Repeated Dose Toxicity Test in Rats

Objective: to evaluate the possible toxicity and determine target organs for toxicity and recovery of toxic reaction in SD rats 4 weeks after repeated intramuscular injection of COVID-19 Vaccine, determine the safe dose for repeated administration, and provide basic data for clinical trial and application of the test articles.

Design of the 3-dose schedule: according to the body weight of animals measured before grouping, 150 animals with similar body weight and satisfactory quarantine inspection results were selected, which were randomized into 7 groups by sex for the main test groups (1~4 groups, i.e. low-dose test group, high-dose test group, negative control group and adjuvant control group) and satellite groups (5~7 groups, i.e. low-dose test group, high-dose test group and negative control group). There were 15 animals per sex in each main test group and 5 animals per sex in each satellite group. The rats in the low-dose test group, high-dose test group, negative control group and adjuvant control group were administrated by intramuscular injection of 0.5ml/300SU/dose of test article, 0.5ml/1,200SU/dose of test article, 0.5ml/dose of normal saline and 0.5ml/dose of adjuvant respectively on D1, D8 and D15, with recovery period of 2 weeks.

Design of the 4-dose schedule: 80 SD rats (half males and half females) aged 5-6 weeks were selected and randomly divided into 2 experimental groups according to body weight: negative control group (CN group) and COVID-19 Vaccine group (T group), with 40 animals in each group, including 30 animals in the main test group and 10 animals in the satellite group. One dose was administered intramuscularly at 1,200 SU/0.5 ml/rat/dose at weeks 0, 1, 2 and 3 respectively, with recovery period of 2 weeks.

Results of the 3-dose schedule: During the test, no death or near death was observed. Clinical observations showed no abnormalities and no abnormal changes in body weight, body temperature and eye examination resulted from drug administration. No abnormality related to the drug administration was observed in the coagulation function, blood biochemistry and urinalysis, T lymphocyte subsets, and cytokines of animals in each group. Compared with the same-sex negative control group in the same phase, the basophils of the female animals in the low-dose test group increased 3 days after the first administration (D4), and the neutrophils of male animals in the low-dose test group increased and eosinophils in female animals increased, and neutrophils in male animals in the high-dose test group increased 3 days after the last administration. Based on the mechanism and mode of action of the test article, it is considered that the above changes may be correlated to the immune response and/or local irritation caused by the administration of the test article. In addition, compared with the same-sex negative control group in the same phase, the lymphocytes of male animals in the adjuvant control group and low-dose test group decreased 3 days after the last administration (D18), and the count of reticulocytes of female animals in the adjuvant control group

apparently decreased, and the monocytes of female animals in the low-dose test group apparently increased at the end of the recovery phase (D29). As the above changes are not obvious and only found in single sex, and there is no correlation with the administered dose, it is considered to be of no toxicological significance.

Pathological examination showed no toxicologically significant regular change in the organ weight and organ coefficient of the animals in each group, and gross observation showed no obvious abnormal change. Microscopic observation showed that 3 days after administration (D18), granulomatous inflammation was visible locally in 17/20, 13/20 and 10/20 animals in the adjuvant control and low/high-dose test groups respectively, with mild to moderate lesions; this change was considered a local reaction caused by accumulation of aluminum adjuvant, which was an expected reaction caused by intramuscular injection of a vaccine containing aluminum adjuvant. At the end of the 2-week recovery period (D29), granulomatous inflammation was still visible locally in 7/10, 6/10 and 6/10 animals in the adjuvant control and low/high-dose test groups respectively, suggesting that the local irritant response caused by drug administration had not recovered.

Results of the 4-dose schedule: During the test, no death or near death was observed. Clinical observations showed no abnormalities and no abnormal changes in body weight, body temperature and eye examination resulted from drug administration. At the end of the last administration and at the end of the recovery period, no abnormality related to the drug administration was observed in the coagulation function, blood biochemistry and urinalysis, T lymphocyte subsets, and cytokines of animals in each group.

Pathological examination showed no toxicologically significant regular change in the organ weight and organ coefficient of the animals in each group after the last administration and recovery period, and gross observation showed no obvious abnormal change. During the whole experiment, no visible local reactions, including hyperemia, edema, induration and necrosis, were observed at the injection sites of rats in administration group. According to the microscopic observation, after the last administration, 12 cases (12/20) showed muscle interstitial inflammatory cell infiltration at the injection sites, including one with fibroblast hyperplasia. At the end of the recovery period, 3 (3/10) cases showed muscle interstitial inflammatory cell infiltration at the injection sites.

Conclusions: The COVID-19 Vaccine was given to SD rats by repeated intramuscular injections once a week for 3 or 4 consecutive times. During administration and at the end of the 2-week recovery period, no systemic toxic reactions were observed in animals at doses of 300 SU/rat and 1,200 SU/rat. It was determined that no observed adverse effect level (NOAEL) was 1,200SU/rat. Irritation related to aluminum adjuvant was seen at some local injection sites, some irritation was partially reversible 2 weeks after the withdrawal, and no immunotoxic reactions were observed.

4.1.4 Repeated Dose Toxicity Test on Cynomolgus Monkey

Objective: to evaluate the possible toxicity and target organ in macaca fascicularis after 4 weeks of repeated intramuscular injection of COVID-19 Vaccine, as well as the recovery of toxicity after 4 weeks of withdrawal, so as to provide animal experimental data for clinical research of the test article.

Design: according to the body weight of animals measured before grouping, 40 animals were randomly divided into 4 groups by sex section, i.e. low-dose test group, high-dose test group, negative control group and adjuvant control group, with 10 macaca fascicularis in each group, half males and half

females, which were administrated by intramuscular injection of 0.5ml/300SU/dose of test article, 0.5ml/1200SU/dose of test article, 0.5ml/dose of normal saline and 0.5ml/dose of adjuvant respectively on D1, D8 and D15, and safety observation was performed until 14 days after the last administration for euthanasia anatomy. Test indexes include clinical observation such as anaphylaxis and local injection reaction, body weight/body temperature/electrocardiogram/blood pressure/ophthalmic testing, cliniopathological markers (blood counts, coagulation function, blood biochemistry and urine analysis), immunological indexes (T lymphocyte subsets, cytokines, C-reaction protein, alexin and antibodies) and pathological examination (gross anatomical observation and histopathological examination).

Result: During the test, no death or near death was observed in the animals of each group. Clinical observations showed no abnormalities related to drug administration and no abnormal changes in body weight, body temperature, electrocardiogram, blood pressure or eye examination resulted from drug administration. No abnormal clinicopathological indexes or immunological indexes associated with drug administration were observed. Pathological examination showed that, on Day 3 after the last administration (Day 18), granulomatous inflammation or single cell infiltration was visible locally in 5/6, 6/6 and 5/6 animals in the adjuvant control and low/high-dose groups respectively, with mild to moderate lesions; this change was considered a local reaction caused by accumulation of aluminum adjuvant, which was an expected reaction caused by intramuscular injection of a vaccine containing aluminum adjuvant. At the end of the 2-week recovery period, macrophage granulomatous inflammation was still visible locally in 3/4, 4/4 and 4/4 of the animals in the adjuvant control group and low/high-dose groups respectively, suggesting that the local irritant response of drug administration had not recovered.

Conclusions: The COVID-19 vaccine was given to machin by repeated intramuscular injections once a week for consecutive 2 weeks, totaling to 3 doses. During administration and at the end of the 2-week recovery period, no systemic toxic reactions were observed in animals at doses of 300 SU/rat and 1,200 SU/rat. It was determined that no observed adverse effect level (NOAEL) was 1,200SU/rat. Local irritation related to aluminum adjuvant was seen at some local injection sites, and no immunotoxic reactions were observed.

4.1.5 Reproductive and development toxicity test in rats

Objective: evaluate the effects of repeated intramuscular injection of COVID-19 Vaccine in SD rats before mating to duration of pregnancy until lactation period on the fertility of male and female rats, and on the development of pregnant/lactation female rats, fetus and fetal rats, understand the effect of the vaccine on the development of teratogenic fetal rats and offspring rats, and study the antibody level in the blood of fetus or offspring rats, so as to provide reference for the safe administration to special population in the Clinical Trial.

Design: according to the body weight of animals measured before administration, the animals were randomly divided into 4 groups by sex section, i.e. low-dose test group, high-dose test group, negative control group and adjuvant control group, with 28 males and 56 females in each group, which were administrated with 0.5ml/300SU/dose of test article, 0.5ml/1200SU/dose of test article, 0.5ml/dose of normal saline and 0.5ml/dose of adjuvant respectively. The male rats were administrated for 4 times before mating, on D1, D8, D15 and D28 respectively. Female rats were administrated for 3 times before mating, on D1, D8 and D15 respectively. Female and male rats started mating in a cage one

week after the end of the administration to male rats, and female rats were administrated once respectively on gestational day 6 (GD6) and on postnatal day 7 of offspring rats (PND7). Male rats were euthanized 3 weeks (Day 57) after the mating season, 1/2 pregnant rats (28 rats/group) were euthanized at GD20, and 1/2 pregnant rats (28/group) were euthanized upon the postpartum lactation period (PND21). Based on the clinical observation in the experiment, the weight, appetite and reproductive capacity of parental male and female rats were inspected; the cesarean delivery of GD20 necropsied male rats, development of embryo-fetal rats, appearance of fetal rats, bone and viscera were inspected; the sperm viability, counting and morphology of parental male rats were inspected, and the histopathology of main reproductive organs of parental male and female rats were inspected; the survival, weight, body and reflective development indexes of the postpartum F1 neonatal rats were inspected; and the anti-novel coronavirus specificity IgG antibody and its neutralizing antibody in the serum of GD20 necropsied male rats and fetal rats as well as parturient female rats and F1 neonatal rats were determined.

Results: Test results show that the COVID-19 Vaccine was repeatedly injected into SD rats by means of intramuscular injection at doses of 300 SU/rat and 1,200 SU/rat from the time before copulation to the embryonic implantation and parturition. Results showed no effects on the fertility of male and female rats as well as growth and development of F1 neonatal rats, no obvious adverse reaction on the pregnant/lactant female rats, and no developmental toxicity and teratogenicity of embryo-fetal rats. Besides, the anti-novel coronavirus specificity IgG antibody and its neutralizing antibody were inspected in the serum of GD20 necropsied male rats, fetal rats, parturient female rats and F1 neonatal rats in the high and low dose groups of samples for test, with a detectable rate of 100%.

4.2 Immunogenicity Study

To evaluate the immunogenicity of COVID-19 vaccine, mice and rats were immunized intraperitoneally and intramuscularly with vaccine of different dosage, and different adsorption methods at different immunization schedules. Blood samples were collected at different time points for the testing of serum neutralizing antibody titer and IgG antibody titer after immunization. The formulation, dosage and immune schedule of the vaccine are determined according to the immunogenicity results.

Study Design:

- **Determination of aluminum adsorption and non-aluminum adsorption processes for the vaccine**

Two different processes were employed to prepare aluminium-containing SARS-CoV-2 vaccines of 1200 SU/0.5 ml, 600 SU/0.5 ml, 300 SU/0.5 ml and 150 SU/0.5 ml, and aluminium-free SARS-CoV-2 vaccines of 1200 SU/0.5 ml, 600 SU/0.5 ml and 300 SU/0.5 ml. Mice were intraperitoneally immunized by the above vaccines, 10 mice per group, 0.5 ml per mouse. For the mice immunized with one injection, serum was collected on Day 7, Day 14 and Day 21 after immunization; for the mice immunized with two injections on Day 0, 7 and Day 0, 14, serum was collected on Day 14, Day 21 and Day 28, and serum IgG antibody titer was determined separately. Negative animal control was set. Immunogenicity of vaccines prepared by two different processes was compared via a comparison of the antibody titers, the specific study design is shown in the table below:

Table 2 Study design of the comparison between immunogenicity of aluminium-adsorbed and non-aluminium Adsorption COVID-19 Vaccine

Dosage (SU/0.5ml)	COVID-19 Vaccine, Inactivated				Non-aluminum adsorption COVID-19 Vaccine, Inactivated			
	Batch No.	One dose	Two doses (D0, 7)	Two doses (D0, 14)	Batch No.	One dose	Two doses (D0, 7)	Two doses (D0, 14)
1200SU	20200303-1	10	10	10	20200303-5	10	10	10
600 SU	20200303-2	10	10	10	20200303-6	10	10	10
300SU	20200303-3	10	10	10	20200303-7	10	10	10
150 SU	20200303-4	10	10	10	/	/	/	/

- **Determination of immunization dosage and schedules of COVID-19 Vaccine**

Mouse groups: 4 dose groups with antigen content of 300SU/0.5ml, 600SU/0.5ml, 1200SU/0.5ml and 2400SU/0.5ml (corresponding lot no. as 20200213-1~4) were for intraperitoneal immunization of mice as per 10 mice/group and 0.5ml/mouse by emergency and routine immunization schedules.

Rat test groups: 4 dose groups with antigen content of 300SU/0.5ml, 600SU/0.5ml, 1200SU/0.5ml and 2400SU/0.5ml (corresponding lot no. as 20200213-1~4) were for intramuscular immunization of rats as per 5 rats/group and 0.5ml/rat by emergency and routine immunization schedules. Vaccine diluent was also provided as negative control. The immunization and blood sampling are shown in Table 3.

The proposed dosage of COVID-19 Vaccine for the Clinical Trial was determined by analyzing the immunization dosage, neutralizing antibody titer and enzyme labelled antibody titer. At the same time, the immunization effects of one dose, two doses and three doses were compared to determine the immunization schedule.

Table 3 Study Design for Immunization Dosage and Schedules of COVID-19 Vaccine

Immunization Schedule	Immunization Schedule	Date of blood sampling	Amount
Emergency schedule	Day 0	Day 7, 14, 21, 28, 35, 42	10 Mice, 5 Rat
	Day 0, Day7	Day 14, 21, 28, 35, 42	10 Mice,5 Rat
	Day 0, Day 3, Day 7	Day 7, 14, 21, 28, 35, 42	10 Mice,5 Rat
Routine Schedule	Day 0, Day 14	Day 21, 28, 35, 42	10 Mice,5 Rat
	Day 0, Day 14, Day 28	Day 35, 42	10 Mice,5 Rat

Study results:

- **Determination of aluminum adsorption and non-aluminum adsorption processes for the vaccine**

After intraperitoneal immunization of mice by one dose of COVID-19 Vaccine with and without aluminum adjuvant, a certain level of anti-SARS-CoV-2 enzyme labelled antibody could be produced in the mice on D7 after primary immunization. The antibody level of 1200SU/0.5ml vaccine without aluminum adjuvant was comparable to that of 300SU/0.5ml vaccine with aluminum adjuvant. The immunogenicity of the vaccine with aluminum adjuvant was obviously better than that of the vaccine without aluminum adjuvant.

- **Determination of immunization dosage and schedules of COVID-19 Vaccine**

(1) For the same immunization schedule, different dose groups were for immunization of the same

species of animals, and the neutralizing antibody titer was detected at the same blood collection point. There was a good dose-effect relationship between the immunization dosage and the neutralizing antibody titer produced.

(2) For the same dose groups, different immunization schedules (one dose, two doses and three doses) were used for immunization of the same species of animals, and the enzyme labelled antibody titer was detected at the same blood collection point. The immunization effect using the schedules of two and three doses on mice was lower than that using the schedule of one dose. The immunization effect using the schedules of two and three doses on rats was higher than that using the schedule of one dose. The immunization effect using the schedules of two and three doses was equivalent due to the short interval among three doses.

(3) For the same dose groups and the immunization schedule of two doses (day 0,7 and day 0,14) at different time points, the enzyme labelled antibody level of the day 0,14 immunization schedule was one order of magnitude higher than that of the day 0,7 immunization schedule on D21, indicating that the interval of more than 14 days is required between two doses in the Clinical Trial.

(4) For different dose groups and the same immunization schedule of two doses, the neutralizing antibody levels under 1200SU and 2400SU were basically the same.

Research conclusion: the formulation of aluminum adjuvant was selected, the dosages determined to be used for the Clinical Research were 300SU/dose, 600SU/dose and 1200SU/dose, and the immunization schedule of two doses was selected.

4.3 Study of Virus Challenge

Objective: to evaluate the animal protective effect of COVID-19 Vaccine under challenge of SARS-CoV-2 after immunizing animals with COVID-19 Vaccine according to different immunization schedules and dosages, and evaluate the existence of Antibody Dependent Enhancement (ADE), so as to provide animal experimental data for clinical research and application.

Design: Rhesus monkeys were immunized by COVID-19 Vaccine according to different immunization schedules and dosages, novel coronavirus seed was used to attack the animals on Day 21~42 after the first immunization, the protective effect of the vaccine was evaluated according to the results of observation of clinical symptoms, serum antibody detection and histopathological examination of the rhesus monkeys, and the existence of ADE under different antibody levels was observed, with the research design shown in the table below:

Table 4 Virus challenge study design

Group	Vaccination Schedule（Days）	Dosage	Day of attack after the first dose	Day of euthanasia after the attack	Number of animals
Vaccine group -3 doses	0,7,14	High dosage (1200SU/0.5ml)	23	7	4
		Medium dosage (600SU/0.5ml)	22	7	4
Adjuvant group-3 doses	0,7,14	/	21	7	2
Model (saline) group	/	/	21	7	2
Vaccine group-2 doses	0,14	High dosage (1200SU/0.5ml)	23	7	4

Group	Vaccination Schedule（Days）	Dosage	Day of attack after the first dose	Day of euthanasia after the attack	Number of animals
		Medium dosage (600SU/0.5ml)	22	7	4

Two-dose test results:

No significant rise in body temperature was observed in the animals of the model group after challenge. The body temperature of two animals in the medium-dose group exceeded 40°C, and no abnormalities were observed in the high-dose group in body temperature. High levels of virus were detected in throat swabs, anal swabs and lung tissue for the model group. Compared with the model group, the medium-dose group was tested negative for throat swab virus on Day 3, Day 5 and Day 7 after challenge (7 days after challenge), 4 tested negative for lung tissue virus on Day 7 after challenge, and all 4 showed mild interstitial pneumonia, suggesting that the medium-dose vaccine had a significant protective effect; compared with the model group, 3 in the high-dose group were tested negative for throat swab virus on Day 3, Day 5 and Day 7 after challenge, 4 tested negative for lung tissue virus on Day 7 after challenge, and all 4 showed mild interstitial pneumonia, suggesting that the high-dose vaccine had a significant protective effect.

The changes of antibody levels in each group of rhesus monkeys are shown in Table 5. Based on the results of immune protection in the medium-dose group and high-dose group as well as the level of neutralizing antibody before challenge, it is suggested that neutralizing antibody titers greater than or equal to 1:48 after 2 doses of immunization had significant protective effect.

Table 5 Changes in Antibody Levels of Rhesus Monkeys in Each Group after COVID-19 Vaccination

	Animal No.	Day 0 after immunization	Day 7 after immunization	Day 14 after immunization	Day 21 after immunization	Day 3 after challenge	Day 5 after challenge	Day 7 after challenge
Medium-dose group	K21	<8	<8	4	64	64	48	256
	K22	<8	<8	4	128	48	64	128
	K23	<8	<8	6	48	32	96	1024
	K24	<8	<8	32	64	256	128	1024
GMT	/	/	/	7.4	70.8	70.8	78.4	430.5
High-dose group	K17	<8	<8	16	128	1024	512	512
	K18	<8	<8	16	256	256	512	512
	K19	<8	<8	4	96	512	1024	512
	K20	<8	<8	<4	64	192	256	1024
GMT	/	/	/	6.7	119.1	400.7	512.0	608.9
Adjuvant group	K9	<8	<8	<4	<4	<4	4	8
	K10	<8	<8	<4	<4	<4	<4	<8
GMT	/	/	/	/	/	/	/	/
Model group	K15	<8	<8	<4	<4	<4	6	12
	K16	<8	<8	<4	<4	<4	8	8
GMT	/	/	/	/	/	/	6.9	9.8

Three-dose test results:

No significant rise in body temperature was observed in the animals of the model group after challenge, and no abnormalities were observed in the adjuvant, medium-dose and high-dose groups in body temperature. White blood cell count decreased and LYMPH% increased after all groups of animals

were infected, and there was no significant difference between the medium/high-dose groups and the model group. Blood biochemical tests on all the groups of animals on D0 and D14 after immunization and when the animals were put to death showed that all the indexes were within the normal range. High levels of virus were detected in pharyngeal swabs, anal swabs and lung tissue for the model group. In the medium-dose group, the average level of virus in pharyngeal and anal swabs decreased on Day 7 after challenge compared with the model group; in the high-dose group, pharyngeal and anal swabs were tested negative for virus on Day 7 after challenge; 3 in the medium-dose group were tested negative for virus in lung tissue on Day 7 after challenge, and all 4 in the high-dose group were tested negative for virus in lung tissue on Day 7 after challenge.

Both the model and adjuvant groups were negative for neutralizing antibodies on Day 21 after immunization. In the medium-dose group, the neutralizing antibody GMT was 1:61.3 and reached 1:400.7 on Day 7 after challenge. In the high-dose group, the neutralizing antibody GMT was 1:50.1 and reached 1:145 on Day 7 after challenge, as shown in Table 6.

Table 6 Changes in Antibody Levels of Rhesus Monkeys in Each Group after COVID-19 Vaccination

	Animal No.	Day 0 after immunization	Day 7 after immunization	Day 14 after immunization	Day 21 after immunization	Day 3 after challenge	Day 5 after challenge	Day 7 after challenge
**Medium-dose group**	K5	<8	<8	6	64	32	384	1024
	K6	<8	<8	4	24	32	64	512
	K7	<8	<8	48	384	128	512	768
	K8	<8	<8	6	24	32	64	64
**GMT**		/	/	9.1	61.3	45.3	168.5	400.7
**High-dose group**	K1	<8	<8	12	48	24	96	256
	K2	<8	<8	16	64	96	512	384
	K3	<8	<8	6	32	24	48	96
	K4	<8	<8	6	64	16	48	48
**GMT**		/	/	9.1	50.1	30.7	103.2	145.9
**Adjuvant group**	K9	<8	<8	<4	<4	<4	4	8
	K10	<8	<8	<4	<4	<4	<4	<8
**GMT**		/	/	/	/	/	/	/
**Model group**	K15	<8	<8	<4	<4	<4	6	12
	K16	<8	<8	<4	<4	<4	8	8
**GMT**		/	/	/	/	/	6.9	9.8

Pathological findings of some animals are detailed in Fig. 1-6.

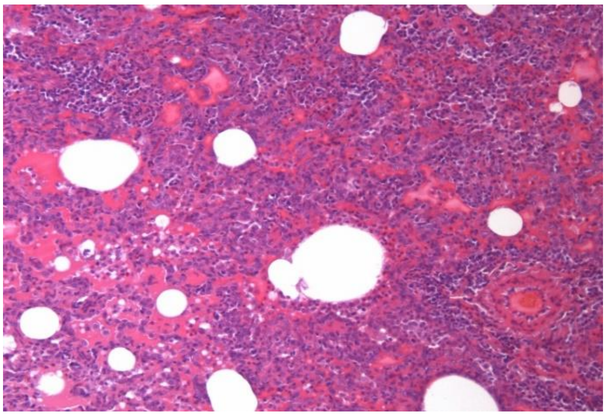

Fig. 1 Model Group K15 Minor Lobe of Right Lung Severe Interstitial Pneumonia H.E.×100

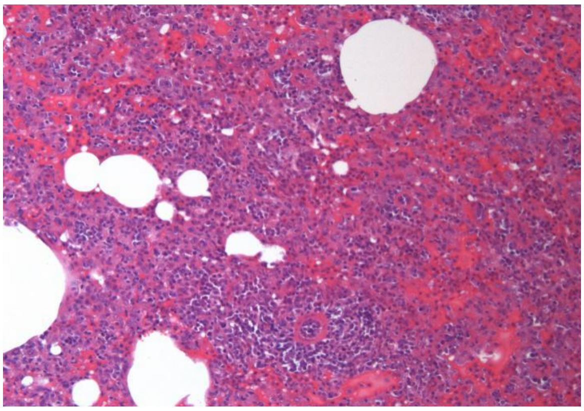

Fig. 2 Adjuvant Group K10 Middle Lobe of Right Lung Severe Interstitial Pneumonia H.E.×100

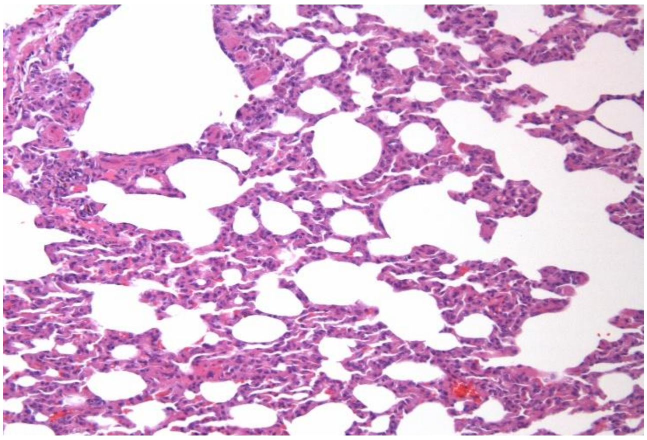

Fig. 3 Medium-dose Group K21 Superior Lobe of Right Lung Mild Interstitial Pneumonia H.E.×100

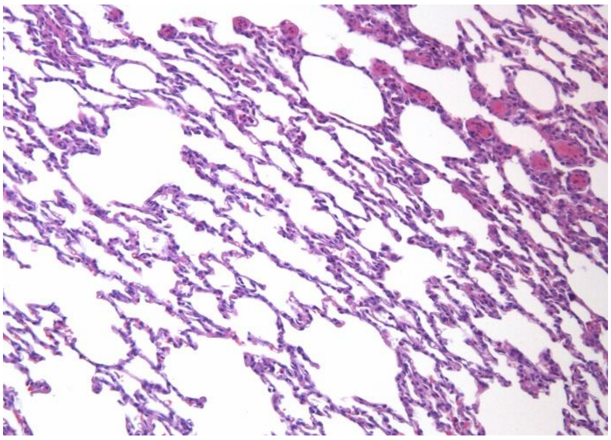

Fig. 4 Medium-dose Group K22 Inferior Lobe of Left Lung No Abnormality Seen H.E.×100

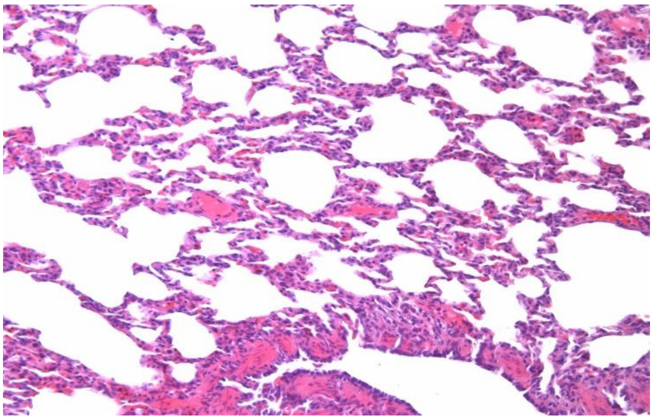

Fig. 5 High-dose Group K17 Inferior Lobe of Right Lung Mild Interstitial Pneumonia H.E.×100

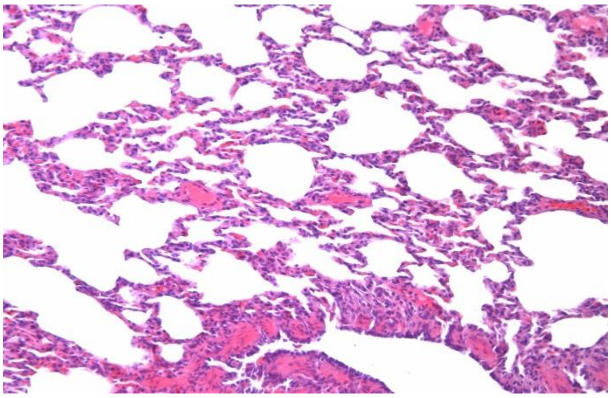

Fig. 6 High-dose Group K19 Inferior Lobe of Left Lung No Abnormality Seen H.E.×100

Conclusions: The COVID-19 vaccine had apparently protective effects, and no ADE phenomenon was observed.

4.4 Cross Neutralizing Research

Objective: To evaluate the cross-neutralizing effect of SARS-CoV-2 immunized serum against different virus strains.

Design: Select novel coronavirus strains separated at different time points and sites to conduct the cross neutralization test with the anti-novel coronavirus serums from different sources (including convalescent serum from COVID-19 patients and anti-novel coronavirus (inactivated CZ02 strain) animal immune serum). The novel coronavirus strains selected are shown in the following below:

Table 7 Basic Information of Strains

Strain	Lot No.	Source
CW01	CW01-W-202002-01	Wuhan (China)
WXY-C3	WXY-202002-01	Zhejiang (China)
CZ01	CZ01-W-202002-01	Zhejiang (China)
CB01	CB01-P5-202002-001	Wuhan Institute of Virology (China)
V34	V34-P4(D3)	Chinese Academy of Military Sciences (China)
CZ12	CZF-202003-01	Zhejiang (China)

Strain	Lot No.	Source
CZ30	WGF-202003-01	Zhejiang (China)
HAC	HAC-202003-01	Imported from Italy
HJL	HJL-202003-01	Imported from Italy
QHF	QHF-202003-01	Imported from Spain
SSH	SSH-202003-01	Imported from Switzerland
ZYF	ZYF-202003-01	Imported from Italy

Result: All convalescent serums of patients infected with novel coronavirus from different sources cross-neutralized with both domestic and imported novel coronavirus strains, with basically consistent results. The serum obtained by inoculation of inactivated COVID-19 vaccine that was prepared from CZ02 candidate strains cross-reacted well with strains separated domestically and overseas, showing consistent reaction trend with the serum from clinically confirmed COVID-19 patients.

Conclusion: The immune serum of novel coronavirus from different sources can produce cross-neutralizing reaction to novel coronavirus in different situations, and the results of cross-neutralizing reaction is basically consistent.

5 Preliminary Clinical Trial

5.1 Phase I/II clinical trial of COVID-19 vaccine in adults

5.1.1 Safety Evaluation

➢ **Adverse Reaction**

For Phase I/II clinical trial for the adults aged 18-59 years old, a total of 372 subjects received the COVID-19 vaccine/Placebo according to the emergency immunization schedule (Day 0,14). From the beginning vaccination to 28 days after the second dose, the incidence of adverse reactions in medium-dosage group, high-dosage group and placebo group were 27.08%, 31.94% and 15.48% respectively, with statistical significance (P=0.0136). Most adverse reactions were mild (Grade 1) and only one severe (Grade 3) adverse reaction occurred in the high-dosage group. Most adverse reaction occurred within Day 0-7 after vaccination. The incidence of adverse reaction after the second dose was lower than the first dose. A total of 371 subjects received the COVID-19 vaccine/Placebo according to the routine immunization schedule (Day 0,28), and the incidence of medium-dosage group, high-dosage group and placebo group were 16.67%, 16.67% and 15.66% respectively without statistical significance (P=0.9546). Most adverse reactions were mild, without Grade 3 adverse reaction. Most adverse reaction occurred within Day 0-7 after vaccination. The adverse reaction incidence after the second dose of inoculation declined compared with the first dose.

For Phase II clinical trial, a total of 150 subjects received the third dose at day 28 after the two-dose schedule (Day 0, 14). From the beginning vaccination to 28 days after the third dose, the incidence of adverse reactions in medium-dosage group, high-dosage group and placebo group were 30.00%, 31.67% and 13.30% respectively, without statistical significance (P=0.2520). Most adverse reactions were mild, without Grade 3 adverse reaction. Most adverse reactions occurred within Day 0-7 after vaccination. The incidence of adverse reaction after the third dose was lower than the first dose and the second dose. A total of 150 subjects received the third dose at day 28 after the two-dose schedule (Day 0, 28), the incidence of adverse reactions in medium-dosage group, high-dosage group and placebo group were

18.33%, 18.33% and 23.33% respectively, without statistical significance (P=0.6210). Most adverse reactions were mild, without Grade 3 adverse reaction. Most adverse reaction occurred within Day 0-7 after vaccination. The adverse reaction incidence after the inoculation of third dose declined compared with the former two doses.

For Phase II clinical trial, within 28 days after the third dose of vaccination of 141 subjects according to the emergency immunization schedule, the adverse reactions were mainly pain,and the adverse reaction incidence rate was 12.06%. The adverse reaction incidence rates of medium and high dose groups of test vaccine and placebo group were 14.55%, 16.07.67% and 0% respectively.There was no significant difference in the incidence of other symptoms except for vaccination site pain.

Within 28 days, after 371 subjects were vaccinated with two doses of experimental vaccine/placebo according to the routine immunization schedule, the adverse reactions were mainly pain and the adverse reaction incidence rate was 10.00%. And the adverse reaction incidence of medium-dose group, high-dose group and placebo group of test vaccine were 11.54%, 14.00% and 0%, respectively. Followed by fever, with an incidence of 2.31%, and the incidence of medium-dose group, high-dose group and placebo group were 1.92%,2.00% and 3.57%, respectively. There was no significant difference in the incidence of symptoms among groups. The adverse reactions were mainly pain, followed by fatigue. As of 28 days after booster immunization, there was no SAEs unrelated to vaccination occurred in the Phase I/II clinical trials.

- **Indicators for laboratory testing**

In phase I clinical trial of COVID-19 Vaccine in adults aged 18-59 years, all subjects received blood routine examination, blood biochemistry and routine urine test 3 days before and after each dose of vaccine; the results showed that the incidence of abnormalities in all these laboratory indicators of clinical significance was low. For the emergency immunization schedule, the incidence of abnormalities in laboratory indicators of clinical significance was 8.33%, 8.33% and 4.17% respectively in test vaccine group, high-dose group and placebo group 3 days after each dose of vaccine; for the routine immunization schedule, such incidence of abnormalities was 8.33%, 8.33% and 4.35% respectively. There was no statistically significant difference in the incidence among groups receiving different immunization schedules, which was mainly of level 1.

- **Inflammatory factor**

In phase I clinical trial of COVID-19 Vaccine in adults aged 18-59 years, all subjects received inflammatory factor testing at different time points before and after immunization. For both emergency immunization schedule and routine immunization schedule, the IL-6, IL-2 and TNF-α varied within a small range and no significant rise of serum inflammatory factor was observed, which showed that the vaccine caused immunopathological effects with limited risks.

5.1.2 Immunogenicity evaluation

5.1.2.1 Immunogenicity of two doses

- **Result of neutralizing antibody**

The results of phase I clinical trial of COVID-19 vaccine in adults aged 18-59 years: For the emergency immunization schedule, the seroconversion rates were 45.83%, 50.00% and 0% respectively and

GMTs (1:) were 5.6, 7.7 and 2.0 respectively in medium-dosage group, high-dosage group and placebo group at Day 14 after the second dose; for the routine immunization schedule, the seroconversion rates were 83.33%, 79.17% and 4.35% respectively and GMTs (1:) were 19.0, 29.6 and 2.2 respectively in three groups on Day 28 after the second dose.

The results of phase II clinical trial of COVID-19 vaccine in adults aged 18-59 years showed that for the emergency immunization schedule (C001-C300), the seroconversion rates of neutralizing antibody were 92.37%, 98.32% and 3.33% respectively and GMTs (1:) were 27.6, 34.5 and 2.3 respectively in medium-dosage group, high-dosage group and placebo group at Day 14 after the second dose; for the routine immunization schedule (D001-D300), the seroconversion rates were 97.44%, 100% and 0% respectively and GMTs (1:) were 44.1, 65.4 and 2.0 respectively medium-dosage group, high-dosage group and placebo group at Day 28 after the second dose. According to the results, both the emergency immunization schedule and the routine immunization schedule of COVID-19 Vaccine had good immunogenicity. On Day 14 after the second dose according to the emergency schedule (C001-C300), the seroconversion rate in the high-dosage group was slightly higher than that in the medium-dosage group (P=0.0296), and GMT in the high-dosage group was comparable to that in the medium-dosage group (P=0.1051); on Day 28 after the second dose according to the routine schedule (D001-D300), the seroconversion rate in the high-dosage group was roughly the same as that in the medium-dose group (P=0.1218), and GMT in the high-dosage group was higher than that in the medium-dosage group (P=0.0006). In summary, the difference in neutralizing antibody GMT after immunization was less than 1.5 times between the medium and high dose groups, and the seroconversion rates were more than 90% in both groups.

The immunogenicity from phase II was significantly superior to that from phase I due to the use of different vaccine preparation processes in the trial; the vaccine used in phase I clinical trial was prepared by the cell factory, while that used in phase II clinical trial was prepared by fermenter; the COVID-19 Vaccine produced with the upgraded process showed good immunogenicity.

5.1.2.2 Immunization persistence of two doses

For the Day 0,14 two-dose immunization schedule of the Phase II clinical trial (C151-C300), the results 6 months after immunization show that the seropositive rates of middle-dosage group, high-dosage group and placebo group were 16.95%, 24.14% and 0% respectively, and GMTs were 4.1, 4.8 and 2.0 respectively. For the Day 0,28 two-dose immunization schedule of the Phase II clinical trial (D151-D300), the results 6 months after immunization show that the seropositive rates of medium-dose group, high-dose group and placebo group were 35.19%, 46.43% and 0% respectively, and GMTs were 6.7, 7.1 and 2.0 respectively. The results showed that the neutralizing antibodies 6 months after the two doses of vaccination based on different immunization schedules were lowered to a low level.

5.1.2.3 Immunogenicity results of booster dose

138 subjects in Phase II clinical trial received booster immunization 6 months after two doses of primary immunization on day 0,14. The results of 14 days after booster immunization showed that the seroconversion rates of the medium-dose group, high-dose group and placebo group were 100.00%, 100.00% and 0.00%, respectively. The seropositive rates were 98.11%, 100.00% and 0.00%, and the GMTs were 137.9, 175.1 and 2.00, respectively. There were statistically significant differences in seroconversion rates, seropositive rates, GMT and GMI among all groups after booster immunization,

while there were no statistically significant differences between medium-dose and high-dose groups.

124 subjects in Phase II clinical trial received booster immunization 6 months after two doses of primary immunization on day 0,28. The results of 28 days after booster immunization showed that the seroconversion rates of the medium-dose group, high-dose group and placebo group were 100.00%, 100.00% and 0.00%, respectively. The seropositive rates were 95.92%, 95.83% and 0.00%, and the GMTs were 143.1, 215.7 and 2.00, respectively. There were statistically significant differences in seroconversion rates, seropositive rates, GMT and GMI among all groups after booster immunization. Meanwhile, only GMT difference was statistically significant between medium-dose and high-dose groups.

5.1.3 Conclusion

The COVID-19 Vaccine produced by SINOVAC had good safety and immunogenicity in adults aged 18-59, and produced antibodies rapidly after the inoculation of both emergency immunization schedule and routine immunization schedule. Neutralizing antibodies decreased significantly 6 months after two doses of vaccination based on Day 0,14 emergency immunization schedule and the Day 0,28 primary immunization schedule. The level of neutralizing antibody increased significantly 6 months after two doses of primary immunization, suggesting a better immune memory response. In view of the above evidence, it provides a scientific basis for formulating the optimal immunization program.

5.2 Phase I/II clinical trial of COVID-19 vaccine in elderly

5.2.1 Safety Evaluation

In the Phase I/II clinical trial for the elderly aged 60 and older, a total of 421 subjects received at least one dose of COVID-19 vaccine/Placebo according to the Day 0,28 immunization schedule. The comprehensive safety analysis results of phase I and phase II within 28 days after vaccination of two doses showed that the incidence of adverse reaction were 20.00%, 20.00%, 21.95% and 20.55% respectively in low-dose group, medium-dose group, high-dose group and placebo group, and there was no statistically significant difference in the incidence among groups. The adverse reaction mainly occurred on Days 0-7 after the inoculation, and all adverse reactions were light and moderate, without Grade 3 adverse reaction. No adverse reaction increased or decreased obviously as the dose increased.

The adverse reactions were mainly vaccination site pain with the incidence of 11.00%, 11.20%, 8.94% and 4.11% respectively in low-dose group, medium-dose group, high-dose group and placebo group, and the incidence of adverse reactions of other symptoms was less than 5%. The incidence of headache and mucocutaneous herpes was slightly higher in the high-dose group than that in other three groups, and the decreasing incidence of placebo group is slightly higher than that of other three groups. Other symptom differences have no statistical significance.

As of 28 days after immunization booster, there was no SAEs unrelated to vaccination occurred in the Phase I/II trials.

5.2.2 Immunogenicity evaluation

5.2.2.1 Immunogenicity of two doses

For phase I clinical trial of COVID-19 vaccine in population aged 60 and above, on Day 28 after two doses of vaccine according to the Day 0,28 primary immunization schedule, the seroconversion rates

(≥1:8) of neutralizing antibody were 100.00%, 95.65% and 0.00% respectively, the GMT (1:) was 54.9, 64.4 and 2.0 respectively and GMI was 27.5, 32.2 and 1.0 respectively in medium-dose group, high-dose group and placebo group. For 28 days after the second dose in phase II, the seroconversion rates (≥1:8) of neutralizing antibody were 90.72%, 97.96%, 98.98% and 0.00% respectively, the neutralizing antibody of GMTs (1:) were 23.4, 42.2, 49.9 and 2.1 respectively and GMIs were 11.7, 20.9, 24.2 and 1.0 respectively in low-dose group, medium-dose group, high-dose group and placebo group. The results indicated that, the seroconversion rates of neutralizing antibody were all over 95% 28 days after the second dose of medium-dose vaccine or high-dose vaccine, with no dose-response relationship in medium-dose group and high-dose group, and the results were similar between phase I and phase II. In addition, the results indicated that the immunogenicity of medium-dose group and high-dose group was obviously superior to low-dose group, with a obvious dose-effect relationship.

5.2.2.2 Immunization persistence of two doses

The Phase II clinical trial showed that the seropositivity rates (⩾1:8) of the low-dose, medium-dose, high-dose and placebo groups were 12.63%, 17.35%, 22.58% and 2.13%, respectively, and the GMT was 3.1, 3.4, 4.1 and 2.1, respectively, 6 months after the second dose. The level of neutralizing antibody dropped to lower levels 6 months after two doses.

5.2.2.3 Immunogenicity results of booster immunization

287 subjects in Phase II clinical trial received booster immunization 6 months after two doses of primary immunization. The results showed that the seropositive rates were more than 97% at 7, 14 and 28 days after booster immunization; GMTs were 179.0, 305.0 and 418.8 in the low-dose, medium-dose and high-dose groups 7 days after immunization, 206.9, 318.3 and 689.1 14 days after immunization, and 184.6, 342.8 and 437.7 28 days after immunization. The results showed GMT in the high-dose group > the medium-dose group > the low-dose group at different time points after booster immunization, and antibody levels in each group increased rapidly and significantly after booster immunization.

5.2.3 Conclusion

According to Day 0,28 immunization schedule, the COVID-19 vaccine produced by SINOVAC was safe in elderly aged 60 and older. For immunogenicity evaluation, medium dosage group and high dosage group were comparable and better than low dosage group. In the meantime, the research results are similar to the results of Phase II clinical trial of COVID-19 vaccine for adults. Considering that the neutralizing antibody decreased to a low level 6 months after two doses of immunization, a booster dose was added 6 months after the two doses of immunization, and the results showed that the level of neutralizing antibody increased rapidly and significantly, indicating a good immune memory response. Based on the above evidences, it provides scientific basis for formulating the optimal immunization program.

5.3 Phase I/II clinical trial of COVID-19 vaccine in children and adolescents

5.3.1 Safety Evaluation

A total of 550 subjects aged 3-17 years old received at least one dose of test vaccine or placebo in the phase I/II clinical trial. At present, adverse events have been collected within 28 days after the second dose in both phase I and Phase II. The comprehensive safety analysis results of Phase I and Phase II showed that the incidence of adverse reactions in the low-dose, medium-dose and placebo groups was

25.57%, 29.03% and 23.68%, respectively, within 28 days after vaccination of two doses, with no statistically significant difference in incidence among the groups. The adverse reactions were mainly grade 1 and 2, and the incidence of grade 3 adverse reactions was only 0.36%. Most adverse reaction occurred within Days 0-7 after vaccination. The incidence of adverse reactions after the first dose in the low-dose group was similar to that after the second dose in the low-dose group, and slightly higher than that after the first dose in the medium-dose group and the placebo group, showing no significant trend of increasing or decreasing adverse reactions with the increase of dose. The incidence of adverse reactions was 16.44%, 16.13% and 1.75% in the low-dose, medium-dose and placebo groups, respectively. The incidence of adverse reactions in the low-dose group and medium-dose group was higher than that in the placebo group, and the main reason was that the incidence of grade 1 pain in the low-dose and medium-dose groups was higher than that in the placebo group. The overall incidence of other symptoms was less than 5%, there was no significant difference in the incidence of symptoms among groups. As of 6 months after vaccination of two doses, there was no SAEs related to vaccination.

5.3.2 Immunogenicity evaluation

5.3.2.1 Immunogenicity of two doses

28 days after the second dose in Phase I clinical trial, the seroconversion rates of neutralizing antibody (≥1:8) in low-dose, medium-dose and placebo groups were 100.00%, 100.00% and 0.00%, respectively, and GMT(1:) was 55.0, 117.4 and 2.0, respectively. 28 days after the second dose in Phase II clinical trial, the seroconversion rates of neutralizing antibody (≥1:8) in low-dose, medium-dose and placebo groups were 96.77%, 100.00% and 0.00%, respectively, and GMT(1:) was 86.4, 142.2 and 2.0, respectively. The results showed that the seroconversion rates of neutralizing antibody were above 96% in the low-dose and medium-dose groups 28 days after the full immunization, and the GMT in medium-dose group was superior to that in low-dose group.

Further analysis by age group showed that the seroconversion rates after receiving low dose and medium dose of vaccine in 3-5 years old, 6-11 years old and 12-17 years old subjects reached more than 92%. In Phase I clinical trials, GMT decreased with the increase of age after the vaccination of low dose or medium dose. GMT (1:) of the vaccination of low dose was 71.9, 50.5 and 45.9 in subjects aged 3-5, 6-11 and 12-17, and GMT (1:) of the vaccination of medium dose was 212.6, 101.6 and 70.8, respectively, in subjects aged 3-5, 6-11 and 12-17. In phase II clinical trials, GMT did not increase or decrease with the increase of age after vaccination, and GMT (1:) of the vaccination of low dose was 94.1, 90.3 and 78.3, and GMT (1:) of the vaccination of medium dose was 140.5, 139.7 and 146.0, respectively, in subjects aged 3-5, 6-11 and 12-17. In phase I clinical trials, the trend of GMT changing with age may be caused by the small sample size. In addition, GMT of the vaccination of medium dose was superior to that of the vaccination of low dose vaccine in all age groups.

5.3.2.2 Immunization persistence of two doses

For 227 subjects who included in analysis of immunization persistence of 3 months after the second immunization in the Phase II clinical trial, the seropositivity rates of neutralizing antibody in the low-dose and medium-dose vaccine groups were 98.91% and 100.00%, respectively, and the GMT was 67.8 and 110.5, respectively. Further analysis by age group showed that the seropositivity rates of neutralizing antibody were above 97% in the low-dose and medium-dose groups of 3-5, 6-11 and 12-17 years old subjects. At the same time, GMT in all age groups did not have a significant decreased

trend, especially for 3-5 years old children, GMT at 3 months after the second immunization was similar to that of 28 days after the full immunization.

For 233 subjects who included in analysis of immunization persistence of 6 months after the second dose in Phase II clinical trial, the seropositivity rates of neutralizing antibody in low-dose vaccine group and placebo group were 86.81% and 95.74%, respectively, and GMT was 21.9 and 27.2, respectively. Further analysis by age group showed that the seropositivity rates of 3-5 years old subjects in both groups were 100%, and the GMTs were 36.6 and 48.0, respectively. The seropositivity rates of 6-11 years old subjects in low-dose vaccine group and placebo group were 94.29% and 100.0%, and GMT was 24.8 and 29.0, respectively. The seropositivity rates of 12-17 in low-dose vaccine group and placebo group were 69.70% and 88.28%, and GMTs were 13.5 and 17.0, respectively. Compared with 28 days and 3 months after the second dose, the seropositivity rates of neutralizing antibody in subjects aged 3-11 years old remained at a high level, but GMT decreased significantly. The seropositivity rates and GMT of neutralizing antibody decreased significantly in subjects aged 12~17 years old.

The results showed that neutralizing antibody levels did not decrease significantly 3 months after the two doses of vaccination in subjects aged 3-17. Indeed, neutralizing antibody levels decreased significantly 6 months after the two doses of vaccination, especially in subjects aged 12-17. But antibody levels 6 months after the two doses of vaccination was higher than results in adults and elderly.

5.3.3 Conclusion

The Phase I/II clinical trial of COVID-19 Vaccine in children and adolescents showed that the COVID-19 Vaccine produced by Sinovac has good safety and immunogenicity in healthy people aged 3-17 years based on Day 0, 28 immunization schedule. The safety in low-dose and medium-dose groups was similar; The immunogenicity of medium dose group was better than that of low dose group.

Based on trends of antibody levels at different time points after the full immunization, the Data Monitoring Committee (DMC) concluded that the available data supported the vaccination of booster immunization in children and adolescents. Therefore, Sinovac plans to carry out booster immunization in children and adolescents, so as to further explore the effect of booster immunization schedule and provide evidence for the formulation of optimal immunization strategies.

6 Product Features

6.1 Preparation Technology and Formulation of the Vaccine

The COVID-19 Vaccine, Inactivated is prepared from novel coronavirus (CZ02 Strain), which is inoculated on African green monkey kidney cells (Vero Cells), then cultured, harvested, inactivated, concentrated, purified and finally aluminium absorbed. The finished vaccine is a milky white suspension liquid, which can be layered due to precipitation and easily dispersed. The main component of the vaccine is the inactivated SARS-COV-2, with the excipients of aluminum hydroxide, disodium hydrogen phosphate, sodium dihydrogen phosphate, sodium chloride, etc., and the vaccine is preservative-free. The vaccine is packaged with prefilled syringes or vials, 0.5ml for each container. The vaccine can induce the immunity against the SARS-COV-2, which can prevent the disease caused by the SARS-COV-2 infection.

The investigational vaccine is manufactured by Sinovac Life Sciences Co., Ltd., Ltd. and tested eligible by National Institute for Food and Drug Control according to *Manufacturing and Quality Control Requirements of Inactivated SARS-CoV-2 Vaccine（Vero Cell) (Draft Version)*. The vaccine is

injectable with the specification of 0.5mL/container. The antigen content of low, medium and high dosage vaccine is 300SU, 600SU, and 1200SU/0.5mL respectively.

6.2 Stability

The thermal accelerated stability study at 25±1°C for 42 days and at 37±1°C for 42 days has been completed for 6 batches of finished products produced by the bioreactor. When stored at 25±1°C, the antigen content after the dissociation at different time points within 42 days (inclusive) met the quality standard, and the monitoring of antigen content after the dissociation will continue for 56 days; when stored at 37±1°C, the antigen content after the dissociation at different time points within 28 days (inclusive) met the quality standard, while the antigen content after the dissociation at the monitoring point on Day 42 was lower than 50% of the labelled amount for some batches, then the test was terminated.

The long-term stability observation at 2-8°C for 3 months has been completed for 12 batches of finished products under the cell factory and bioreactor manufacturing processes, and no obvious reduction was observed in the antigen content after the dissociation of finished products of COVID-19 Vaccine packaged in the syringe. The antigen content after the dissociation of finished products of COVID-19 Vaccine packaged in the penicillin bottle declined slightly compared with the Day 0 result. Based on the analysis, the packaging material absorbs the antigen to a certain degree, and its stability will be further investigated successively.

According to the results of accelerated stability test, the validity period of vaccine is tentatively determined to be 2 years when stored at 2-8°C. The stability test will continue for batches of final products of COVID-19 vaccine as per the stability study plan.

6.3 Control Vaccine

In this study, placebo produced by Sinovac Life Sciences Co., Ltd. was adopted as the control. The placebo is aluminum hydroxide diluent with trace of milkly white precipitation. The appearance is consistent with the investigational vaccine.

It is tested eligible by National Institute for Food and Drug Control according to the *Manufacturing and Quality Control Requirements of Inactivated SARS-CoV-2 Vaccine （Vero Cell） (Draft Version)*. The vaccine is injectable with the specification of 0.5mL/container. It contains no SARS-CoV-2 antigen.

6.4 Transportation and Storage of Vaccine

The vaccine shall be kept and transported away from light at 2~8°C. When the excessive temperature occurs temporarily for such reason as the signing after reception of vaccine, the opening/closing of door and other normal operations, it shall not be considered as deviation.

6.5 Inoculation route and procedure

The qualified subjects receive intramuscular injection at the lateral deltoid of upper arm, each with 0.5ml test vaccine or control vaccine for single dose. They were inoculated as per D0/28 two-dose primary immunization schedule, at 0.5ml/dose/time; the vaccine was shook well before use.

Subjects in the low-dose and medium-dose vaccine groups in the Phase II clinical trial received one

dose of booster immunization 10 or 12 months after vaccination of two doses.

6.6 Information of Investigational Vaccine

Information of Investigational Vaccine is shown in the following figure:

Table 8 Information of Test Product

Group	Vaccine name	Packaging	Antigen content	Manufacturer	Lot No.	Valid until
Low dosage vaccine	COVID-19 Vaccine, Inactivated	Prefilled syringe	300SU/ 0.5ml	SINOVAC	20200307	2023.03.20
Medium dosage vaccine	COVID-19 Vaccine, Inactivated	Prefilled syringe	600SU/ 0.5ml	SINOVAC	20200412	2023.04.08
Placebo	Aluminum hydroxide diluent	Prefilled syringe	0SU/ 0.5ml	SINOVAC	20200228 01	2023.02.27

6.7 Vaccine Packaging

The vaccine will be packed in a labelled box, with the label style shown as follows, and the numbering principle of vaccine can be found in "9.4 Randomization and blindness".

Phase I/II of Clinical Trial of SARS-CoV-2 Vaccine<br>(Vero Cell), Inactivated<br>PRO-nCOV-1003<br>**C001**<br>Only for clinical study, stored at 2-8°C<br>Expiration date:

The packing box should be as follows:

Phase I/II of Clinical Trial of SARS-CoV-2 Vaccine<br>(Vero Cell), Inactivated<br>PRO-nCOV-1003<br>Serial No.:<br>Only for clinical study, stored at 2-8℃<br>Expiration date:

7 Objective

To evaluate the safety and immunogenicity of COVID-19 Vaccine developed by SINOVAC in health children and adolescents aged 3-17 years old.

7.1 Phase I clinical trial

To evaluate the safety, tolerance and preliminary immunogenicity of different dosage vaccine in healthy population aged from 3 to 17 years.

7.2 Phase II clinical trial

To evaluate the safety and immunogenicity of different dosage vaccine in healthy population aged from 3 to 17 years to determine the appropriate dosage for further clinical evaluation.

8 Study Design

8.1 Design

8.1.1 Overall design

The randomized, double-blinded and placebo-controlled design is adopted.

8.1.2 Sample Size Considerations

Phase I clinical trial: according to the Technical Guidelines for Clinical Trial of Vaccines[3] and *Provisions of Drug Registration*[1], Phase I clinical trial is a small-scale research (20~30 persons), focusing on the evaluation of vaccine safety. The total sample size of clinical trials at this stage is 72, and the total number of people vaccinated with low and medium doses of test vaccines is 54, and the number of people vaccinated meets the requirements of Phase I clinical trials.

Phase II clinical trial: according to the *Technical Guidelines for Clinical Trial of Vaccines*[3] and the *Provisions of Drug Registration*[1], Phase II clinical trial is to observe the immunization effect and safety of different doses of vaccine in the target population. The endpoint is to evaluate the immunogenicity and safety of the test vaccine. The number of cases in the test group is not less than 300. The total sample size of clinical trials at this stage is 480, and the total number of people vaccinated with low and medium doses of test vaccines is 384, and the number of people vaccinated meets the basic requirements of Phase II clinical trials.

8.2 Endpoint

8.2.1 Endpoints of phase I trial

8.2.1.1 Primary endpoint

– Incidence of adverse reactions 0~28 days after each dose of vaccination;

8.2.1.2 Secondary endpoint

- Incidence of adverse reactions 0~7 days after each dose of vaccination;
- Incidence of abnormal laboratory indexes (blood routine test, blood biochemistry test and urine routine test) on the 3rd day after each dose of vaccination;
- The incidence of SAE from the vaccination to 12 months after the second dose.
- Seroconversion rate, seropositive rate, GMT and GMI of neutralizing antibodies on Day 28 after the second dose;
- Seroconversion rate, seropositive rate, GMT and GMI of neutralizing antibodies on Day 28 after the first dose;

8.2.1.3 Exploratory endpoint

- Seropositive rate and GMT of neutralizing antibodies 6 and 12 months after the second dose.

8.2.2 Endpoints of phase II trial

8.2.2.1 Primary endpoint

- Seroconversion rate of neutralizing antibodies on Day 28 after the second dose;

- Incidence of adverse reactions 0~28 days after each dose of vaccination.

8.2.2.2 Secondary endpoint

- Seroconversion rate, GMT and GMI of neutralizing antibodies on Day 28 after the second dose;
- Incidence of adverse reactions 0~7 days after each dose of vaccination;
- The incidence of SAE and AESI from the beginning of vaccination to 12 months after the booster immunization.

8.2.2.3 Exploratory endpoint

- Seropositive rate and GMT of neutralizing antibodies 3, 6 ,10 and 12 months after the second dose.
- Seropositive rate, GMT and GMI of neutralizing antibody against Prototype strain (CZ strain) and Omicron 28 days after booster dose.
- Seropositive rate and GMT of neutralizing antibody against Prototype strain (CZ strain) and Omicron strain 6 and 12 months after booster dose.

8.3 Study Plan

8.3.1 Study Plan of Phase I

The clinical trial is a single-center, randomized, double-blind and placebo-controlled study. In Phase I, 72 adolescents and children aged 3-17 years (including 24 subjects aged 3-5 years, 6-11 years and 12-17 years respectively) will be selected. Sequential grouping method will be adopted according to the age from older to younger and the dose-escalating manner is used. The subjects in different dose stages of each age group will be randomly divided into 2 groups according to the ratio of 3: 1. Each enrolled subject will receive two doses of vaccine or placebo according to the immunization schedule of day 0, 28. Enrollment in Phase I clinical trial is divided into four stages as follows: the first stage: low-dose stage (12 subjects) for adolescents aged 12-17; the second stage: medium-dose stage for adolescents aged 12-17 (12 subjects) and low-dose stage for children aged 6-11 (12 subjects); the third stage: medium-dose stage (12 subjects) for children aged 6-11 and low-dose stage (12 subjects) for children aged 3-5; the fourth stage: medium-dose stage for children aged 3-5 (12 subjects). The next stage will start only with the condition that safety observation 0~7 days after the first dose of vaccination is finished, and the good safety profiles is confirmed according to the occurrence of the solicited and unsolicited adverse events, as well as the occurrence of the abnormal results of the blood routine, blood biochemical and urine routine testing.

The immediate reactions within 30 min after each dose of inoculation will be observed; the solicited local and systemic adverse events within 0~7 days and the unsolicited adverse events within 0~28 days will be collected; and the SAE and AESI monitoring will be from the beginning of vaccination to 12 months after the second dose.

Venous blood and urine sample will be collected from all subjects at different time points before and after vaccination for the blood routine, blood chemistry, urine routine testing, and the testing of serum inflammatory factor and antinuclear antibody, to evaluate the safety. Venous blood will be collected from all subjects at different time points before and after vaccination for the neutralizing antibody test, to evaluate immunogenicity and immune persistence.

The detailed study plan of phase I clinical trial is shown in the table 9.

Table 9 Study Plan of Phase I Clinical Trial

Enrollment stage	Age group	Dose stage	Low dose	Medium dose	Placebo	Total	Immunization schedule (day)	Neutralizing antibody test (day)	Blood routine examination, blood biochemistry and routine urine test (day)
Stage 1	12~17 years old	Low dose stage	9	-	3	12	0,28	0,28,56, 208,388	0(-3),3,28,31
Stage 2	12~17 years old	Medium dose stage	-	9	3	12			
	6~11 years old	Low dose stage	9	-	3	12			
Stage 3	6~11 years old	Medium dose stage	-	9	3	12			
	3~5 years old	Low dose stage	9	-	3	12			
Stage 4	3~5 years old	Medium dose stage	-	9	3	12			
**Total**			**27**	**27**	**18**	**72**			

8.3.2 Study Plan of Phase II

The subjects for Phase II clinical trial can be enrolled only upon safety observation on Days 0-7 after the first dose of inoculation in all medium-dose groups for Phase I clinical trial and the safety confirmation by the Data Monitoring Committee (DMC). A total of 480 adolescents and children aged 3-17 years (including 120, 180, 180 subjects aged 3-5 years, 6-11 years and 12-17 years respectively) will be selected. The subjects of each age sub-group will be randomly divided into 3 groups according to the ratio of 2:2:1. All enrolled subjects will receive two doses of low-dose, medium-dose test vaccine and placebo respectively.

Based on the results of immunogenicity 6 months after 2 doses from the Phase Ⅱ trial, DMC concluded that the available data support the introduction of booster immunization in children and adolescents. Therefore, the study plans to administrate one booster dose to subjects in the low -dose and medium-dose vaccine groups 10 or 12 months after the second dose, with those numbered C101-C190, C281-C370 and C461-C520 receiving a booster dose 10 months after the second dose, those numbered C191-C280, C371-C460 and C521-C580 receiving a booster dose 12 months after the second dose.

The immediate reactions within 30 min after each dose of inoculation will be observed; the solicited local and systemic adverse events within 0~7 days and the unsolicited adverse events within 0~28 days will be collected; and the SAE and AESI monitoring will be from the beginning of vaccination to 12 months after the second dose.

Blood samples will be collected at different time points before and after immunization to detect neutralizing antibodies, evaluate the immunogenicity, immunization persistence of the vaccine, and the immune effect of booster immunization.

The detailed study plan of phase II clinical trial is shown in the table 10.

Table 10 Study Plan of Phase II

Test group	3~5 years old	6~11 years old	12~17 years old	Total	Primary immunization schedule (day)	Booster immunization schedule (day)	Time of blood sample collection (day)
**Low-dose group**	48	72	72	192	0,28	One dose of booster immunization 10 months or 12 months after the second dose	0,56,118,328, 356, 508,688 or 0,56,208,388, 416,568,748*
**Medium-dose group**	48	72	72	192			
**Placebo group**	24	36	36	96	0,28	-	0,56,118 或 208¶
**Total**	120	180	180	480	-	-	

§ Booster immunization for C101-C190,C281-C370 and C461-C520 subjects 10 months after the vaccination of the second dose .And booster immunization for C191-C280, C371-C460 and C521-C580 subjects 12 months after the vaccination of the second dose.

* Blood sampling time of subjects numbered C101-C190, C281-C370 and C461-C520 was 0,56,118,328, 356, 508,688; Blood sampling time of subjects numbered C191-C280, C371-C460 and C521-C580 was 0,56,208,388, 416,568,748.

* Blood sampling for C101-C190, C281- C370 and C461-C520subjects 3 months after the second dose; Blood sampling for C191-C280, C371-C460 and C521-C580 subjects 6 months after the second dose.

8.4 Randomization and blinding

8.4.1 Randomization

Randomized statisticians adopt the block randomization method. The SAS software (Version 9.4) is used to stratify and randomize the subjects of Phase I and Phase II clinical trials. A randomized blind code is generated. The blind code of the research vaccine is a "List of Corresponding Relationships between Random Numbers and Research Vaccines or Placebos", which is made in duplicate and sealed after blind coding is completed. The original is kept by the investigator for unblinding in the test, and the copy is kept by the Sponsor. Phase I clinical trial vaccine number is C001-C072, and Phase II clinical test vaccine number is C101-C580.

Randomized statisticians apply SAS software (Version 9.4) to generate blind codes of spare vaccines for Phase I and Phase II clinical trials respectively. The low-dose, medium-dose and placebo spare vaccines for Phase I clinical trials are prepared according to the ratio of 3:3:2, and the spare vaccines are numbered X001-X016; the low, medium dose and placebo spare vaccines in Phase II clinical trials are prepared according to the ratio of 2:2:1, and the spare vaccines are numbered Y001-Y050. When the color of the test vaccine changes or the test vaccine is damaged, the vaccination personnel shall report to the on-site responsible investigator and the principal investigator, start the standby vaccine activation procedure, obtain the standby vaccine number through the online standby vaccine acquisition system, and replace the research vaccine with the standby vaccine. All test vaccines and placebos will be labelled blind as described in “6.7 Vaccine Packaging”. After enrollment, subjects were inoculated with the blinding vaccine consistent with their study number.

8.4.2 Blinding

The blind design is adopted in this trial. Randomized statisticians and other blind coding personnel who are not involved in the trial are employed for blind coding of the vaccine, that is, paste the printed label on the designated position of each vaccine/placebo according to the blind code. Randomized statisticians supervise the vaccine blind coding, and guide the blind coding operators to label according to the blind code. After the blind coding is completed, the blind code shall be sealed by randomized statisticians. The whole blind coding process must be recorded in writing. The blind coding personnel shall neither participate in other related work of this clinical trial, nor disclose any information about

the blind code to any person participating in this clinical trial.

8.4.3 Emergency unblinding

Randomized statisticians shall prepare emergency letters during blind coding. Each letter shall contain a random password for unblinding, and each random password can correspond to any study number. The group of the study number can be fed back through the online unblinding system. Each random password represents an opportunity for unblinding. Only one study number can be subject to unblinding, and then it will become invalid. It is invalid for the study number that has been subject to unblinding. In this trial, 5 emergency envelopes are prepared for Phase I and 10 emergency envelopes are prepared for Phase II, which are kept by the investigator in charge of the site. The blind review personnel shall check the opening and closing status of emergency envelopes.

During the study period, if emergency unblinding is jointly determined by the principal investigator and the Sponsor, the site investigator shall open and read the emergency letter, log into the online unblinding system with the random unblinding password in the letter, perform emergency unblinding according to the prompt information, and make relevant records. The subjects with this study number will suspend the trial for withdrawal treatment, and the investigator will record the reasons for suspension in the CRF. The emergency letters that have been opened and read shall be kept properly and returned to the Sponsor after the trial.

8.4.4 Unblinding regulations

The unblinding of Phase I and Phase II clinical trials shall follow the time points below: obtaining the serum detection results on Day 28 after Day 0,28 primary immunization. It shall be implemented jointly by the Sponsor, the principal investigator and the statistician, and the unblinding record shall be kept. After the unblinding, the investigator responsible for observing and assessing the subjects and the clinical research associate responsible for source data validation shall remain blind until the database is locked. In order to protect the rights and interests of subjects in the placebo group and ensure that subjects can receive COVID-19 vaccine as soon as possible, subjects of appropriate age will be vaccinated with COVID-19 vaccine (non-experimental vaccine) in accordance with government requirements during the trial. The low-dose and medium-dose groups will remain blind until the final database is locked.

8.5 Flow chart

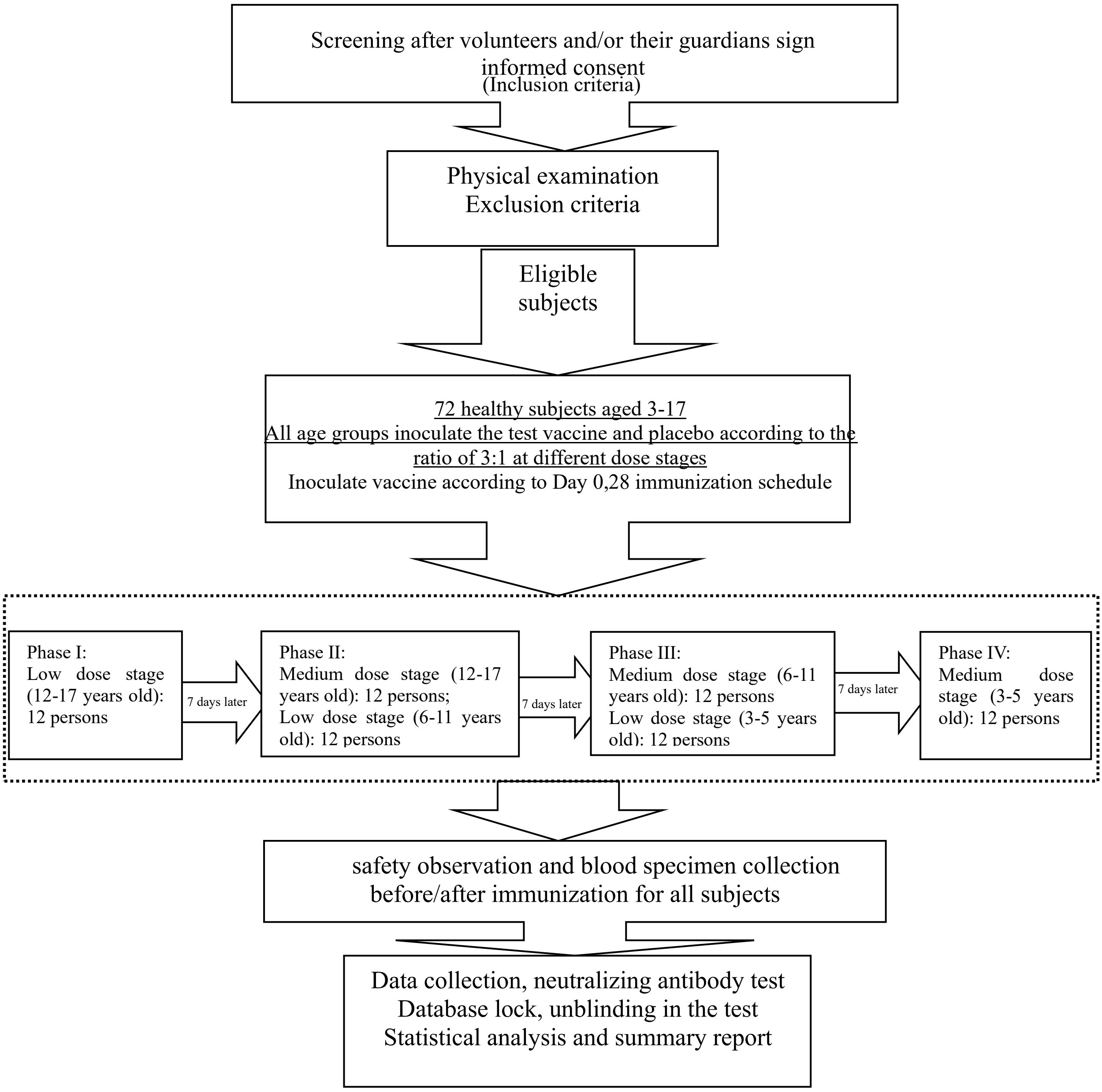

Figure 7 Flow Chart of Phase I clinical trial of COVID-19 Vaccine, Inactivated

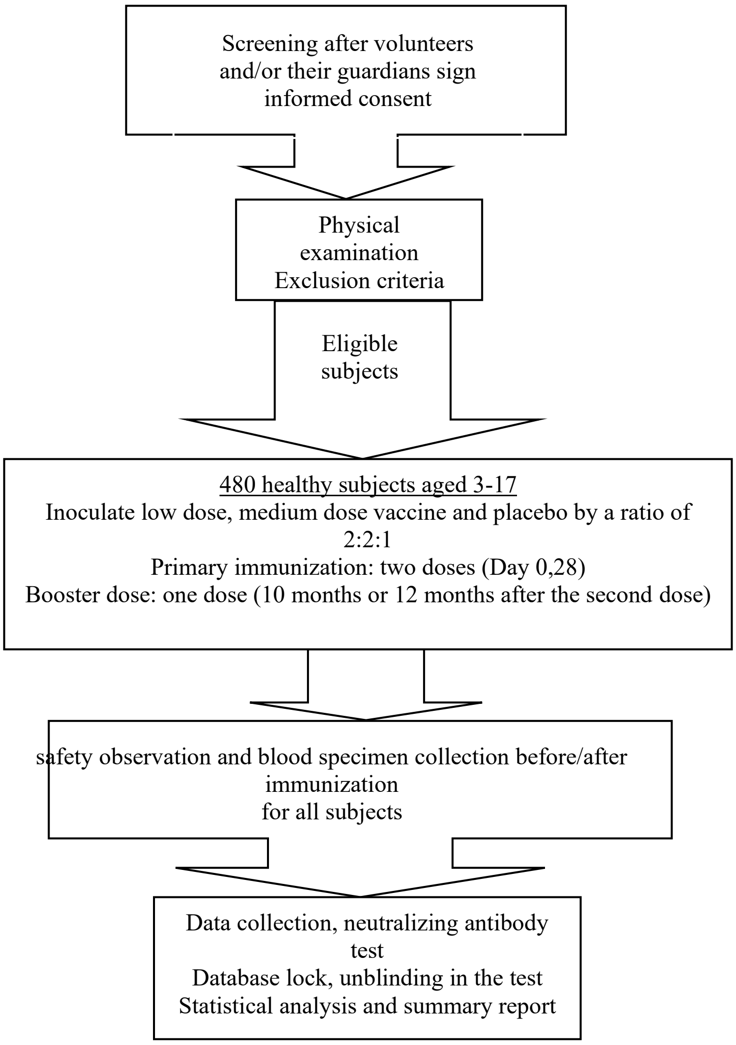

Figure 8 Flow Chart of Phase II clinical trial of COVID-19 Vaccine, Inactivated

8.6 Study Duration

It is estimated that the minimum study duration is 13 months for each subject.

8.7 Trial Suspension and Early Termination

Criteria for test suspension:

- One or more Grade-4 adverse reactions (local, systemic) related to vaccination;
- Over 15% of the subjects have Grade-3 or higher adverse reactions, including local reaction, systemic reaction, and vital signs.

Criteria for early termination:

- After the clinical trial is suspended, the Investigator and Sponsor will discuss and decide whether the trial is going to be terminated;
- The sponsor requests a complete termination of the test and gives reasons;
- Independent Ethics Committee requests a complete termination of the test and gives reasons;
- The competent administrative department requests a complete termination of the test and gives reasons.

8.8 Protocol Violation and Deviation

Protocol Deviation: It refers to any change and non-compliance with the design or process in the protocol for clinical trial. The protocol deviation refers to the action of evaluating the safety or main indicators without affecting the rights and interests, safety and benefits of the subjects, or the integrity, accuracy and reliability of the test data. The severe protocol deviation (protocol violation) refers to the action of evaluating the safety or main indicators affecting the rights and interests, safety and benefits of the subjects, or the integrity, accuracy and reliability of the test data.

For any protocol deviation/violation during the research, the on-site investigator shall report the fact, process, cause and impact to the responsible organization, and the Principal Investigator shall give opinions on the handling of such deviation/violation. The protocol deviation/violation report shall be submitted to the Independent Ethics Committee for review and approval.

The investigator shall provide targeted training on relevant links of such deviation/violation for the staff concerned to prevent the occurrence of similar incidents, and shall record the training process.

8.9 Pregnancy event

Pregnancy is an exclusion criterion for subjects, and women of childbearing age (after menarche (≥ 14 years old) and before menopause) are required to take effective contraceptive measures from the first dose of vaccination to 1 month after primary immunization and 1month after booster immunization, but the subjects may still have unwanted pregnancy during the participation process. The pregnancy events occurred within one month from the first dose of vaccination to the whole vaccination should be reported, and the researchers should fill in the "pregnancy event report form".

The researchers closely followed pregnant subjects to obtain information about pregnancy outcomes (e.g., details of delivery and new born conditions or termination of pregnancy), and updated the pregnancy event report form.

Pregnancy itself is not considered a serious adverse event, but any complications during pregnancy will be considered as an adverse event, and some cases can be considered as serious adverse event, such as spontaneous abortion, stillbirth and congenital abnormalities of infants. Induced abortion due to the mother's personal decision without any abnormalities in the fetus is not considered as an adverse event.

The treatment of pregnancy events during vaccination is as follows:

- If the pregnancy occurs after the first dose of vaccination and the whole vaccination has not been completed, the subjects are not allowed to participate in the follow-up visit until the end of pregnancy (term, abortion, abortion, etc.). Participants will be contacted regularly for pregnancy assessment.

If pregnancy is found after the subject has completed the whole course of vaccination, the subject can complete the study visit according to the trial scheme and the decision of the researcher.

9 Subjects

9.1 Inclusion Criteria for Subjects

(1) Healthy subjects aged 3~17 years old;
(2) The subjects/guardians are able to understand and sign the informed consent voluntarily (both subjects and guardians should sign the informed consent for those aged 8~17 years old);
(3) Provide legal identification.

9.2 Exclusion Criteria for Subjects

(1) Travel / residence history of communities with COVID-19 case reports within 14 days prior to the entry;
(2) Contact with SARS-CoV-2 infected persons (positive for nucleic acid detection) within 14 days prior to the entry;
(3) Contact patients with fever or respiratory symptoms from communities with case reports within 14 days prior to the entry;
(4) Two or more cases of fever and / or respiratory symptoms in a small area, such as family, office, school class or other places within 14 days prior to the entry;
(5) History of SARS-CoV-2 infection;
(6) History of asthma, allergy to vaccines or vaccine ingredients, and serious adverse reactions to vaccines, such as urticaria, dyspnea, angioneuroedema;
(7) Congenital malformation or developmental disorder, genetic defect, severe malnutrition, etc;
(8) Autoimmune disease or immunodeficiency / immunosuppression;
(9) Serious chronic disease, serious cardiovascular disease, hypertension and diabetes that cannot be controlled by drugs, hepatorenal disease, malignant tumor, etc;
(10) Serious nervous system disease (epilepsy, convulsion or convulsion) or psychosis;
(11) Thyroid disease or history of thyroidectomy, spleenlessness, functional spleenlessness, spleenlessness or splenectomy resulting from any condition;
(12) Diagnosed abnormal blood coagulation function (eg, lack of blood coagulation factors, blood coagulopathy, abnormal platelets) or obvious bruising or blood coagulation;
(13) Immunosuppressive therapy, cytotoxic therapy, inhaled corticosteroids (excluding allergic rhinitis corticosteroid spray therapy, acute non-complicated dermatitis superficial corticosteroid therapy) in the past 6 months;
(14) Abnormal hematological and biochemical laboratory test results beyond the reference value range

in physical examination (only applicable to phase I clinical trial)

1) Blood routine indexes: white blood cell count, hemoglobin, platelet count;

2) Blood biochemical indexes: alanine aminotransferase (ALT), aspartate aminotransferase (AST), total bilirubin (TBIL), creatinine (CR), fasting blood glucose;

3) Urine routine index: urine protein (PRO)

(15) Long history of alcohol or drug abuse;
(16) Receipt of blood products in the past 3 months;
(17) Receipt of other investigational drugs in the past 30 days;
(18) Receipt of attenuated live vaccines in the past 14 days;
(19) Receipt of inactivated or subunit vaccines in the past 7 days;
(20) Acute diseases or acute exacerbation of chronic diseases in the past 7 days;
(21) Axillary temperature >37.0°C;
(22) Under Pregnancy (including positive urine pregnancy test) or breast-feeding period and plan to prepare for pregnancy within 3 months;
(23) According to the investigator's judgment, the subject has any other factors that are not suitable for the clinical trial.

9.3 Exclusion Criteria for Vaccination with Subsequent Dose

The subjects who experience any of events in the following (1) to (5) are forbidden to continue vaccination, but they can continue other study steps according to the investigator' judgement. For the subjects who experience any of the events in the following (6) to (7), the investigator will judge whether vaccination will be continued. For the subjects who experience any of the events in the following (8) to (11), the vaccination can be delayed within the protocol-permitted time window.
(1) Similar vaccines other than the investigational vaccines were used during the study;
(2) Any serious adverse reactions which have a causal relationship with the vaccination;
(3) Severe anaphylaxis or hypersensitivity after vaccination (including urticaria/rash appears within 30 minutes after vaccination);
(4) Any confirmed or suspected autoimmune disease or immunodeficiency disease, including human immunodeficiency virus (HIV) infection;
(5) Become pregnant after last vaccination (including positive urine pregnancy test);
(6) Acute or newly onset chronic disease after vaccination;
(7) Other reactions (including severe pain, severe swelling, severe limitation of movement, persistent high fever, severe headache or other systemic or local reactions) judged by the investigators;
(8) Acute diseases occur during vaccination (acute disease means moderate or severe disease with or without fever);
(9) Axillary temperature >37.2℃ (aged >14 years) or >37.4℃ (aged 3~14 years) during vaccination;
(10) Have vaccinated with subunit vaccine or inactivated vaccine within 7 days, immuned with live attenuated vaccine within 14 days;
(11) According to the investigator's judgment, the subject has any other factors that affect vaccination.

9.4 Withdrawal and Termination Criteria for Subjects

(1) Subjects request to withdraw;
(2) Intolerable adverse events, whether or not related to the investigational product;
(3) Subjects are not allowed to participate in this trial due to their health status;
(4) In case of any abnormal clinical manifestations of the subjects, the researcher should determine whether it is related to the vaccine, and judge whether the subjects suspend the clinical trial;
(5) Any other reasons considered by the investigator.

If the trial vaccine has been inoculated to the subject before suspending, the clinical trial data of the subject will be used for safety analysis. Subjects could not be replaced in the trail. After the subjects who have been vaccinated in the clinical trial withdraw or suspend the trial, the researcher should provide necessary guidance for any clinical situation related to the trial, and follow up until a definitive diagnosis is obtained, or the health condition stabilizes or recovers.

10 Method and Schedule

10.1 Visit Plan

10.1.1 Visit plan of Phase I

Table 11 Visit plan of Phase I

**Follow-up**		**1**	**2**	**3**	**4**	**5**	**6**	**7**	**8**	**9**
Follow-up period	D-14～D-1	D-3～D0	D3[e]	D8[e]	D28[e]	D31[e]	D36[e]	D56[e]	D208[e]	D388[e]
Preliminary notice, subject recruitment	X									
Informed consent		X								
Demographic analysis		X								
General examination		X								
Urine pregnancy test (those with menophania, ≥14 years old)		X			X					
Blood routine examination, blood biochemistry		X	X		X	X				
Routine urine test		X	X		X	X				
Screening based on inclusion/exclusion criteria[a]		X			X					
Neutralizing antibody test		X			X			X	X	X
Inoculation[b]		X			X					
Subjects record the safety observation results on their daily diary/contact cards[c].		X	X	X	X	X	X	X		
Monitoring of adverse reactions/events (including adverse events of grade 3 and above and SAE,AESI)[cd]		X	X	X	X	X	X	X	X	X
Usage records of concomitant drugs/vaccines[cd]		X	X	X	X	X	X	X	X	X

a) Screening shall be conducted based on inclusion/exclusion criteria before vaccination of every dose.
b) Subjects will be observed in the observation room for 30 minutes to ensure no adverse events, especially acute allergic reactions, and then followed up regularly as required.
c) Safety observations include assessment of adverse reactions/events and body temperature measurements. Body temperature should be taken daily 0-7 days after inoculation and when fever is suspected. Subjects will record safety observation data in the Diary Card within 28 days after vaccination of every dose. Subjects will be interviewed regularly by the investigator to verify and record adverse events and concomitant drugs/vaccines.
d) During Day 56 to Day 388, SAE ,AESI and the usage records of their concomitant drugs are only collected.
e) See "Visit Plan" for window period;

Visit plan:

Visit 1 - Day -3~ Day 0 - informed consent, **blood sampling, urine collection**, and screening of laboratory indexes (blood routine examination, blood biochemistry, and routine urine test); qualified subjects are enrolled and inoculated with the first dose of vaccine;

Visit 2- Day 3 (±1 day) after 1st dose - Verify safety observation, drug use and other vaccination records, and **collect blood and urine;**

Visit 3- Day 8 after 1st dose - Verify safety observation, drug use and other vaccination records**.**

Visit 4- Day 28 (+10 days) after 1st dose - Verify safety observation, drug use and other vaccination records, **collect blood and urine,** and inoculate the 2nd dose.

Visit 5- Day 3 (±1 day) after 2nd dose - Verify safety observation, drug use and other vaccination records, and **collect blood and urine;**

Visit 6- Day 8 after 2nd dose - Verify safety observation, drug use and other vaccination records**.**

Visit 7- Day 28 (+10 days) after 2nd dose - Verify safety observation, drug use and other vaccination records, and **sample blood.**

Visits 7~8 - Verify SAE and AESI observations, concomitant drug use records of SAE and AESI and other special cases.

Visit 8 - Day 180 (+30 days) after the 2nd dose - Verify SAE and AESI observations, concomitant drug use record and other special cases, and **sample blood.**

Visits 8~9 - Verify SAE and AESI observations, and concomitant drug use record of SAE and AESI and other special cases.

Visit 9 - Day 360 (+30 days) after the 2nd dose - Verify SAE and AESI observations, concomitant drug use record and other special cases, and **sample blood.**

10.1.2 Visit plan of Phase II

Table 12 Phase II Subject Visit Schedule

(Only for subjects with study numbers C101-C190, C281-C370 and C461-C520)

Follow-up		1	2	3	4	5	6	7	8	9	10	11
Follow-up period	D-14～D0	D0	D8[e]	D28[e]	D36[e]	D56[e]	D118[e]	D328[e, f]	D336[e,f]	D356[e,f]	D508[e,f]	D688[e,f]
Preliminary notice, subject recruitment	X											
Informed consent		X					X	X				
Demographic analysis		X										
General examination		X										
Urine pregnancy test (those with menophania, ≥14 years old)		X		X				X				
Screening based on inclusion/exclusion criteria[a]		X		X				X				
Neutralizing antibody test		X				X	X	X		X	X	X
Inoculation[b]		X		X				X				
Subjects record the safety observation results on their daily diary/contact cards[c].		X	X	X	X	X		X	X	X		
Monitoring of adverse reactions/events (including adverse events of grade 3 and above and SAE) [cd]		X	X	X	X	X	X	X	X	X	X	X
Usage records of concomitant drugs/vaccines[cd]		X	X	X	X	X	X	X	X	X	X	X

a) Screening shall be conducted based on inclusion/exclusion criteria before vaccination of every dose.

b) Subjects will be observed in the observation room for 30 minutes to ensure no adverse events, especially acute allergic reactions, and then followed up regularly as required.

c) Safety observations include assessment of adverse reactions/events and body temperature measurements. Body temperature should be taken daily 0-7 days after

inoculation and when fever is suspected. Subjects will record safety observation data in the Diary Card within 28 days after vaccination of every dose. Subjects will receive face to face visit regularly by the investigator to verify and record adverse events and concomitant drugs/vaccines.

d) During Day 56 to Day 328 and D356 to D688 , SAE ,AESI and the concomitant drugs use records of SAE and AESI are only collected.

e) See “Visit Plan” for window period;

f) Only subjects in the experimental group received visit 7 and visit 11, and subjects in the placebo group didn’t receive booster immunization and related visit.;

Visit plan:

Visit 1 - Day 0 - informed consent, enrollment of qualified subjects, **blood sampling**, and first dose of vaccination.

Visit 2- Day 8 after 1st dose - Verify safety observation, drug use and other vaccination records**.**

Visit 3- Day 28 (+10 days) after 1st dose - Verify safety observation, drug use and other vaccination records, and inoculate the 2nd dose.

Visit 4- Day 8 after 2nd dose - Verify safety observation, drug use and other vaccination records**.**

Visit 5- Day 28 (+10 days) after 2nd dose - Verify safety observation, drug use and other vaccination records, and **blood sampling.**

Visits 5 & 6 - Verify SAE and AESI observations, the concomitant drug use records of SAE and AESI and other special cases.

Visit 6 - Day 90 (+30 days) after the second dose or Day 180 (+30 days) after the second dose - Verify SAE and AESI observations, the concomitant drug use records of SAE and AESI and other special cases, and **blood sampling.**

Visits 6~7 - Verify SAE and AESI observations, the concomitant drug use records of SAE and AESI and other special cases.

Visit 7 - Day 300 (+30 days) after the 2nd dose - Verify SAE and AESI observations, the concomitant drug use records of SAE and AESI and other special cases, and **blood sampling,** vaccination of booster immunization.

Visit 8-Day 8 after booster immunization -Verify safety observation, the records of drug use and other vaccination.

Visit 9-Day 28(+10 days) after booster immunization- Verify safety observation, the records of drug use and other vaccination, **blood sampling.**

Visit 10-Day 180(+30 days) after booster immunization-Verify SAE and AESI observation, the concomitant drug use records of SAE and AESI and other special cases, **blood sampling.**

Visit 11- Day 360(+30 days) after booster immunization-Verify SAE and AESI observation, the concomitant drug use records of SAE and AESI and other special cases, **blood sampling.**

Table 13 Phase II Subject Visit Schedule

(Only for subjects with study numbers C191-C280, C371-C460 and C521-C580)

Follow-up		1	2	3	4	5	6	7	8	9	10	11
Follow-up period	D-14～D0	D0	D8[e]	D28[e]	D36[e]	D56[e]	D208[e]	D388[e, f]	D396[e,f]	D416[e,f]	D568[e,f]	D748[e,f]
Preliminary notice, subject recruitment	**X**											
Informed consent		**X**						**X**				
Demographic analysis		**X**										
General examination		**X**										
Urine pregnancy test (those with menophania≥14 years old)		**X**		**X**				**X**				
Screening based on inclusion/exclusion criteria[a]		**X**		**X**				**X**				
Neutralizing antibody test		**X**				**X**	**X**	**X**		**X**	**X**	**X**
Inoculation[b]		**X**		**X**				**X**				
Subjects record the safety observation results on their daily diary/contact cards[c].		**X**	**X**	**X**	**X**	**X**		**X**	**X**	**X**		
Monitoring of adverse reactions/events (including adverse events of grade 3 and above and SAE)[cd]		**X**	**X**	**X**	**X**	**X**	**X**	**X**	**X**	**X**	**X**	**X**
Use records of concomitant drugs/vaccines[cd]		**X**	**X**	**X**	**X**	**X**	**X**	**X**	**X**	**X**	**X**	**X**

a) Screening shall be conducted based on inclusion/exclusion criteria before vaccination of every dose.
b) Subjects will be observed in the observation room for 30 minutes to ensure no adverse events, especially acute allergic reactions, and then followed up regularly as required.
c) Safety observations include assessment of adverse reactions/events and body temperature measurements. Body temperature should be taken daily 0-7 days after inoculation and when fever is suspected. Subjects will record safety observation data in the Diary Card within 28 days after vaccination of every dose. Subjects will receive face-to-face visit regularly by the investigator to verify and record adverse events and concomitant drugs/vaccines.
d) During Day 56 to Day 388 and D416 to D748 , SAE,AESI and the concomitant drugs use records of SAE and AESI are only collected.
e) See “Visit Plan” for window period;
f) Only subjects in the experimental group received visit 7 and visit 11, and subjects in the placebo group didn’t receive booster immunization and related visit

Visit plan:

Visit 1 - Day 0 - informed consent, enrollment of qualified subjects, **blood sampling**, and vaccination of the first dose.

Visit 2- Day 8 after 1st dose - Verify safety observation, drug use and other vaccination records**.**

Visit 3- Day 28 (+10 days) after 1st dose - Verify safety observation, drug use and other vaccination records, and inoculate the 2nd dose.

Visit 4- Day 8 after 2nd dose - Verify safety observation, drug use and other vaccination records**.**

Visit 5- Day 28 (+10 days) after 2nd dose - Verify safety observation, drug use and other vaccination records, and **blood sampling.**

Visits 5 & 6 - Verify SAE and AESI observations, the concomitant drug use records of SAE and AESI and other special cases.

Visit 6 - Day 180 (+30 days) after the second dose after the second dose - Verify SAE and AESI observations, the concomitant drug use records of SAE and AESI and other special cases, and **blood sampling.**

Visits 6~7 - Verify SAE and AESI observations, the concomitant drug use records of SAE and AESI and other special cases.

Visit 7 - Day 360 (+30 days) after the 2nd dose - Verify SAE and AESI observations, the concomitant drug use records of SAE and AESI and other special cases, and **blood sampling,** vaccination of booster immunization.

Visit 8-Day 8 after booster immunization -Verify safety observation, the records of drug use and other vaccination, and **blood sampling**.

Visit 9-Day 28(+10 days) after booster immunization- Verify safety observation, the records of drug use and other vaccination, **blood sampling.**

Visit 10-Day 180(+30 days) after booster immunization-Verify SAE and AESI observation, the concomitant drug use records of SAE and AESI and other special cases, **blood sampling.**

Visit 11-Day 360(+30 days) after booster immunization-Verify SAE and AESI observation, the concomitant drug use records of SAE and AESI and other special cases, **blood sampling.**

10.2 Recruitment and Informed Consent

Recruitment notices will be issued to volunteers who meet the enrollment criteria. The informed consent should be explained to the volunteers and/or their guardians in detail. Under the condition of voluntary participation, the volunteers and/or their guardians and the study doctors sign the informed consent which is in duplicate, and the copy is reserved by the volunteers and/or their guardians.

10.3 Screening and Random Enrollment

The subjects who meet the inclusion criteria and don't meet the exclusion criteria are eligible to be enrolled into the study. The screening number is CS+ screening sequence number, such as "CS0001". The enrolled subject will be assigned a study number in the order of enrollment. In the phase Ⅰ clinical trial and the phase Ⅱ clinical trial, study numbers of subjects are C001-C072 and C101-C580 respectively.

10.4 Vaccination

According to the study number of the subject, the vaccinator takes out the corresponding vaccine labelled with the same number and opens the package box, checks the information of label on the syringe, label in the package box, and label on the outer surface of package box, the vaccination should be carried outs with the condition that information on the three labels are confirmed consistency. After vaccination, the label in the backage box should be removed and pasted on the specific location of the original logbook, simultaneously, the vaccination information should be recorded in the original logbook.

See "8.3 Study plan" for immunization schedules.

10.5 Safety Follow-up and Observation

Subjects will be observed for 30 minutes on site after each dose of vaccination. Diary cards and contact cards are distributed to subjects to record the adverse events within 0~7 days and 8~28 days respectively. Doctors should explain the judgment, measurement, recording, precautions and reporting method of adverse events. Systematic observation is carried out within 7 days after vaccination. Subjects are required to closely observe their own symptoms and vital signs and fill in the diary card every day. The investigators verify the adverse events on the 8th days after vaccination through face-to-face interviews on all subjects (those who do not face-to-face interviews are conducted by telephone), collect diary cards and distribute contact cards to record the adverse events within 8~28 days. The investigators verify the adverse events on the 28th days and collect contact cards.

The subjects are informed to record the adverse events at any time. Acute allergic reactions, severity level 3 and above adverse events and SAE should be reported to the investigators timely. After the investigators are informed, they should conduct investigation, verification and follow-up until the adverse event is solved, and finally complete the detailed investigation and follow-up records, which should include the following contents:

- Description of adverse events
- Start time and end time of adverse events
- Severity of adverse events
- Correlation with vaccination

- Laboratory test results
- Processing measures

If subjects develop acute allergic reaction and grade 3 or above adverse events after vaccination, treatment should be provided in time to relieve the pain of the subjects as soon as possible. Medication and medical treatment at each follow-up shall be recorded in detail.

10.6 Sampling

- **Sampling plan and sample number**

Sampling before/after immunization should be conducted according to "10.1 Visit Plan" for subjects. See the sampling plan in Table 14, Table15 and Table16. During screening, samples are numbered as "screening No. + serial number of sampling" and the samples of enrolled subjects are numbered as "study number + serial number of sampling".

Table 14 Sampling Plan for Phase I Clinical Trial

Sample type	Blood sampling time	D-3～D0	D3[e]	D28[e]	D31[e]	D56[e]	D118[e,]	D208[e]	D388 [e]
Venous blood (ml)	Blood routine	2.0~2.5	2.0~2.5	2.0~2.5	2.0~2.5	-	-	-	-
	Blood biochemistry	3.0~3.5	3.0~3.5	3.0~3.5	3.0~3.5	-	-	-	-
	Serum antibody	3.5~4.0	-	3.5~4.0	-	3.5~4.0	-	2.5~3.5	2.5~3.5
	**Total**	**8.5~10.0**	**5.0~6.0**	**8.5~10.0**	**5.0~6.0**	**3.5~4.0**	**-**	**2.5~3.5**	**2.5~3.5**
Urine (ml)	Urine routine test	5~10	5~10	5~10	5~10	-	-	-	-
	Urine pregnancy test (those with menophania, ≥14 years old)	5~10	-	5~10	-	-	-	-	-

Table 15 Sampling Plan for Phase Ⅱ Clinical Trial

(Only for subjects with study numbers C101-C190, C281-C370 and C461-C520)

Sample type	Blood sampling time	D0	D28[e]	D56[e]	D118[e]	D328[e]	D356[e,f]	D508[e,f]	D688[e,f]
Venous blood (ml)	Serum antibody	3.5~4.0	-	3.5~4.0	2.0~2.5	2.0~2.5	2.0~2.5	2.0~2.5	2.0~2.5
Urine (ml)	Urine pregnancy test (those with menophania, ≥ 14 years old)	5~10	5~10	-	-	5~10	-	-	-

[e] See "10.1 Visit Plan" for window period;

f Only subjects in the experimental group received blood sampling for 328 days and subsequent days, and subjects in the placebo group didn't receive booster immunization and related visits.;

Table 16 Sampling Plan for Phase Ⅱ Clinical Trial

(Only for subjects with study numbers C191-C280, C371-C460 and C521-C580)

Sample type	Blood sampling time	D0	D28[e]	D56[e]	D208[e]	D388[e,f]	D416[e,f]	D568[e,f]	D748[e,f]
Venous blood (ml)	Serum antibody	3.5~4.0		3.5~4.0	2.0~2.5	2.0~2.5	2.0~2.5	2.0~2.5	2.0~2.5
Urine (ml)	Urine pregnancy test (those with menophania, ≥ 14 years old)	5~10	5~10	-	-	5~10	-	-	-

[e] See "10.1 Visit Plan" for window period;

f Only subjects in the experimental group received blood sampling for 328 days and subsequent days, and subjects in the placebo group didn't receive booster immunization and related visits

- **Sample management**

All samples collected on site should be sent to the laboratory in time to complete the handover with laboratory personnel. The laboratory personnel separate the serum from the blood sample for serum antibody detection, and the serum separate before immunization, Day 28 (only Phase I) after the first dose of immunization and the second dose of immunization is filled into three tubes (no less than 0.5 ml for single serum tube A, for neutralizing antibody detection; backup serum in tubes B and C); the serum separated 3 months after immunization of the second dose and later blood sampling are filled into two tubes (no less than 0.5 ml in single serum tube A, for neutralizing antibody detection; backup serum for tube B). After separation, serum should be kept below -20 ºC. The handover, serum separation and preservation of samples shall be recorded. For all submitted samples, specimen submission record should be made and temperature control record during submission should be kept.

10.7 Safety Evaluation

10.7.1 Safety Observation Indexes

✧ **Solicited local adverse events:**

(1) pain, (2) induration, (3) swelling, (4) vaccinal areola, (5) rash, (6) pruritus.

✧ **Solicited systemic adverse events (including vital signs):**

(1) fever (axillary temperature), (2) acute allergic reaction, (3) abnormal skin and mucosa, (4) diarrhea, (5) anorexia, (6) vomiting, (7) nausea, (8) muscle pain, (9) headache, (10) cough, (11) fatigue.

✧ **Adverse Events of Special Interest :**

These include Bell's palsy, sudden deafness/hearing loss, brachial plexus/multiple neuritis, Guillain-Barre syndrome, thrombocytopenic purpura, allergic purpura, thrombosis, myelitis, myocarditis, immune thrombocytopenia, convulsion, and multisystem inflammatory syndrome in children (MISC).

✧ **Phase I laboratory test:**

(1) Blood routine examination: white blood count, hemoglobin and platelet count;

(2) Blood biochemistry: alanine aminotransferase (ALT), aspartate aminotransferase (AST), total bilirubin (TBIL), creatinine (CR) and blood glucose.

(3）Routine urine: urine protein (PRO).

10.7.2 Definition of adverse event/reaction

The safety of vaccines will be evaluated according to the scope, intensity, and severity of the local adverse events, systemic adverse events as well as abnormality of vital signs, and the correlation of the above events with vaccination. All adverse medical events occurring during the trial (since signing

of the informed consent form) should be collected and recorded.

Adverse events (AE): Adverse medical events that occur after vaccination, which can be manifested as symptoms and signs, diseases or laboratory abnormalities, but are not necessarily causally related to the trial vaccine.

Adverse reactions: Any harmful or unexpected reaction that may be related to the trial vaccine in clinical trials. There is at least one reasonable possibility that the causal relationship between experimental vaccine and adverse events can not be excluded.

Serious adverse event (SAE): it refers to the events during the clinical trial that need hospitalization treatment, prolong hospitalization time, disability, affect working ability, endanger life or death, cause congenital malformation, etc.

Suspicious and unexpected serious adverse reactions: refers to the suspicious and unexpected serious adverse reactions with the severity of clinical manifestations exceeding the existing data and information, including the trial drug researcher's manual, the instruction manual of marketed drugs or the summary of product characteristics.

Solicited/unsolicited adverse events: In this trial, the solicitation period is 0-7 days after each dose of vaccination, and the non-solicitation period is 8-28 days. The solicited adverse events refer to the solicited symptoms occur within the solicitation period, and the unsolicited adverse events refer to the unsolicited symptoms occur within the solicitation period, and any symptoms occur within the non-solicitation period.

10.7.3 Outcome of Adverse Events

The outcomes of adverse events include: (1) recovery, (2) not recovered, (3) recovered with sequela, (4) death, (5) loss to follow-up/unknown.

10.7.4 Correlation Between Adverse Events and Vaccines

Investigators should try their best to explain AE and assess the possible causal links, i.e., the causal link with the inoculation of the research vaccine and the superseding causes (such as the medical history and combined treatment of underlying diseases). It is applicable to all AEs, including the severe and non-severe ones.

The causal link assessment will be determined by the degree of the reasonable explanation of events obtained in the following one or more aspects:

As for the preparations of such kind, reactions of similar properties had been observed previously;

For preparations of similar kinds, similar events had been reported on literature;

From the perspective of time, the events occur with the inoculation of the research vaccine and re-occur after the re-inoculation of the research vaccine.

The causal links of AE shall be assessed by investigators according to the following questions, and whether there are reasonable probabilities that the AE is caused by vaccination:

a. **Certainly related:** there is evidence of vaccination of experimental vaccine; the time sequence of adverse events and vaccination is reasonable; the occurrence of adverse events is more reasonable than other reasons; repeated vaccination will induce the adverse events; adverse events are consistent with previous knowledge of this or this kind of vaccine.

b. **Probably related**: there is evidence of vaccination of experimental vaccine; the time sequence of adverse events and vaccination is reasonable. It is more reasonable to explain adverse events by experimental vaccine than by other reasons.
c. **Possibly related**: there is evidence of vaccination of experimental vaccine; the time sequence of adverse events and vaccination is reasonable. The causes of adverse events can not be excluded from the experimental vaccine, but also may be caused by other reasons.
d. **Possibly unrelated**: there is evidence of vaccination of experimental vaccine; adverse events are more likely to be caused by other reasons; repeated vaccination will possibly not induce the adverse events.
e. **Definitely unrelated**: the subjects do not use the experimental vaccine; or the occurrence of adverse events was illogical with the time sequence of vaccination; or there were other significant reasons that could lead to adverse events.

10.7.5 Handling of Adverse Events

Reactions below grade 2 such as vaccinal areola, swelling, pain, or (and) fever and general malaise after vaccination can generally disappear spontaneously without special treatment.

The investigator should make investigation and medical follow-up on the adverse reactions/events of grade 3 and above that occur in subjects from the start of primary immunization to 28 days after immunization and within 28 days of booster immunization including medical history, physical examination and necessary laboratory examination, treatment and tracking until the event is solved, and detailed investigation records should be completed. The investigation records shall include symptoms, signs and diagnosis.

In the event of a SAE, the Investigator shall promptly take necessary actions and report it within 24 hours. During test observation, the subjects who develop fever with cough and other respiratory symptoms shall, when necessary, immediately go to the designated hospitals, have the throat swabs/sputum and anal swabs collected, and be subjected to imaging examinations such as CT to determine if the disease is caused by COVID-19 infection. In case of COVID-19 infection, it shall be treated according to SAE, and the occurrence of ADE shall be especially analyzed.

10.7.6 Report on Serious Adverse Events

(1) **Reporting procedures for investigators**
(1) The responsible organization establishes an emergency plan for SAE. After the investigator becomes aware of the SAE, appropriate action should be taken for the subject and documented immediately. The investigator must submit an initial report of the Serious Adverse Event Report Form by fax, E-mail, or personal delivery immediately to the sponsor, principal investigator, and clinical research associate after learning of the SAE, whether the SAE related to the experimental vaccine or not. Follow-up Serious Adverse Event Report Form should be submitted periodically until the end of the event. If death is involved, the investigator shall report to sponsor, the Independent Ethics Committee, principal investigator, and clinical research associate, as well as provide autopsy reports and final medical reports. Complete information, including description of adverse reactions/events, time and type of onset, duration, severity, causality to vaccination, outcome, treatment (symptomatic treatment) and other relevant clinical and laboratory data, is reported in a written report on the Serious Adverse Event Report Form.

When receipt of the report of a serious adverse event/reactions, the investigator and the sponsor should

decide subjects whether to continue the study or terminate the study early, considering about the duration, scope, severity, outcome and the subjects' wishes of the adverse event.

(2) **Reporting procedures for sponsor**

During clinical drug trial, the sponsor should judge the suspicious and unexpected serious adverse reactions (SUSAR) or the adverse reactions definitely related to the test drug according to the Standards and Procedures for Rapid Reporting of Safety Data During Clinical Drug Trial and quickly give response with a case-by-case safety report.

For suspicious and unexpected serious adverse reactions (SUSAR) resulting in death or threatening the life, the sponsor shall soon report them upon being first informed within 7 nature days and report related follow-up information in next 8 nature days (The day on which the applicant is first informed is Day 0). For SUSAR not resulting in death or threatening the life, the sponsor shall soon report them upon being first informed within 15 nature days. For information on other potentially serious safety risks, the sponsor should also report to the national drug evaluation agency as soon as possible, while a medical and scientific judgment should be made for each case. After the initial report, the sponsor shall continue to track SAE and report related new information or change information to the previous report in the form of follow-up reports within 15 days after receiving new information. The sponsor is not allowed to change the investigator's judgment of the correlation between SAE and the vaccine. In case of disagreement between the sponsor and the investigator, the opinions of the sponsor and the investigator should be showed in detail in the report, and reported according to the higher management requirements.

In exceptional cases, the investigator and sponsor should timely provide SAE related information and safety reports as required by regulatory authorities and the independent ethics committee.

(3) The contacts and contact information of the Sponsor, the Independent Ethics Committee and Hebei Medical Products Administration are as follows:

Sinovac Life Sciences Co., Ltd.: 24h contact: Wang Jiayi; Tel. 18518337983; E-mail: wangjy1755@sinovac.com; fax: 010-82890408.

Contact of Hebei Provincial Center for Disease Control and Prevention: Doctor Yong; Tel.: 0311-86573167; fax: 0311-86573167

10.7.7 Safety Evaluation Criteria

Solicited local adverse events, systemic adverse events and vital signs: the grading of solicited adverse events mainly refers to the Guidelines of the National Medical Products Administration for Adverse Event Classification Standards for Clinical Trials of Preventive Vaccines (2019)[15], as shown in the following table. Solicited adverse events and non-solicited adverse events of the same symptom are graded according to the following criteria.

Table 17 Grading of (Local) Adverse Events at Inoculation Site

	Grade 1	Grade 2	Grade 3	Grade 4
Pain	Having no or marginal effect on limb activity	Having an effect on limb activity	Having an effect on daily life	Loss of basic living skills or hospitalization
Induration * #, swelling #				

＞ 14 years old	Diameter 2.5-＜5 cm or area 6.25-＜25 cm^2 and having no or marginal effect on daily life	Diameter 5-＜10 cm or area 25-＜100 cm^2 or having an effect on daily life	Diameter ≥10 cm or area ≥100 cm^2 or fester or secondary infection or phlebitis or sterile abscesses or wound drainage or having serious effect on daily life	Abscess, exfoliative dermatitis, dermal or deep tissue necrosis
≤14 years old	Diameter < 2.5 cm	Diameter ≥2.5cm, and area < 50% of the inoculated limb (the limb where the inoculation is received in terms of anatomy, such as upper arm or thigh)	Area ≥ 50% of the inoculated limb <u>or</u> fester <u>or</u> secondary infection <u>or</u> phlebitis <u>or</u> wound drainage	Abscess, exfoliative dermatitis, dermal or deep tissue necrosis
Vaccinal areola #, rash* #				
＞ 14 years old	Diameter 2.5-＜5 cm or area 6.25-＜25 cm^2 and having no or marginal effect on daily life	Diameter 5-＜10 cm or area 25-＜100 cm^2 or having an effect on daily life	Diameter ≥10 cm or area ≥100 cm^2 or fester or secondary infection or phlebitis or sterile abscesses or wound drainage or having serious effect on daily life	Abscess, exfoliative dermatitis, dermal or deep tissue necrosis
≤14 years old	Diameter < 2.5 cm	Diameter ≥2.5cm, and area < 50% of the inoculated limb (the limb where the inoculation is received in terms of anatomy, such as upper arm or thigh)	Area ≥ 50% of the inoculated limb or fester or secondary infection or phlebitis or wound drainage	Abscess, exfoliative dermatitis, dermal or deep tissue necrosis
Pruritus	Pruritus at inoculation site, mitigated spontaneously or within 48 hours after treatment	Pruritus at inoculation site, not mitigated within 48 hours after treatment	Having an effect on daily life	NA

*Induration and rash: In addition to the grading and evaluation by measuring the diameter directly, the change of measurements should also be recorded.

#Induration and swelling, rash and vaccinal areola: The maximum measured diameter or area should be used; The grading and evaluation should be based on the function grade and actual measurements and indicators with a higher grade should be chosen.

Table 18 Grading of (Systemic) Adverse Events and Vital Signs Not at Inoculation Site

	Grade 1	Grade 2	Grade 3	Grade 4
Acute allergic reaction*	Local urticaria (blister), no treatment required	Local urticaria, requiring for treatment or mild angioedema, no treatment required	Extensive urticaria or angioedema requiring for treatment or mild bronchospasm	Allergic shock or life-threatening bronchospasm or laryngeal edema
Abnormal skin & mucous	Erythema/pruritus/color change	Diffuse rash/maculopapule/xerosis cutis/desquamation	Herpes zoster/exudation/desquamation/ulceration	Exfoliative dermatitis (involving mucosa) or erythema multiforme or suspected Stevens-Johnsons syndrome
Diarrhoea	Mild or transient, 3 or 4 times per day, abnormal poop or mild diarrhea lasting less than a week	Moderate or persistent, 5 to 7 times per day, abnormal poop, or diarrhea lasting over 1 week	Over 7 times per day, abnormal poop, or bloody diarrhea, orthostatic hypotension, electrolyte imbalance, venous transfusion >2L indicated	Hypotensive shock, hospitalization indicated

	Grade 1	Grade 2	Grade 3	Grade 4
Anorexia	Loss of appetite, but normal food intake	Loss of appetite, decreased food intake, but no significant weight loss	Loss of appetite, and significant weight loss	Need for intervention (such as tube feeding and parenteral nutrition)
Vomiting	1 - 2 times /24 hours and daily activities not affected	3 to 5 times /24 hours or limited activity	Over 6 times within 24 hours or requiring intravenous infusion	Hospitalization or other nutrition channels indicated due to hypotensive shock
Nausea	Transient (<24 hours) or intermittent, and basically normal food intake	Persistent nausea leads to reduced food intake (24-48 hours)	Persistent nausea leads to almost no food intake (>48 hours) or requiring intravenous infusion	Life-threatening (such as hypotensive shock)
Muscular pain (not at the inoculation site)	Daily activities not affected	Daily activities marginally affected	Severe muscle pain, and daily activities severely affected	Urgent intervention or hospitalization indicated
Headache	Daily activities not affected, and treatment not required	Transient, daily activities marginally affected, treatment or intervention probably required	Daily activities severely affected, treatment or intervention required	Refractory, urgent intervention or hospitalization required
Cough	Transient, no treatment required	Continuous cough which can be treated effectively	Paroxysmal cough which cannot be controlled by treatment	Urgent intervention or hospitalization indicated
Fatigue and weakness	Hypoergia <48 hours, no impact on activity	Hypoergia for 20% to 50% >48 hours, with slight impact on activity	Hypoergia for >50%, with heavy impact on activity	Incapable of taking care of oneself, and emergency treatment or hospitalization
**Vital signs** - fever, axillary temperature (°C)				
>14 years old	37.3～<38.0	38.0～<38.5	≥38.5	≥39.5, lasting over 3 days
≤14 years old	37.5～<38.0	38.0～<39.5	≥39.5	≥39.5, lasting over 5 days

The sign * indicates type I hypersensitivity

Laboratory indexes: The first step is to determine the clinical significance. When there are "abnormality and clinical significance", the grading mainly refers to the Guidelines of the National Medical Products Administration for *Adverse Event Classification Standards for Clinical Trials of Preventive Vaccines* (2019)[15], the *Guidelines for Adverse Event Classification Standards for Clinical Trials of Preventive Vaccines* (2005) (Only Creatinine)[16] and the Grading Criteria of the National Institute of Allergy and Infectious Diseases (NIAID) under the National Institutes of Health (NIH) for Clinical Assessment (Only Platelets, aged 13-17)[17], as shown in the following table:

Table 19 Grading of Blood Routine Indexes

Indicators/grading	Grade 1	Grade 2	Grade 3	Grade 4
Leukocyte increase (WBC, 10^9/L)	11～<13	13～<15	15～<30	≥30
Leukocyte decrease (WBC, 10^9/L)	2.000～2.499	1.500～1.999	1.000～1.499	<1.000
Low hemoglobin (g/dL) - male aged 13-17 years old	10.0～10.9	9.0～<10.0	7.0～<9.0	<7.0

Indicators/grading	Grade 1	Grade 2	Grade 3	Grade 4
Low hemoglobin (g/dL) - female aged 13-17 years old	9.5～10.4	8.5～<9.5	6.5～<8.5	<6.5
Low hemoglobin (g/dL)- 3-12 years old	9.5～10.4	8.5～<9.5	6.5～<8.5	<6.5
Platelet (10^9/L)- (13-17 years old)	75-99.999	50-74.999	20-49.999	<20
Platelet (10^9/L) -(3~12 years old)	NA	50-75	25-49.999	<25

Table 20 Grading of Blood Biochemical Indexes

Indicators/grading	Grade 1	Grade 2	Grade 3	Grade 4
Liver function (ALT,AST)	1.25～<2.5 ×ULN	2.5～<5.0×ULN	5.0～<10×ULN	≥10×ULN
Increase of total bilirubin (mg/dL; μmol/L)	1.1～<1.6×ULN	1.6～<2.6×ULN	2.6～5.0×ULN	≥5.0×ULN
Creatinine (CR)	1.1～1.5×ULN	1.6～3.0×ULN	3.1～6×ULN	>6×ULN
Hyperglycemia (fasting) (Glu, mmol/L)	6.11～<6.95	6.95～<13.89	13.89～<27.75	≥27.75

Note: The ULN refers to the upper limit of normal; blood glucose shall be measured on an empty stomach.

Table 21 Grading of Routine Urine Test Indexes

Indicators/grading	Grade 1	Grade 2	Grade 3	Grade 4
Urine protein (PRO) (Urine test strip)	1+	2+	3+ orhigher	NA

Adverse events not included in above grading table should be graded and evaluated according to the following standards:

Grade 1 mild: short time (<48h) or slight discomfort; daily activities not affected, and treatment not required;

Grade 2 moderate: mild or moderate limited activity, medical attention probably required, treatment not required or mild treatment required;

Grade 3 severe: obvious limited activity, treatment required, hospitalization probably required;

Grade 4 critical: probably deadly, severe limited activity, monitoring and treatment required.

Grade 5: death

10.8 Concomitant Medication and Vaccination

10.8.1 Concomitant Medication

- If any adverse event (AE) occurs during the trial, the drug therapy and medical treatment should be allowed if necessary.
- In case of severe allergic reaction or life threatening events, first aid measures should be taken immediately.
- The investigator should record any concomitant medication information, including name, dosage form, dosage, and duration of use.

10.8.2 Concomitant Vaccination

- Other vaccines can be administered at least 7 days after the test vaccine is administered.
- During the trial, subjects can be vaccinated with such vaccines as rabies vaccine and tetanus vaccine in case of emergency.
- Detailed information should be recorded, including the name of the vaccine, the use of the vaccine and the time of vaccination if concomitant vaccination.

10.9 Immunogenicity evaluation

Blood samples collected at different points-in-time shall be subject to neutralizing antibody test, and the seroconversion rate, seropositive rate, GMT and GMI shall be calculated.

10.9.1 Evaluation Standards

The evaluation standards for positive serum neutralizing antibody are as follows:

- If antibody titer≥1:8, it is positive.

The evaluation standards for seroconversion of serum neutralizing antibody are as follows:

- The seroconversion is defined as a post-vaccination Nab titer ≥1:8 if seronegative (<1:8) at beseline, or a 4 fold increase of Nab titer if seropositive (≥1:8) at baseline.

10.9.2 Laboratory Test Methods

Neutralizing antibody test: micro virus neutralization test (MVNT);

10.10 Data management

10.10.1 Original Data

The original data should include the informed consent form, diary cards and original record books. The following basic data should be recorded.

- Trial name, subject number
- Demographic data
- Inclusion/exclusion criteria
- Vaccination records
- Follow-up date and date of discontinuation of the trial discontinuation date of the subject
- Adverse events/reactions and the corresponding treatment and outcome
- Concomitant medical treatment and other vaccinations

All data should have original records, which should be properly kept by investigators in a dedicated space. The original data should be archived in the study site, which is the true and complete evidence for the participation of the subjects in the clinical trial.

The investigators should carefully, accurately and timely make the original records. All the collected original data should be recorded on the same day with that of the data collection. Additionally, the raw data should be recorded using the black sign pen, and the mistake record should be crossed out with the correct content being written beside it along with the signature of the modifier, instead of be altered directly.

10.10.2 Case Report Form (CRF)

In this trial, “Electronic Data Capture (EDC)” is used to establish the electronic CRF. As an important

component of the clinical trials and research reports, the electronic CRF is used to record clinical trial data. Information should be inputted with standard language according to the EDC instructions and CRF filling instructions.

The data on the electronic CRF should be derived from and consistent with the original data. The input, verification, modification, cleanup and quality control of any electronic CRF data will be recorded in the EDC system. Upon completion of the data cleanup, the investigator should confirm the data in each electronic CRF and create an electronic signature for each electronic CRF.

Only the investigator and authorized staff will be allowed to access the EDC system during the trial.

10.10.3 Data Locking

After input, verification and cleanup of all data, the final data verification is carried out. According to the evaluation criteria, the analyzed population should be determined, and the situations that deviate from the schedule as well as their impact on data group analysis should be confirmed. Then, the database should be locked.

10.10.4 Privacy Protection for Subjects and Data Utilization Range

Any information regarding the identity of the subject will be confidential and the name will not appear in any publication or report of the study. Study records will be made available to the sponsor's representatives in the presence of the investigator for the purpose of medical data collection. In addition, the CRA and inspector of this study, and the representatives of Independent Ethics Committee of Hebei Provincial Center for Disease Control and Prevention and National Medical Products Administration (NMPA) can review the original research materials of the subjects involved this study to verify the accuracy of the data collected in this study. The original data obtained in this study will only be used for publication of papers or results related to this project.

10.11 Statistical analysis

10.11.1 Analysis Set

10.11.1.1 Safety Set, SS

(1) Safety Set of Primary immunization(SS)
All subjects who have completed at least one dose of vaccination after randomization are included in the safety set. Among them, subjects receiving wrong vaccination in safety evaluation should be statistically analyzed based on actual vaccine groups according to ASaT (All Subjects As Treated) **principle.**

The safety dataset is divided into total safety dataset, first dose safety set and second dose safety set. The total safety set includes subjects administrated with at least one dose of vaccination; the first dose safety set includes subjects administrated with the first dose of vaccine; the second dose safety set includes subjects administrated with the second dose of vaccine.

(2) Safety Set for Booster immunization(bSS)
All randomized subjects who receive the booster dose. Subjects who are vaccinated with the wrong vaccine would be transferred to the group of actually administered vaccine according to the ASaT principle (All Subjects as Treated), for the safety evaluation.

10.11.1.2 Immunogenicity Set

(1) Full Analysis Set of Primary Immunization, FAS

A population defined according to the principle of intent-to-treat (ITT), including all the subjects who have been randomized, have received at least one dose of vaccination, have finished blood collection before/after vaccination for at least one time with the corresponding antibody assay results provided. The subjects who are vaccinated with the wrong vaccine will be kept in the originally assigned group according to the ITT principle, for the immunogenicity evaluation.

(2) Per-Protocol Set of Primary Immunization, PPS

It is a subset of FAS, including all subjects who will follow the inclusion/exclusion criteria, are randomized into groups, finish vaccination of primary immunization within the time window period according to the protocol, have the blood collected before and after immunization, and have effective antibody titer values. Among them, subjects who meet the following conditions cannot enter PPS:

1） Receive the wrong vaccine;
2） Use vaccines or drugs prohibited by the protocol:
    ①Other research or unregistered products (drugs or vaccines) that are not research vaccine
    ② Long-term use (lasting for more than 14 days) of immunosuppressants or other immunomodulatory drugs (inhaled or topical steroids are allowed);
    ③Immunoglobulin and/or blood preparations;
3） Newly diagnosed autoimmune diseases, including human immunodeficiency virus (HIV) infection;
4） Other situation under which the evaluation on vaccine immunogenicity will be affected.

(3) Immune Persistence Set 3 of Primary Immunization, IPS-3

All subjects who complete the vaccination of primary immunization according to the Day 0,28 immunization schedule, have blood sampling 3 months after primary immunization with effective antibody titer values are included.

(4) Immune Persistence Set 6 of Primary Immunization, IPS-6

All subjects who complete the vaccination of primary immunization according to the Day 0,28 immunization schedule, have blood sampling 6 months after the primary immunization with effective antibody titer values are included.

(5) Immune Persistence Set 10 for Primary Immunization, IPS-10

All subjects who complete the vaccination of primary immunization according to the Day 0,28 immunization schedule, have blood sampling 10 months after the primary immunization with effective antibody titer values are included.

(6) Immune Persistence Set 12 for Primary Immunization, IPS-12

All subjects who complete the vaccination of primary immunization according to the Day 0,28 immunization schedule, have blood sampling 12 months after the primary immunization with effective antibody titer values are included.

(7) Full Analysis Set for Booster Immunization, bFAS

A population defined according to the principle of intent analysis (ITT), including all the subjects who have been randomized, completed primary immunization, entered the booster immunization process, and have received the booster dose vaccination, and have finished blood collection before booster dose vaccination with the corresponding antibody assay results provided. The subjects who are vaccinated with the wrong vaccine will be kept in the originally assigned group according to the ITT principle, for the immunogenicity evaluation.

(8) Per Protocol Set for Booster Immunization, bPPS

A subset of bFAS, including all the subjects who meet the inclusion criteria and don't meet the exclusion criteria, have been randomized, have finished the 1st and 2nd dose vaccination and the booster dose after the second dose within the protocol required time window, and have finished the blood collection before booster immunization and 28 days after booster dose vaccination with the corresponding antibody assay results provided. Subjects who meet the following conditions are not allowed to enter bPPS:

- Subjects vaccinated vaccine with incorrect number;
- Subjects who vaccinated for booster immunization and sampled blood 28 days after booster immunization out of time window
- Use of vaccines or drugs prohibited by the protocol:

①Other investigational or unregistered products (drugs or vaccines) of non-research vaccine.② Long-term use (lasting more than 14 days) immunosuppressants or other immunomodulatory drugs (inhaled or topical steroids are permitted)

③Immunoglobulin and/or blood products.

- Newly diagnosed autoimmune diseases, including human immunodeficiency virus (HIV) infection;
- Other conditions affecting evaluation of vaccine immunogenicity at 28 days after booster immunization.

(9) Immune Persistence Set of Booster Immunization, 6 months after booster dose, bIPS-6

All subjects who have completed primary immunization according to the 0, 28-day immunization schedule, and will receive booster immunization 10 months or 12 months after completion of primary immunization, and will collect blood at 6 months after completion of booster immunization with effective antibody titer value were included.

(10) Immune Persistence Set of Booster Immunization, 12 months after booster dose, bIPS-12

All subjects who have completed primary immunization according to the 0, 28-day immunization schedule, and will receive booster immunization 10 months or 12 months after completion of primary immunization, and will collect blood at 12 months after completion of booster immunization with effective antibody titer value were included.

10.11.2 Statistical Analysis Method

10.11.2.1 General principles

The measurement data are statistically described with average, standard deviation, median, maximum and minimum; the enumeration data or ranked data are presented by frequency and relative frequency.

All statistical analysis will be completed with the help of the statistical software SAS 9.4.

10.11.2.2 Characteristics of Subjects

The number of subjects who are screened and enrolled into each group and complete the test and the number of subjects in each analysis set are summarized; and the reasons for subjects' drop-out are analyzed. The list of subjects who fail in screening, the list of withdrawal subjects and the list of subjects who do not enter each analysis set are listed respectively.

10.11.2.3 Immunogenicity evaluation

The seroconversion rate and seropositive rate of neutralizing antibody after immunization in medium-dose group, high-dose group and placebo group are calculated respectively. The bilateral 95% confidence interval is calculated by Clopper-Pearson. Chi-square test/Fisher exact probability test is used to statistically test the differences between groups.

The GMT and GMI of neutralizing antibody after immunization in the test group and control group shall be calculated by geometric mean and 95% confidence interval, and the Group t test by logarithmic conversion shall be used to statistically test the differences between groups.

10.11.2.4 Safety Evaluation

Adverse events are medically coded by MedDRA. This study mainly analyzes the adverse events after vaccination, and the adverse events before vaccination will be listed.

The number of episodes, number of involved subjects, as well as the incidence rate of overall AEs, the vaccine-related AEs, and the vaccine-unrelated AEs in all the groups should be calculated separately, and the differences between groups will be statistically tested using the Fisher exact probability method. The severity, dose distribution and time distribution of general AEs as well as the vaccine-related AEs should be statistically analyzed. The list of the vaccine-related AEs, vaccine-unrelated AEs should be made separately. The AEs after each dose should be statistically analyzed based on the safety set of each dose respectively.

The number of epidoses, number of involved subjects, as well as the incidence rate of overall SAEs, the vaccine-related SAEs, and the vaccine-unrelated SAEs in all groups should be calculated separately, and the differences between groups will be statistically tested using the Fisher exact probability method. The list of the SAEs should be made.

10.11.2.5 Processing of missing data

In the statistical analysis of the full analysis set, for those with missing serum test results after immunization, the method of last observation carried forward (LOCF) is used to fill the data. For those with missing serum results before immunization but after immunization, the maximum value of the serum antibody before immunization is used to fill the value of antibody before immunization of all subjects and the corresponding immunogenicity endpoint is further derived and calculated. Missing data in exploratory endpoints and safety endpoints are not processed in this trial.

11 Clinical Trial Monitoring

11.1 Sponsor's Responsibility

The sponsor should execute and maintain the quality assurance and control system and prepare quality management documents to make sure that the test is executed according to regulations. Meanwhile,

data, record and report should meet the requirements of GCP and other regulations.

11.2 Investigator's Responsibility

The main investigator should manage and clearly divide the roles of all participants in the clinical trial. The investigator should keep the subject's individual data secret. The document provided to the sponsor should be identified only with the subject identification code and subject number. The investigator keeps a list of subjects' identifications in the investigator's file. In accordance with GCP principle, each subject's original data is allowed to be monitored, audited, and reviewed.

11.3 Personnel Training

Before the test, the participants shall be trained. The training shall include: GCP, clinical trial protocol, standard operating procedure (SOP), etc. If new monitors or investigators participate in the trial, they should be trained separately. Retraining can be conducted if the sponsor or principal investigator deems it necessary. Records should be made for each training.

11.4 Compliance Guaranteeing of Subjects

According to the clinical trial protocol, a concise and well-organized volunteer recruitment letter and an informed consent form are prepared.

Responsible physicians should be told to communicate with volunteers in plain language so as to make subjects fully informed.

Subjects are screened strictly according to the criteria for inclusion and exclusion.

Follow-up personnel should have a high sense of responsibility and professionalism. Training is required to improve their communication skills and affinity. In the process of safety follow-up, measures should be taken to ensure effective contact between subjects and investigators and timely deal with adverse reactions found. Subjects should be provided with relevant health consultation.

11.5 Management of Test Vaccine

11.5.1 Definition and Treatment of Cold Chain Damage

Once the temperature of refrigerators stored with vaccine is <2°C or >8°C, it shall be recorded as cold chain damage, and the investigator shall handle it as per the relevant operating procedures as soon as possible, report to the Sponsor in a timely manner, and decide whether to discontinue or continue using the vaccine according to written/e-mail reply from the Sponsor.

11.5.2 Acceptance of Test Vaccine

The sponsor sends the vaccines used in clinical trial to the test site. The investigator must sign the vaccine acceptance receipt which should briefly show information about the received vaccine (completeness of the package and normal indication of the cold chain system).

When the investigator finds that the vaccine package is damaged, that the vaccine goes bad or that there is any blocky substance that cannot be shaken, the vaccine cannot be used and should be returned to the sponsor. If the cold chain system is damaged during transportation and storage or the vaccine is frozen, the vaccine cannot be used, and should be stored separately, marked with "×" on the outer package, managed by the designated person, and returned to the sponsor.

11.5.3 Management of Test Vaccine

The test vaccine should be stored in separate areas by project, and managed by the designated person and cabinet with lock. The safekeeping conditions shall be in line with the storage conditions of test vaccine, and monitored by the monitor. The Warehouse Standing Book for Vaccines should include the amount of vaccines received, the amount of vaccines given to subjects, the amount of vaccines left, or the amount of vaccines lost. The investigators will count all the test vaccines at the end of the trial. When research is finished, the remaining test vaccines are counted and returned to the sponsor.

11.6 Sample Management in Clinical Trials

"Samples for laboratory detection and the immunogenicity samples of the subjects not enrolled shall be disposed as medical waste at the research site after the work on that day, and the sample for neutralizing antibody test shall be disposed as medical waste by the detection unit upon detection. The backup sample shall be not delivered together with the submitted samples, but be kept properly by the investigator until the completion of clinical trial report and then destroyed on site upon the confirmation by the Sponsor. Meanwhile, relevant records shall be kept.

11.7 Storage of Data on Clinical Test

The data in the clinical trial must be kept in accordance with GCP requirements, and the Sponsor, responsible organization and trial site shall keep the clinical trial data for 5 years after the approval of test drug for marketing.

11.8 Ending Criteria for Clinical Trial

- The test samples are sent to the corresponding test organization, and the test report should be issued;
- All subjects complete the required visits, and the original data and documents of the clinical trial are handed over to the archivist for archiving and preservation;
- The remaining number of test vaccines is accurate and the remaining test vaccines are handed over to the sponsor;
- The statistical analysis report and summary report meet the requirements of the sponsor.

12 Ethical Approval

12.1 Review and Approval

The clinical trial protocol should be approved by the local independent ethics committee. The principal investigator submits the clinical protocol and all necessary additional documentation to the independent ethics committee, After the approval of the committee, the investigator provides the sponsor with a certificate of approval from the committee.

12.2 Field Supervision

Throughout the trial, the independent ethics committee should supervise if there's any ethical damage to the subjects and if the subjects obtain treatment or compensation and corresponding medical insurance measures when they are badly influenced by the study. What's more, the independent ethics committee should evaluate the risks borne by the subjects.

12.2.1 Informed Consent and Informed Consent Form

Ensure that the subject enrollment method and the relevant data provided to subjects are comprehensive and understandable, and that the method of obtaining informed consent is appropriate. Throughout the trial, the independent ethics committee should periodically review the progress of the trial and assess the risks and benefits of the subjects.

12.2.2 Potential Risks and Risk Minimization

If an adverse event is identified to be associated with the vaccination (abscess at vaccination site and rash after vaccination), the subjects shall be treated timely in accordance with relevant provisions. In case of life-threatening event, the subject will be escorted to the hospital for treatment immediately and report should be made.

Under strict supervision, the trained, experienced medical personnel could carry out vaccination and collect venous blood in accordance with the rules and procedures, so as to minimize the pain of the subject suffering from vaccination and blood collection (including pain and rare local infection in vein puncture site).

12.2.3 Protection Measures for Subjects

The clinical trial should be performed in centers for disease control and prevention with vaccination qualification at county or municipal level. The sponsor should assess the study site in strict accordance with the GCP requirements prior to the start of the trial. The environmental and facilities of the test site should meet the requirements stipulated in Guidelines for Quality Management in Clinical Trial of Vaccines (Trial). An emergency plan for prevention and handling of damages and emergencies of subjects should be made on test site. In physical examination room and blood collection room, qualified and experienced doctors and nurses should be in place to strictly follow the inclusion/exclusion criteria and to collect blood smoothly. Proper first-aid facilities, equipment and medicines should be available in the emergency room and emergency physicians should be qualified and competent. After adverse events occur at test site, subjects should be treated immediately in an emergency room on the site and sent to contracting hospitals by ambulances on site after the condition is stable if emergency hospitalization is required. Ambulances should be equipped with the necessary first aid facilities and drugs. The trial site should sign a Green Channel Agreement with local county-level or higher general hospitals. During the enrollment of subjects, contracting hospitals should be notified for timely treatment. Staff responsibilities should be clarified. Contact numbers and rescue routes should be available to ensure the timely treatment of sudden adverse events and the effective contact between the subject and investigator so that any adverse events are promptly reported and dealt with. When subjects experience serious adverse events and need to be hospitalized for emergency treatment, contracting hospitals can provide green channel services including medical treatment, hospitalization and medical security to ensure that subjects can be treated in time. The investigator follows the progress of the events and completes the investigation records until the end of the serious adverse events.

12.3 Confidentiality

The subjects' privacy should be kept during the study and the collection of biological samples, as well as during the reporting and publication of the study. Only subject code, sample number, collection time and test index are recorded for the test sample. It is strictly restricted that only principal test personnel can obtain electronic and printed copies.

13 Modification of Clinical Trial Protocol

After the sponsor and investigator have signed the clinical trial protocol, if there are any modifications to the protocol, all protocols modified should be re-signed and dated by the principal investigator and sponsor, with the protocols not modified attached.

All modified protocols should be reported to the independent ethics committee and approved by the independent ethics committee before being executed. When a protocol is modified, it should be pointed out whether it is necessary to modify the informed consent and electronic CRF form.

14 Disclosure and Publication of Data

After the completion of this clinical trial, if the results of the trial need to be disclosed and/or published, the positive results will be disclosed and/or published together with the negative results.

15 References

1. State Administration for Market Regulation, Provisions of Drug Registration, 2020.
2. National Medical Products Administration, *Good Clinical Practice*, 2020.
3. The former China Food and Drug Administration, Good Clinical Practice, 2003.
4. The former China Food and Drug Administration, Technical Guidelines for Clinical Trial of Vaccines, 2004.
5. The former China Food and Drug Administration, Guidelines for Quality Management in Clinical Trial of Vaccines (Trial), 2013.
6. Center for Drug Evaluation. Technical Guidelines for Research and Development of Vaccines for Prevention of COVID-19 (Trial). March 2020.
7. Lu R, Zhao X, Li J, Niu P, Yang B, Wu H, Wang W, Song H, Huang B, Zhu N, et al. Genomic characterisation and epidemiology of 2019 novel coronavirus: implications for virus origins and receptor binding. Lancet (London, England). 2020; 6736(20): 1-10.
8. Chen Y, Liu Q, Guo D. Coronaviruses: genome structure, replication, and pathogenesis. Journal of medical virology. 2020: 0-2.
9. Information Office of the State Council, Press Conference on Joint Prevention and Control of Novel Coronavirus–Infected Pneumonia. Beijing, January 26, 2020.
10. China's Novel Coronavirus Pneumonia Diagnosis and Treatment Plan (Provisional 7th Edition)
11. Holshue ML, DeBolt C, Lindquist S, et al. First Case of 2019 Novel Coronavirus in the United States. N Engl J Med. 2020 Mar 5; 382(10): 929-936. .
12. Special Expert Group for Control of the Epidemic of Novel Coronavirus Pneumonia of the Chinese Preventive Medicine Association. An update on the epidemiological characteristics of novel coronavirus pneumonia (COVID-19). Chinese Journal of Epidemiology, 02/14/2020.
13. https://www.who.int/emergencies/diseases/novel-coronavirus-2019/situation-reports.
14. Department Of Diseases Control, National Health Commission of People's Republic of China. Joint Investigation Report of China-WHO on Novel Coronavirus Pneumonia (COVID-19).
15. National Medical Products Administration, Guidelines for Adverse Event Classification Standards for Clinical Trials of Preventive Vaccines, 2019.
16. The former National Medical Products Administration Institute, Guidance on Graded Standard of Adverse Effect in Clinical Trial for Prevention, 2005.
17. Division of microbiology and infections diseases (DMID) adults toxicity table. November 2007. NIH.
