# Supplementary material for: Safety and immunogenicity following a homologous booster dose of CoronaVac in children and adolescents
Source: Nat Commun. 2022 Nov 14;13:6952. doi: 10.1038/s41467-022-34280-y (PMC9663200; doi:10.1038/s41467-022-34280-y)
Supplement: Supplementary file 3 — Reporting Summary [file 41467_2022_34280_MOESM3_ESM.pdf]

nature portfolio

Corresponding author(s): Qiang Gao; Yuliang Zhao

Last updated by author(s): Oct 14, 2022

Reporting Summary

Nature Portfolio wishes to improve the reproducibility of the work that we publish. This form provides structure for consistency and transparency in reporting. For further information on Nature Portfolio policies, see our Editorial Policies and the Editorial Policy Checklist.

Statistics

For all statistical analyses, confirm that the following items are present in the figure legend, table legend, main text, or Methods section.

n/a	Confirmed	
☐	☒	The exact sample size (*n*) for each experimental group/condition, given as a discrete number and unit of measurement
☒	☐	A statement on whether measurements were taken from distinct samples or whether the same sample was measured repeatedly
☐	☒	The statistical test(s) used AND whether they are one- or two-sided<br>*Only common tests should be described solely by name; describe more complex techniques in the Methods section.*
☒	☐	A description of all covariates tested
☒	☐	A description of any assumptions or corrections, such as tests of normality and adjustment for multiple comparisons
☐	☒	A full description of the statistical parameters including central tendency (e.g. means) or other basic estimates (e.g. regression coefficient) AND variation (e.g. standard deviation) or associated estimates of uncertainty (e.g. confidence intervals)
☐	☒	For null hypothesis testing, the test statistic (e.g. *F*, *t*, *r*) with confidence intervals, effect sizes, degrees of freedom and *P* value noted<br>*Give P values as exact values whenever suitable.*
☒	☐	For Bayesian analysis, information on the choice of priors and Markov chain Monte Carlo settings
☒	☐	For hierarchical and complex designs, identification of the appropriate level for tests and full reporting of outcomes
☐	☒	Estimates of effect sizes (e.g. Cohen's *d*, Pearson's *r*), indicating how they were calculated

Our web collection on statistics for biologists contains articles on many of the points above.

Software and code

Policy information about availability of computer code

Data collection	In this trial, Electronic Data Capture (RIEHEN version:2.1.1610) is used to establish the electronic CRF. As an important component of the clinical trials and research reports, the electronic CRF is used to record clinical trial data.
Data analysis	SAS (Version 9.4, SAS Institute Inc., Cary, USA) for all analyses.<br>The SAS code for the main analysis is available on GitHub at https://github.com/wanglei365/sinovac_1003_antibody.

For manuscripts utilizing custom algorithms or software that are central to the research but not yet described in published literature, software must be made available to editors and reviewers. We strongly encourage code deposition in a community repository (e.g. GitHub). See the Nature Portfolio guidelines for submitting code & software for further information.

Data

Policy information about availability of data

All manuscripts must include a data availability statement. This statement should provide the following information, where applicable:

- Accession codes, unique identifiers, or web links for publicly available datasets
- A description of any restrictions on data availability
- For clinical datasets or third party data, please ensure that the statement adheres to our policy

The study protocol is available in the Supplementary Material. The individual participant-level data that underlie the results reported in this article will only be shared after de-identification (text, tables, figures, and supplementary). This clinical trial is ongoing, and all the individual participant data cannot be available until

the immune persistence evaluation is conducted. Source data underlying all figures are provided with this paper. Researchers who provide a scientifically sound proposal will be allowed to access to the de-identified individual participant data. These proposals will be reviewed and approved by the sponsor, investigators and collaborators on the basis of scientific merit. To gain access, data requestors will need to sign a data access agreement. Proposals should be directed to gaoq@sinovac.com. Source data are provided with this paper.

Human research participants

Policy information about studies involving human research participants and Sex and Gender in Research.

Reporting on sex and gender	During the enrollment period, the subjects were randomized to achieve gender balance. The results were not analysis by gender, so our results apply to both genders. During the booster period, 171 participants in cohort 1 and 175 participants in cohort 2 voluntarily participated in the ongoing study and sign the informed consent. All 346 participants received third dose in overall safety analysis. And immunological endpoints were assessed in the per-protocol population. So there was no self-selection bias in gender. The proportion of males were 51% (88/171) in the cohort 1 and 55% (96/175) in the cohort 2.
Population characteristics	The mean age of participants was 9.2 years (SD 3.7) in cohort 1 and 9.3 years (SD 3.8) in cohort 2.The proportion of males were 51% in the cohort 1 and 55% in the cohort 2.
Recruitment	Recruitment notices will be issued to volunteers who meet the enrollment criteria. The informed consent will be explained to the volunteers in detail. Under the condition of voluntary participation, the volunteers and the study doctors sign the informed consent which is in duplicate, and the copy is reserved by the volunteer.<br>Subjects who are normal in physical examination and screened qualified as per other inclusion/exclusion criteria (Screening No. consists of S and screening order, such as "CS0001") will be enrolled and given Research Number based on enrollment order.<br>Compensation: Participants will not be remunerated for participating in this study. But if there are transportation expenses and blood sample collection, small souvenirs (worth approximately 50 yuan) will be distributed to the participants. At the same time, if participants need to take blood sample, 50-yuan blood sampling subsidy will be distributed to them.
Ethics oversight	The complete study protocol was approved by the ethics committees of Hebei Provincial Centre for Disease Control and Prevention (IRB2020-005).

Note that full information on the approval of the study protocol must also be provided in the manuscript.

Field-specific reporting

Please select the one below that is the best fit for your research. If you are not sure, read the appropriate sections before making your selection.

☒ Life sciences ☐ Behavioural & social sciences ☐ Ecological, evolutionary & environmental sciences

For a reference copy of the document with all sections, see nature.com/documents/nr-reporting-summary-flat.pdf

Life sciences study design

All studies must disclose on these points even when the disclosure is negative.

Sample size	The sample size was determined following requirements of the National Medical Products Administration, China's regulatory authority for vaccines.
Data exclusions	Exclusion Criteria for Subjects<br>(1) Travel / residence history of communities with COVID-19 case reports within 14 days prior to the entry;<br>(2) Contact with SARS-CoV-2 infected persons (positive for nucleic acid detection) within 14 days prior to the entry;<br>(3) Contact patients with fever or respiratory symptoms from communities with case reports within 14 days prior to the entry;<br>(4) Two or more cases of fever and / or respiratory symptoms in a small area, such as family, office, school class or other places within 14 days prior to the entry;<br>(5) History of SARS-CoV-2 infection;<br>(6) History of asthma, allergy to vaccines or vaccine ingredients, and serious adverse reactions to vaccines, such as urticaria, dyspnea, angioneuroedema;<br>(7) Congenital malformation or developmental disorder, genetic defect, severe malnutrition, etc;<br>(8) Autoimmune disease or immunodeficiency / immunosuppression;<br>(9) Serious chronic disease, serious cardiovascular disease, hypertension and diabetes that cannot be controlled by drugs, hepatorenal disease, malignant tumor, etc;<br>(10) Serious nervous system disease (epilepsy, convulsion or convulsion) or psychosis;<br>(11) Thyroid disease or history of thyroidectomy, spleenlessness, functional spleenlessness, spleenlessness or splenectomy resulting from any condition;<br>(12) Diagnosed abnormal blood coagulation function (eg, lack of blood coagulation factors, blood coagulopathy, abnormal platelets) or obvious bruising or blood coagulation;<br>(13) Immunosuppressive therapy, cytotoxic therapy, inhaled corticosteroids (excluding allergic rhinitis corticosteroid spray therapy, acute non-complicated dermatitis superficial corticosteroid therapy) in the past 6 months;<br>(14) Abnormal hematological and biochemical laboratory test results beyond the reference value range in physical examination (only applicable to phase I clinical trial)

1) Blood routine indexes: white blood cell count, hemoglobin, platelet count;
2) Blood biochemical indexes: alanine aminotransferase (ALT), aspartate aminotransferase (AST), total bilirubin (TBIL), creatinine (CR), fasting blood glucose;
3) Urine routine index: urine protein (PRO)
(15) Long history of alcohol or drug abuse;
(16) Receipt of blood products in the past 3 months;
(17) Receipt of other investigational drugs in the past 30 days;
(18) Receipt of attenuated live vaccines in the past 14 days;
(19) Receipt of inactivated or subunit vaccines in the past 7 days;
(20) Acute diseases or acute exacerbation of chronic diseases in the past 7 days;
(21) Axillary temperature >37.0°C;
(22) Under Pregnancy (including positive urine pregnancy test) or breast-feeding period and plan to prepare for pregnancy within 3 months;
(23) According to the investigator's judgment, the subject has any other factors that are not suitable for the clinical trial.

Exclusion Criteria for Vaccination with Subsequent Dose
The subjects who experience any of events in the following (1) to (5) are forbidden to continue vaccination, but they can continue other study steps according to the investigator' judgement. For the subjects who experience any of the events in the following (6) to (7), the investigator will judge whether vaccination will be continued. For the subjects who experience any of the events in the following (8) to (11), the vaccination can be delayed within the protocol-permitted time window.
(1) Similar vaccines other than the investigational vaccines were used during the study;
(2) Any serious adverse reactions which have a causal relationship with the vaccination;
(3) Severe anaphylaxis or hypersensitivity after vaccination (including urticaria/rash appears within 30 minutes after vaccination);
(4) Any confirmed or suspected autoimmune disease or immunodeficiency disease, including human immunodeficiency virus (HIV) infection;
(5) Become pregnant after last vaccination (including positive urine pregnancy test);
(6) Acute or newly onset chronic disease after vaccination;
(7) Other reactions (including severe pain, severe swelling, severe limitation of movement, persistent high fever, severe headache or other systemic or local reactions) judged by the investigators;
(8) Acute diseases occur during vaccination (acute disease means moderate or severe disease with or without fever);
(9) Axillary temperature >37.2°C(aged >14 years) or >37.4°C(aged 3~14 years) during vaccination;
(10) Have vaccinated with subunit vaccine or inactivated vaccine within 7 days, immuned with live attenuated vaccine within 14 days;
(11) According to the investigator's judgment, the subject has any other factors that affect vaccination.

Replication

This study represents the phase II clinical trial about the immune response induced by CoronaVac, an inactivated vaccine, no replication is performed in this study. We have the protocol with the manuscript to ensure the reproducibility of this study.

Randomization

Randomisation codes were generated by the randomisation statistician by means of block randomisation using SAS software version 9.4. The randomisation code was allocated to each participant in sequence in the order of enrolment, and then the participants received the investigational products labelled with the same code.

Blinding

Concealed random group allocations and blinding codes were kept in signed envelopes. Investigators, participants, and laboratory staff were masked to group allocation.

Reporting for specific materials, systems and methods

We require information from authors about some types of materials, experimental systems and methods used in many studies. Here, indicate whether each material, system or method listed is relevant to your study. If you are not sure if a list item applies to your research, read the appropriate section before selecting a response.

Materials & experimental systems

n/a	Involved in the study	
☒	☐	Antibodies
☐	☒	Eukaryotic cell lines
☒	☐	Palaeontology and archaeology
☒	☐	Animals and other organisms
☐	☒	Clinical data
☒	☐	Dual use research of concern

Methods

n/a	Involved in the study	
☒	☐	ChIP-seq
☒	☐	Flow cytometry
☒	☐	MRI-based neuroimaging

Eukaryotic cell lines

Policy information about cell lines and Sex and Gender in Research

Cell line source(s)

The Vero cells used in neutralization assay are obtained from kidney tissue of adult African green monkey (W.H.O.VERO SEED LOT 10-87), which was the 10th generation passaged from original ATCC Vero CCL-81.

Authentication

Cell line was authenticated by cell morphology examination and species identification (PCR).

Mycoplasma contamination

Cell line was confirmed to be tested negative for mycoplasma by culture method and indicator cell culture medium (DNA staining).

Commonly misidentified lines
(See ICLAC register)

No.

Clinical data

Policy information about clinical studies
All manuscripts should comply with the ICMJE guidelines for publication of clinical research and a completed CONSORT checklist must be included with all submissions.

Clinical trial registration

NCT04551547

Study protocol

Study protocols are provided in supplementary files.

Data collection

This study was carried out based on the double-blind, randomised, placebo-controlled, phase 2 clinical trial initiated on December 12, 2020, in healthy children and adolescents aged 3-17 years in Zanhuang County, Hebei Province. Between December 12 and December 20, 2020, 515 individuals were screened 480 participants were enrolled in phase 2 trial, of whom 96 were allocated to the placebo group, and 384 were allocated to the 1.5 µg or 3.0 µg vaccine group. Between November 13, 2021 and February 11, 2022, All 346 (90%) participants who received the third dose were included in the safety analyses, and 337 (88%) were included in the per-protocol population for the immunogenic evaluation on day 28 after the third dose.

Outcomes

The primary endpoint of this study is safety and immunogenicity following a third dose of CoronaVac.
The secondary endpoint of this study is antibody persistence following the primary two-dose schedule.

Exploratory endpoint for booster dose
-Seropositive rate, GMT and GMI of neutralizing antibody against Omicron 28 days after booster dose.
-Seropositive rate and GMT of neutralizing antibody against Prototype strain (CZ strain) and Omicron strain 6 and 12 months after booster dose.
Exploratory endpoint for primary immunization
Seropositive rate and GMT of neutralizing antibodies 3, 6 ,10 and 12 months after the second dose.
